# Effectiveness of Motivational Interviewing on adult behaviour change in health and social care settings: A systematic review of reviews

**DOI:** 10.1371/journal.pone.0204890

**Published:** 2018-10-18

**Authors:** Helen Frost, Pauline Campbell, Margaret Maxwell, Ronan E. O’Carroll, Stephan U. Dombrowski, Brian Williams, Helen Cheyne, Emma Coles, Alex Pollock

**Affiliations:** 1 School of Health and Social Care, Edinburgh Napier University, Sighthill Court, Scotland, United Kingdom; 2 Nursing, Midwifery, Allied Health Professional Research Unit (NMAHP-RU), Glasgow Caledonian University, Glasgow, United Kingdom; 3 Nursing, Midwifery, Allied Health Professional Research Unit (NMAHP-RU), School of Health Sciences, University of Stirling, Stirling, Scotland, United Kingdom; 4 School of Health Sciences, Division of Psychology, University of Stirling, Stirling, Scotland, United Kingdom; Brown University, UNITED STATES

## Abstract

**Background:**

The challenge of addressing unhealthy lifestyle choice is of global concern. Motivational Interviewing has been widely implemented to help people change their behaviour, but it is unclear for whom it is most beneficial. This overview aims to appraise and synthesise the review evidence for the effectiveness of Motivational Interviewing on health behaviour of adults in health and social care settings.

**Methods:**

A systematic review of reviews. Methods were pre-specified and documented in a protocol (PROSPERO–CRD42016049278). We systematically searched 7 electronic databases: CDSR; DARE; PROSPERO; MEDLINE; CINAHL; AMED and PsycINFO from 2000 to May 2018. Two reviewers applied pre-defined selection criteria, extracted data using TIDIER guidelines and assessed methodological quality using the ROBIS tool. We used GRADE criteria to rate the strength of the evidence for reviews including meta-analyses.

**Findings:**

Searches identified 5222 records. One hundred and four reviews, including 39 meta-analyses met the inclusion criteria. Most meta-analysis evidence was graded as low or very low (128/155). Moderate quality evidence for mainly short term (<6 months) statistically significant small beneficial effects of Motivational Interviewing were found in 11 of 155 (7%) of meta-analysis comparisons. These outcomes include reducing binge drinking, frequency and quantity of alcohol consumption, substance abuse in people with dependency or addiction, and increasing physical activity participation.

**Conclusions:**

We have created a comprehensive map of reviews relating to Motivational Interviewing to signpost stakeholders to the best available evidence. More high quality research is needed to be confident about the effectiveness of Motivational Interviewing. We identified a large volume of low quality evidence and many areas of overlapping research. To avoid research waste, it is vital for researchers to be aware of existing research, and the implications arising from that research. In the case of Motivational Interviewing issues relating to monitoring and reporting fidelity of interventions need to be addressed.

## Introduction

There is overwhelming epidemiological evidence that health behaviour such as smoking, substance abuse (drugs and alcohol), physical inactivity, and unhealthy eating are associated with increased morbidity and mortality. The cost to the UK NHS for diseases associated with poor diet, physical inactivity, smoking, alcohol and obesity are estimated to be in excess of £12 billion [1]. The challenge of addressing unhealthy lifestyle choice is complex and requires sustained behaviour change. The UK NICE (2014) guidelines [2] recommend a range of behaviour change approaches, guided by a taxonomy of interventions [3], aimed at changing health-related behaviour of individuals, communities or whole populations.

Motivation to change is a key component of the behaviour change process as it guides and maintains goal-related behaviour [4]. One approach to change motivation and subsequent behaviour is Motivational Interviewing, introduced by William Miller in 1983 to help people with alcohol problems change their drinking behaviour [5]. The approach was developed further in the 1990s into “A collaborative conversation style for strengthening a person’s own motivation and commitment to change” [5]. Motivational Interviewing aims to explore and resolve ambivalence that people might have about health behaviour in favour of change. It encourages people to say why and how they might change and pertains both to a style of relating to others and a set of skills to facilitate that process. The four overlapping processes involve: 1) engaging in a working relationship; 2) focusing on a problem to change; 3) evoking the person’s desire to change; 4) planning the change [5]. In 1997 an international organisation of trainers established ‘The Motivational Interviewing Network of Trainers (MINT)’ with an aim to improve the quality and effectiveness of counseling and consultations for professional delivering Motivational Interviewing. The organisation has grown to represent 35 countries and 26 languages, which demonstrates the global popularity of this intervention. Some reviews report positive outcomes for Motivational Interviewing and suggest it could be useful for a wide range of behavioural and health problems [6–9] whilst others are more cautious in their conclusions and recommendations [10–12].

Many different health care professionals and other groups are using behaviour change interventions including Motivational Interviewing to help people change or adapt their behaviour. However, it is unclear for which behavioural problems and populations Motivational Interviewing is most beneficial, or in some cases, where there is evidence of no effect or possible harm. This overview aims to identify, appraise and synthesise the review evidence for the effectiveness of Motivational Interviewing on health behaviour of adults in a wide range of health and social care settings to answer the following question;

What is the strength and quality of the current evidence to support the use of Motivational Interviewing to change adult behaviours in health and social care settings?

This question is important to guide health care professionals, researchers and other stakeholders to the most effective and worthwhile interventions for patients.

## Methods

### Design

We conducted a systematic review of existing reviews (referred to as an overview [13]). An overview synthesises the evidence from more than one systematic review at a variety of different levels, including the combination of different interventions, different outcomes, or people from different populations with different conditions.

### Search methods

We systematically searched the following electronic databases from January 2000 to 28th May 2018; Cochrane Database of Systematic Reviews (CDSR); Database of Reviews of Effects (DARE); PROSPERO (an international prospective register of systematic reviews); MEDLINE; CINAHL; AMED and PsycINFO. The search string was adapted for each database. (See Appendix 1 for Medline search). A comprehensive search combined key terms using Boolean operators (e.g. AND, OR) for: Intervention (e.g. "motivational interviewing," "motivational enhancement") and Review type (e.g. "systematic review," "meta-analysis, " "review literature, " "qualitative systematic review," "evidence synthesis" OR "realist synthesis", "qualitative AND synthesis", "meta-synthesis* OR meta synthesis* OR metasynthesis", "meta-ethnograph* OR metaethnograph* OR meta ethnograph*", "meta-study OR metastudy OR meta study"). Truncated forms of these terms and alternative spellings were included. To be eligible for inclusion, reviews met the following criteria:

#### Inclusion criteria

- Reviews using structured, pre-planned methods to synthesise research studies addressing a clearly defined topic or research question (which could comprise either quantitative, qualitative or mixed methodology)
- Published from January 2000
- Interventions described as Motivational Interviewing or Motivational Enhancement Therapy (MET) delivered in any format (e.g. face to face, online, group, text or telephone)
- English language
- Interventions focused on adults.

#### Exclusion criteria

- Letters, commentaries, expert opinion, theoretical and “non-systematic” or unstructured reviews e.g. reviews without an aim that did not clearly describe the search strategy, selection criteria and quality assessment employed.
- Reviews focused solely on children and adolescents under the age of 18 years
- Reviews focused on Motivational Interviewing intervention to change professional or organisational group behaviour.
- Reviews focused on combined psychological interventions e.g. Motivational Interviewing combined with Cognitive Behavioural Therapy.

### Identification of studies

Members of the review team (PC / SM) ran the search strategy and then examined all titles to exclude clearly irrelevant papers. Two reviewers (PC and HF) independently reviewed the abstracts of all potential records identified from the electronic searches and excluded those not meeting the inclusion criteria. Inter-rater reliability was assessed for agreement of abstract screening.

Two reviewers (PC and HF) independently assessed full papers for all potentially relevant reviews. Full text papers ranked as irrelevant by both reviewers were excluded at this stage of the screening process. The final selection of full text papers (judged as relevant or unsure) were discussed at a consensus meeting, with a third reviewer (MM or AP) as required.

### Data extraction

Three reviewers (PC, HF and EC) independently extracted the following information: review question or aims; types of studies included; characteristics of participants and numbers included; interventions details. The TIDieR framework[14] was used to guide reporting of interventions components and comparators. Two reviewers (HF and PC) checked all the extracted data and discussion between the two reviewers resolved any disagreement; with assistance from a third reviewer (AP) when necessary. A data extraction form (excel) specifically developed by the overview author team was used to collate the data.

### Categorisation of reviews

Two reviewers (PC and HF) categorised each review into one of four of the following domains depending on the focus of the review.

Domain 1: Stopping or preventing an unhealthy behaviour

Domain 2: Promoting healthy behaviour for a specific problem

Domain 3: Behaviour change for multiple health related problems and /or multiple behaviour problems

Domain 4: Behaviour change in specific settings

Reviews in Domain 1 and 2 were then sub-grouped by HF and PC according to the main health behaviour or problem.

### Assessment of quality of reviews

Two reviewers (HF and PC) independently assessed the methodological quality of included reviews using the ROBIS tool [15]. Any disagreement was resolved through discussion between the two reviewers. The tool covers four domains to detect bias in systematic reviews relating to study eligibility criteria; identification and selection of studies; data collection and study appraisal; synthesis and findings. The full result of assessment of bias aids transparency and aims to help researchers judge risk of bias in the review process, results and conclusions.

### Meta-analyses data extraction

One reviewer (PC) extracted comparative data for individual and combined outcomes from any review that included meta-analyses. Data exploring effectiveness of Motivational Interviewing as the main intervention compared with any other intervention or control was extracted. One reviewer (HF) checked the data entry.

This included the following data: Number of trials and participants in the meta-analysis; Measure of effect (e.g. effect size, mean difference, standardised mean difference, relative risk); Measure of variability (95% confidence intervals) and Measure of heterogeneity (I-squared).

Three reviewers (AP, PC and HF) checked the quality assessment of individual studies reported in the reviews and considered the results when grading the evidence. We used the GRADE (Grades of Recommendation, Assessment, Development, and Evaluation) criteria to assess whether the quality of the evidence presented in the meta-analyses was high, moderate, low or very low [16] for all available comparator data within each review. This involved judgement of risk of bias relating to study design, imprecision, inconsistency, indirectness, and publication bias [17]. In addition, one reviewer (PC) extracted any data that included exploration of moderator variables and tabulated effect size for each comparator.

### Meta-analysis synthesis

For reviews including a meta-analysis two reviewers (PC and HF) independently checked the overlap in studies within all the reviews and resolved any uncertainty through discussion. We excluded data superseded by a more up-to-date review (e.g. where a Cochrane review had been updated while we were conducting the overview), or in cases where an overlapping review was conducted with the same review question, we selected the higher quality review judged using the ROBIS quality assessment tool [15]. We tabulated the intervention, comparison, outcome, number of studies and participants’ data relating to effectiveness and the GRADE of evidence [18]. Using the data relating to effectiveness we noted whether there was statistically significant evidence of benefit or harm for each outcome reported in the meta-analyses, or if there was no evidence of benefit or harm (no statistically significant effect).

### Narrative review synthesis

For all systematic reviews without meta-analysis data (defined as narrative reviews), we summarised key findings. We systematically documented and explored the conclusions reported by the authors of the reviews. Where these reviews included overlapping aims and outcomes, we compared conclusions; where there was a discrepancy in conclusions, we focused conclusions of the most up-to-date and highest quality reviews (judged using ROBIS) [15]. We considered whether these were in agreement with the results of any related meta-analyses reported in other reviews and focused our conclusions on the most up-to-date and high quality data.

## Results

**Fig 1:**
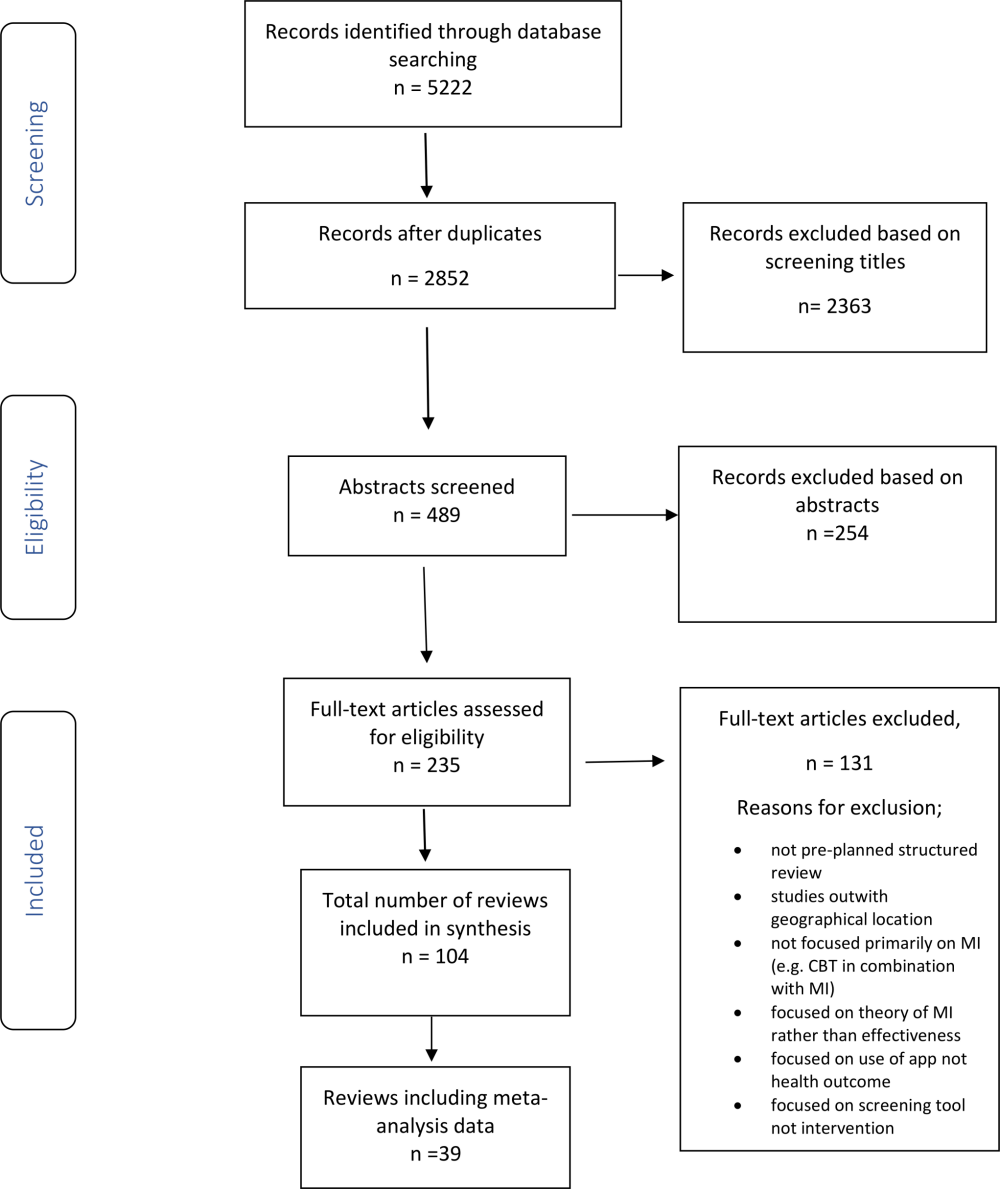
PRISMA Study flow diagram. MI = Motivational Interviewing; CBT = Cognitive Behavioural Therapy.

The search identified 5222 records; we screened 2852 titles and removed 2363 obviously irrelevant records after removing duplications. Two reviewers screened 489 abstracts and 235 full text articles, excluded 131 reviews and extracted data from the remaining 104 reviews. The inter-rater reliability for abstract screening was 92%. The PRISMA flow diagram (Fig 1) shows the flow of literature through the searching and screening process.

### Description of included reviews

**Fig 2:**
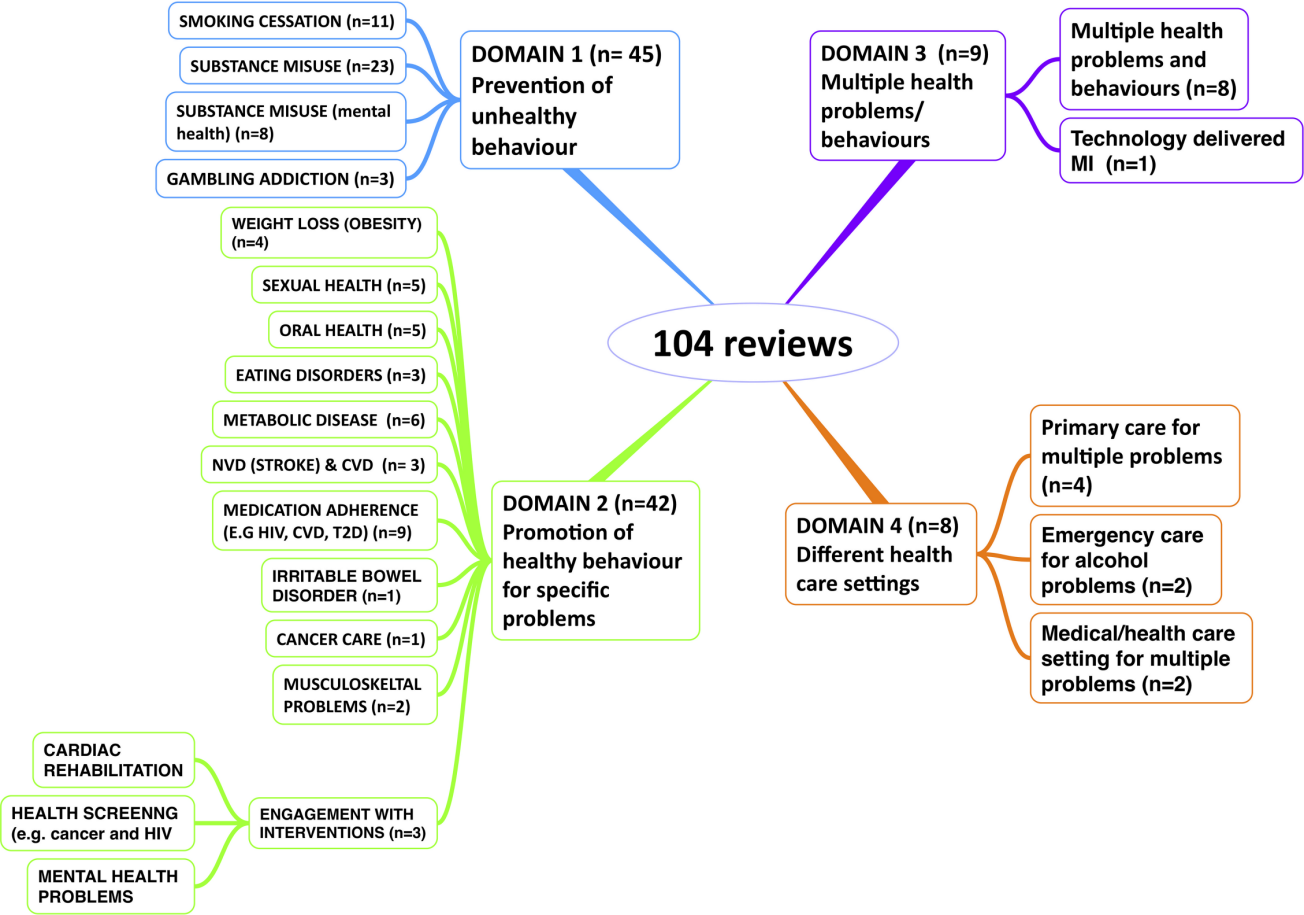
Number of reviews in each domain.

Two reviewers categorised the reviews into four domains. The number of reviews in each domain are represented in Fig 2.

Domain 1. Stopping or preventing an unhealthy behaviour including smoking cessation (n = 11) [11, 12, 19–43], substance misuse for general population (alcohol and drugs) (n = 23) [28, 29, 38–58], substance misuse for people with mental health problems (n = 8) [31, 33, 35–37, 59–61] and people with gambling addiction (n = 3)[7, 62, 63] (Total = 45).

Domain 2. Promoting healthy behaviour for a specific problem including; management of oral health (n = 5) [64–68], eating disorders (n = 3) [10, 69, 70], weight loss management (n = 4) [71–74], management of metabolic disease (Type 2 diabetes) (n = 6) [75–80], management of neurovascular (stroke) and cardiovascular disease (n = 3) [81–83], management of sexual health (n = 5) [84–88], adherence to medication (n = 9) [89–97] and engagement with interventions; cardiac care [98], health screening [99] and mental health interventions [100] (n = 3), cancer care (n = 1) [101], musculoskeletal problems [102, 103] (n = 2), irritable bowel disorder[104] (n = 1).

Domain 3. Behaviour change for multiple health related problems and /or multiple behaviour problems (n = 9) including one recent review of Technology Delivered Motivational Interviewing (TDMI)[105] and eight reviews focused on various health problem such as excess drinking, smoking, and physical inactivity [8, 9, 106–111].

Domain 4. Behaviour change in specific settings (n = 8) including emergency care settings [112, 113](n = 2), primary care [114–117](n = 4), medical care settings for multiple problems [6, 118](n = 2).

### Domain 1: Reviews focused on interventions aimed at preventing unhealthy behaviour

#### Smoking cessation

Of the 11 reviews [11, 12, 19–27], two reviews focused on reducing exposure of smoke to children [11, 20], one on smoking during pregnancy [19], three on general smoking cessation [22–24], two were carried out in emergency care settings [25, 26], One review was updated from an earlier review of Motivational Interviewing to support smoking cessation [119] with the addition of 14 studies since 2010 [12]. One review focused on smokeless tobacco users although only one out of 34 trials included Motivational Interviewing [21].

#### Substance misuse

Thirty-one reviews assessed substance misuse/abuse of which 13 focused primarily on alcohol related problems [28, 39, 40, 43–46, 49, 50, 52, 53, 55, 58]. Reviews in this domain included different populations and problems [29, 38, 41, 42, 48, 56, 57] [53, 54]; both alcohol and drug abuse users[56]; young adults [39]; pregnant women and drug use [38], two reviews focused on cannabis use [41, 42]; one focused on offenders and treatment retention [29]. Eight reviews describe substance misuse in people with co-existing mental health disorders [31–37]. Jiang et al (2017) focused on brief non face-to- face interventions e.g. telephone.

#### Gambling behaviour

Three reviews focused on Motivational Interviewing and psychological therapies for gambling addiction [7, 30, 63]. Yakovenko et al (2015) [7] identified eight trials including longer term follow up, Petry (2017) [63] reviewed trials of psychological interventions but identified only 2 trials that included Motivational Interviewing as a stand-alone intervention.

### Domain 2: Reviews focused on interventions aimed at promoting healthy behaviour for a specific problem

#### Oral hygiene behaviour

Five reviews focused on oral hygiene, 3 compared conventional oral hygiene advice with Motivational Interviewing interventions [64, 65, 68]. One compared periodontal therapy alone with Motivational Interviewing and periodontal therapy combined [66], and one included a meta-analysis of psychological treatment for people with poor oral health [67].

#### Eating disorders

Three reviews focused on eating disorders of mainly female participants e.g. Anorexia nervosa and bulimia nervosa [10, 69, 70].

#### Weight management behaviour

Three reviews focused on changing diet and physical activity for weight management in obese adults [71, 72, 74] and one investigated the management of weight gain during pregnancy [73].

#### Management of diabetes

Six reviews focused on the management of people with diabetes. They include reviews focussed on evidence for; improving health behaviour in the management of diabetes [75], promoting glycaemic control [77] and lifestyle modifications programmes for- metabolic risk [78]. Four other reviews categorised in Domain 3 (multiple health problems / behaviours) and Domain 4 (Behaviour change in specific settings) assessed the effectiveness of Motivational Interviewing for diabetes management alongside obesity and other health related problems [71, 91, 114, 118].

#### Management of neurovascular disorders and cardiovascular disease (CVD)

Three reviews focused on behavioural interventions for neurovascular disorders, but the reviews only included 11 trials in total evaluating the effectiveness of Motivational Interviewing. One review investigated Motivational Interviewing for the management of activities of daily living for stroke victims, identifying one study only [81]. Hildebrand (2015) reported one of 39 trials that incorporated Motivational Interviewing into interventions to support occupational therapy for stroke victims [82]. Lee et al (2016) [83] investigated lifestyle modification, physiological and psychological outcomes for people diagnosed with Cardiovascular disease. Overall there is insufficient evidence in this group to make firm conclusions about effectiveness of Motivational Interviewing.

#### Sexual health behaviour

Five reviews focused on promoting safe sexual behaviours [84–88]. Two reviews focused specifically on sexual health in gay men [84, 85]. One review focused on the effectiveness of Motivational Interviewing on contraceptive use in women [87].

#### Adherence to medication

Adherence to medication was assessed for different populations and health problems. Hu et al (2014) assessed interventions including Motivational Interviewing to increase medication adherence in racial and ethnic minority groups [94]. Five reviews assessed medication adherence for patients with HIV [90, 94, 96, 97, 120]. Two recent reviews with meta-analyses assessed the effectiveness of Motivational Interviewing to enhance medication adherence for adults with chronic diseases and health problems [93, 95].

#### Engagement with interventions

Three reviews focused on engagement with a specific intervention [98–100]; one specifically on cardiac rehabilitation. Karmali et al (2014) assessed adherence to cardiac rehabilitation but only one trial of Motivational Interviewing was identified in this review [98]. A review with meta-analysis of outcomes relating to adherence by Lawrence et al (2017) [100] investigated individuals’ uptake of mental health interventions. Miller et al (2017) [99] assessed the efficacy of Motivational Interviewing to improve health screening for various problems e.g. breast screening, uptake of colonoscopy.

In addition, two other reviews grouped in Domain 1 and 2 assessed the effect of Motivational Interviewing on adherence to drug management programmes in offender populations [29] and adherence to treatment for chronic pain [102].

#### Management of musculoskeletal problems

Two reviews focused on musculoskeletal problem [102, 103] with some overlap of trial within the reviews. In the most recent review, Alperstein and Sharp (2016) identified 7 trials focused on pain outcomes and adherence to treatment in adults with various musculoskeletal problems e.g. low back pain, rheumatoid arthritis [102].

#### Management of irritable bowel disorders

One review explored the use of Motivational Interviewing to improve outcomes for people with irritable bowel disorders including quality of life measures [104].

#### Cancer care

One review focused on Motivational Interviewing to address various lifestyle behaviours and health problem associated with cancer such as fatigue, weight problems, and physical activity participation [101].

### Domain 3: Reviews that focused on multiple health related problems and /or multiple behaviour problems

Nine reviews focused on behavioural interventions for people with multiple health problems [8, 9, 105–111]; These included multiple risk factors for cardiovascular disease[110]; diet, exercise, diabetes and oral health[109]; alcohol, drugs, diet and exercise[106, 111]; substance abuse, smoking, HIV risk, diet and exercise[107] multiple behaviour problems[8, 108] and multiple health outcomes [9]. Shingleton et al (2016) evaluated the efficacy of technology delivered Motivational Interviewing interventions in a mixed population from different socioeconomic backgrounds [105].

### Domain 4: Reviews focused on behaviour change interventions in specific settings

Eight reviews reported behaviour change interventions delivered in specific settings [6, 112–118]. One included a combination of healthcare settings [118]; one focused on medical care settings [6]; four were carried out in primary care[114–117]. Merz et al (2015)[113] and Kohler and Hofmann (2015)[112] focused on young adults in emergency care units. In addition, two reviews described in Domain 1 (preventing an unhealthy behaviour) also reported smoking cessation in emergency department settings [25, 26].

### Review characteristics and quality assessment

**Fig 3:**
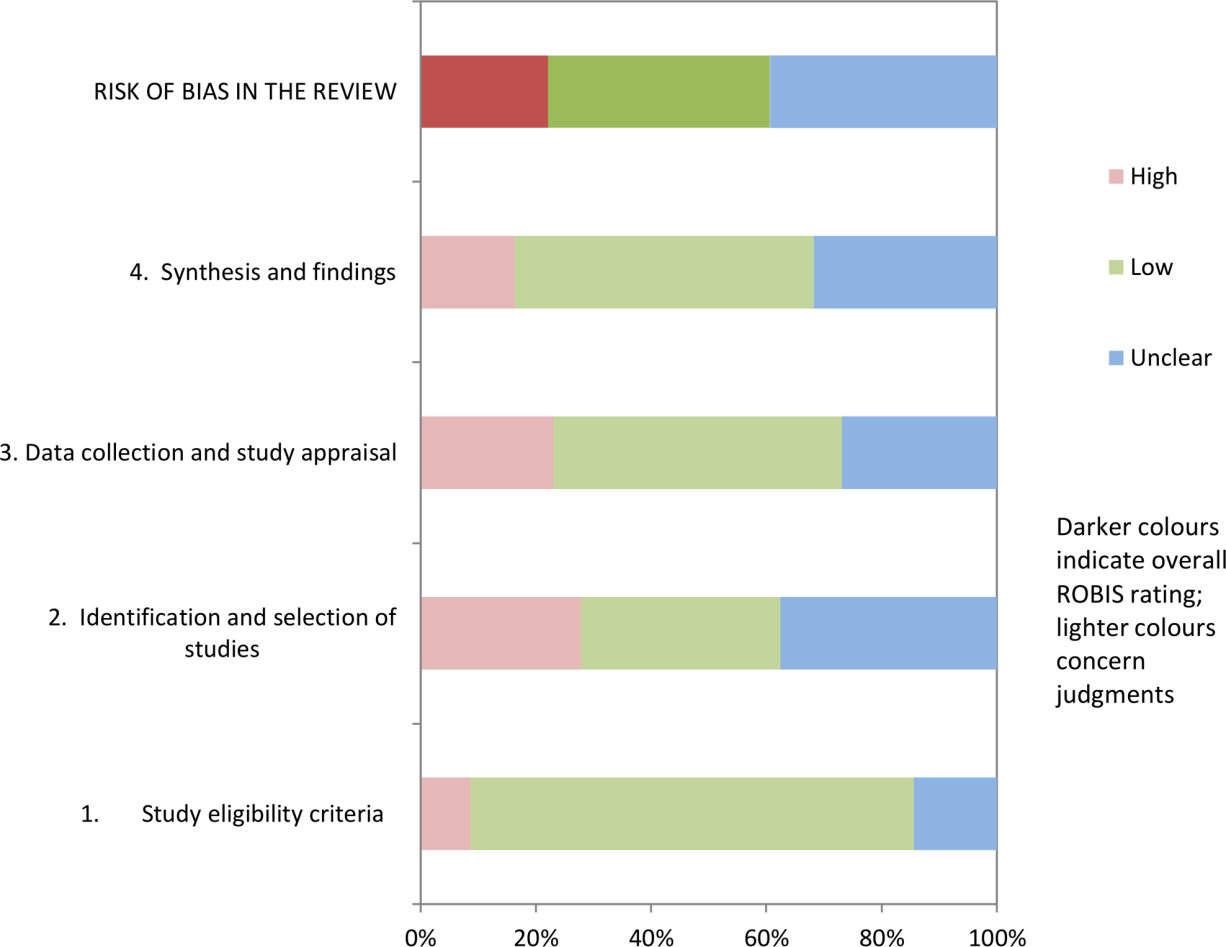
Bar chart summary of ROBIS across included reviews [15].

Tables 1–4 report details of the review characteristics and implications for clinical practice and research. Further details of the interventions using the ‘Template for Intervention Description and Replication (TIDieR) [14] are reported in S1 Table. Of the 104 reviews 40 were judged by two authors (PC and HF) as overall low risk of bias [7, 11, 12, 20, 21, 25–27, 30, 35, 38, 41, 44, 47–49, 51, 53, 54, 56, 57, 59, 65, 71, 81, 83, 84, 89, 91–94, 97, 98, 100, 102, 111, 113–115]. Fig 3 summaries the risk of bias across all reviews. S2 Table reports the assessment of bias for each review individually using the ROBIS tool [15].

**Table 1:** Characteristics of included reviews of Motivational Interviewing (MI) and summary of findings for Domains 1 (Smoking Cessation). Abbreviations: MI = Motivational Interviewing, BMI Brief Motivational Interviewing, RCT = randomised controlled trial, MET = Motivational Enhancement Therapy.

Domain 1: Smoking/Tobacco cessation								
Review author	Objective	Type and Number of studies	Participants	Intervention /*comparisons*	Outcomes	Authors conclusions	Meta-analysis (M-A) or Narrative review (NR) and overall Risk of Bias (ROBIS score)	Implication for clinical practice and research (Interpretation of authors of overview)
Baxi et al (2014)[11]	To determine the effectiveness of interventions aiming to reduce exposure of children to environmental tobacco smoke.	57 controlled trials (n = unclear)	Parents, family members, child care workers and teachers	MI / *Usual care—Placebo*	Primary outcome -children’s exposure to tobacco smoke	Inconclusive	NR (LOW)	. There is moderate quality evidence (assessed by GRADE) that MI interventions provide small beneficial effects in smoking cessation in the short and long term (4–12 months) compared to no treatment. Effects are likely to be small. (See Tables 5 & 6) Further high quality research focusing on training and competency, fidelity, delivery and dose in different settings for specific groups is justified. Windows of opportunity to promote smoking cessation should be investigated further e.g. in specific antenatal groups and during pregnancy
Baxter (2011) [19]	Interventions aimed at smoke-free homes in pregnancy and in the year following childbirth.	1/17 RCT included MI (n = 291)	Pregnant women	MI / *Control group received ‘self-help’ materials via post*.	Exposure levels of environmental nicotine at 6 months in TV room and kitchen	Nicotine levels were significantly lower in MI households	NR (UNCLEAR)	
Behbod et al (2018)[20]	To determine the effectiveness of interventions designed to reduce exposure of children to environmental tobacco smoke, or ETS.	78 RCTs n total. 15 used MI n = >3000	Parents / family members, child care workers, and teachers	MI /BMI /Telephone delivered MI/ *control*, *TAU other psychological interventions*.	Tobacco smoke exposure / clinical symptoms e.g. of asthma	Only 26/78 studies reported benefits. Mixed results for MI. One study reduced children’s asthma symptoms.	NR (LOW)	
Ebbert et al (2015) [21]	To assess the effects of behavioural and pharmacologic interventions for the treatment of smokeless tobacco (ST) use.	RCT 1/34 studies used MI (n = 60)	Adult male ST users	MI/ *Usual care control*, *given information on how to sign up for an 8w cessation class*	Complete abstinence from tobacco use six months or more after the start of the intervention	Only 1 trial of MI and high chance of bias. Insufficient evidence.	M-A (LOW)	
Heckman et al (2010)[22]	To investigate the efficacy of interventions incorporating MI for smoking cessation.	31 RCTs and CRT (n = 9,485)	Mixed adults including pregnant/postpartum women	MI /*Brief advice plus some written materials*.	Primary outcomes: abstinence or reduction in smoking	MI for smoking cessation is effective	M-A (HIGH)	
Hettema et al 2010[23]	To focus solely on smoking cessation and examine potential moderating factors to inform clinical practice guidelines.	31 studies (n = 8165)	Mixed adults of different race and sex	MI /*Another treatment or no treatment control or placebo control"*.	Variable smoking abstinence outcome.	MI significantly outperformed comparison conditions at long-term follow-up	M-A (UNCLEAR)	
Lindson-Hawley et al (2015)[12]	To determine whether or not MI promotes smoking cessation.	28 studies (n = > 16,000)	Mixed population	MI / *brief advice or usual care in the trials*.	Abstinence from smoking after at least six months follow-up	MI effective but possibility of publication or selective reporting bias. 2/28 studies included cost effectiveness but no clear conclusion could be drawn	M-A (LOW)	
Mantler et al (2012)[24]	To compare three different dimensions of MI at facilitating smoking cessation.	17 studies (n = 11600)	Adults between 18 and 64 years	MI strategies / *variable controls e*.*g*. *written materials and education*	Self-reported outcomes and biological measures	Inconclusive	NR (UNCLEAR)	
Pelletier et al (2014)[25]	Effectiveness of smoking cessation interventions for patients in the adult or paediatric emergency care setting.	4 RCTs included MI (n = 74–1044)	Adults in emergency setting	MI plus brochures / *control brochures*	Smoking cessation	Inconclusive	NR (LOW)	
Rabe et al (2013)[26]	To examine the efficacy of Emergency Department–Initiated Tobacco Control	7 RCTs (n = 1,986)	Adults age range from 18–78 years	MI plus booklets /*Usual care*, *brief advice only; brief counselling*	Smoking abstinence	MI increased abstinence up to 12 months.	M-A (LOW)	
Stead et al 2016[27]	To assess the effect of combining behavioural support and medication to aid smoking cessation, compared to a minimal intervention or usual care or usual care.	53 studies, 16 included MI (n = <25000)	Adult smokers. 35 to 65% female participants with average age from low 40’s to mid-50.	MI strategies / *usual care or brief advice or less intensive behavioural support*	Abstinence from smoking after at least six months of follow-up.	Combination of pharmacotherapy with behavioural support improves quit rates compared to no treatment or a minimum intervention.	M-A (LOW)	

**Table 2:** Characteristics of included reviews of Motivational Interviewing (MI) and summary of findings for Domains 1 (Substance misuse and gambling). Abbreviations: MI = Motivational Interviewing, BMI Brief Motivational Interviewing, RCT = randomised controlled trial, MET = Motivational Enhancement Therapy, HAART = Highly Active Antiretroviral Therapies, ETS = Environmental Tobacco Smoke, SUMSM = Substance-using men who have sex with men, BCT = Behaviour change techniques, BZDs = Benzodiazepines, Blood alcohol concentration (BAC).

Domain 1: Substance Abuse (Alcohol and Drugs)								
Review author	Objective	Type and Number of studies	Participants	Intervention / *Comparison*	Outcomes	Authors’ conclusions	Meta-analysis (M-A) or Narrative review (NR) and overall Risk of Bias (ROBIS score)	Implication for clinical practice and research (Interpretation of authors of overview)
Appiah-Brempong, et al (2014) [43]	To assess the effectiveness of MI interventions in reducing alcohol consumption among college students.	13 RCTs (n = 1904)	College students with mean age of 18.1–21.2 across all studies	MI and adaptions of MI underpinned by the key principles/ *Alcohol Education; TAU*	Alcohol consumption	MI is effective in reducing collegiate alcohol consumption when compared to alternative interventions and no intervention.	NR (HIGH)	Moderate quality evidence (assessed by GRADE) that MI is effective in reducing alcohol intake in some populations including young adults <25. The effects are small (See Tables 5 & 6). MI may be beneficial in preventing alcohol abuse for college students and pregnant women but more high-quality research is needed to confirm results. Further high-quality research focusing on training and competency, fidelity, delivery and dose for specific groups is justified. Windows of opportunity to reduce alcohol/drug abuse should be investigated further e.g. in specific antenatal groups and during pregnancy, in offender populations and students at transition to university
Barrio et al (2016) [44]	To systematically assess the efficacy of interventions based on a Patient Centred Care (PCC) health care approach for the management of alcohol use disorders.	36 of 40 studies (n = 16,020). Sample size in each study ranged from 54 to 987	Adults with alcohol use disorders / university students/ people attending ED/ army conscripts	BMI and MI */ no treatment control/ other psychological interventions* / *educational sessions*	Amount and frequency of alcohol consumption, e.g. binge drinking	Trials on PCC interventions based on MI appeared mixed.	NR (LOW)	
Branscum et al (2010) [28]	To make implications for future research initiatives and current health-promoting interventions.	11 studies (N = 1674)	Students; heavy drinkers	MI based interventions / *No treatment control groups or no information for comparisons*.	Alcohol use and drinking problems	MI effective at reducing alcohol use and drinking problems.	NR (HIGH)	
Carey et al (2007) [46]	To summarize the current status of the literature on alcohol abuse and college drinkers.	62 studies published between 1985 to early 2007 (n = 13750)	College students; males (53%) and females (47%) mostly heavy drinkers (65%)	MI techniques were used in 44% of the interventions */no-treatment condition or active comparison*	Alcohol consumption, quantity and typical blood alcohol concentration (BAC)	Individual-level alcohol interventions for college drinkers reduce alcohol use	M-A (UNCLEAR)	
Carey et al (2012) [45]	To compare computer delivered interventions and face to face interventions for MI.	48 (RCT) or quasi-experimental design Face to face (n = 5,237); Computer interventions (n = 32,243).	First-year students and/or heavy drinkers.	Brief MI */ no-treatment condition* *assessment-only*, *55%); active comparison conditions*	Alcohol consumed per week or month and per drinking day, frequency of heavy drinking, peak BAC.	Face to face brief MI is most effective.	M-A (UNCLEAR)	
Chatter et al (2016) [47]	To assess the effectiveness of a broad range of psychosocial and psychological interventions for cannabis cessation in adults.	25 RCTs; 12 MI or MET (n = >4497)	Adults > 8 years who were users of cannabis	MI or MET or BMI/ *waiting list control*, *TAU*, *CBT or other psychological intervention*	Level of cannabis use/dependence	Brief MI improved short-term outcomes at post-treatment in a younger non-clinically dependent population. Results were mixed	NR (LOW)	
Cooper et al (2015) [42]	To systematically review the evidence for the clinical effectiveness of psychological and psychosocial interventions for cannabis cessation in adults who use cannabis regularly.	33 studies; 10 include MI (n = 3700)	18 years + mean age ranged from 18–36 years. People with general and psychiatric disorders.	MI or MET/ *TAU*, *CBT or waiting list control or other treatment*.	Frequency and amount of cannabis use; severity of dependence; motivation to change; level of cannabis-related problems, attendance, retention and dropout rates.	BMI vs other: mixed results due to limited data. BMI better than education control on some but not all outcomes. BMI vs. wait list/AO: some significant differences. BMI significantly better on some outcomes but not all.	NR (LOW)	
Darker et al (2015) [48]	To evaluate the effectiveness of psychosocial interventions for treating Benzodiazepines (BZDs) harmful use, abuse or dependence.	25 RCTs; 4 include MI n = 80)	Opiate dependent population sand non-opiate dependent populations.	MI/MET/ *Treatment as usual (TAU)*	Successful discontinuation of BZDs- post treatment	The effect of MI versus TAU for all the time intervals is unclear. Currently there is insufficient evidence to support the use of MI to reduce BZD use	M-A (LOW)	
Gates et al (2016) [41]	To evaluate the efficacy of psychosocial interventions for cannabis use disorder (compared with inactive control and/or alternative treatment) delivered to adults in an out-patient or community setting	23 RCTs. (15 included MI /MET)	18 years + diagnostic criteria for cannabis abuse or dependence by clinical assessment or were at least near daily cannabis users	MI /MET/ *untreated/minimally treated control or delayed treatment control (DTC)) or a second active psychosocial intervention*	• Severity of cannabis use • Level of cannabis-related problems • Retention in treatment, • Motivation to change cannabis use • Frequency of self-reported other substance intake • Mental health outcomes	The most consistent evidence supports the use of cognitive-behavioural therapy (CBT), motivational enhancement therapy (MET) and particularly their combination for assisting with reduction of cannabis use frequency at early follow-up. No intervention consistently effective at long term follow-up or later.	M-A (LOW)	
Jiang et al (2017) [51]	To synthesize the evidence on the effectiveness of motivational interviewing (MI), delivered in modes other than face-to-face individual counselling, in preventing and treating substance abuse related behaviours.	25 articles (22 RCTs) 57 to 2151, (n = 9920)	Problem drinkers; college students, pregnant women, cannabis users, military personnel.	Telephone, SMS and group MI / *Control of TAU*, *pamphlet*, *nurse–led health promotion*	Smoking frequency, drug use, alcohol intake and frequency	Telephone MI is a promising mode of intervention in treating and preventing substance abuse. The effectiveness of other alternative modes (SMS-based MI, Internet based MI and group MI) remain inconclusive.	NR (LOW)	
Joseph et al (2014) [40]	To compare the efficacy of nurse-conducted brief interventions in reducing alcohol consumption,	11 RCTs 2/11 specifically MI (n = 2676 trial size 134 and 251)	Adult alcohol users identified on the basis of a screening tool score	MI/ *TAU*, *general practitioner or nurse advice to cut down drinking*, *TAU plus a booklet*.	Self-reported alcohol consumption; quantity and frequency of alcohol consumed. Number of ED attendances	For 2 trials that assessed MI 1 found MI to be effective at 12 months. Goodall et al (2008) and Dent et al. (2008) found no difference between MI and usual care.	NR (UNCLEAR)	
Joseph and Basu (2017) [52]	To assess the efficacy of alcohol brief interventions in reducing hazardous or harmful drinking in randomized controlled trials (RCTs) conducted in middle-income countries.	9 RCTs in middle-income countries (n = 3411)	Patients and students, alcohol users.	BMI/ TAU, health education, assessment only	Self-reported drinking	Brief intervention can help reduce self-reported hazardous or harmful alcohol use in primary-care population.	NR (HIGH)	
Klimas et al (2012) [53]	To assess the effects of psychosocial interventions for problem alcohol use in illicit drug users (principally problem drug users of opiates and stimulants)	RCTs and CCTs MI in 2 studies only (n = 443).	Adult (>18 year) problem drug users attending a range of services	MI / *Psychosocial interventions with another therapy years) illicit drug users with concurrent problem alcohol use*	Drug use, Engagement in further treatment, Alcohol-related problems or physical or mental health outcomes	No conclusion can be made due to the paucity of the data and the low quality of the retrieved studies	NR (LOW)	
Foxcroft et al (2014) [49]	To evaluate the effectiveness of MI for the prevention of alcohol and alcohol-related problems in young adults.	66 RCTs (n = 17901)	Young adults <25 yrs. old	MI +feedback element or other non-MI techniques. /*No intervention*, *placebo or TAU*	Alcohol misuse, quantity, frequency and Binge drinking.	No meaningful benefits of MI for the prevention of alcohol misuse. Authors consider effect sizes as too small to be of relevance to policy or practice.	M-A (LOW)	
Gilinsky et al (2011) [50]	To determine whether pregnant women reduced alcohol consumption during pregnancy following interventions delivered during antenatal care.	6 studies. 1 includes MI (n = 40)	Pregnant women mean age 24	MI/ *Other behavioural interventions*	Total alcohol consumption, or the number of days abstinent	In general, methodological quality in all but two studies was poor, limiting the conclusions.	NR (UNCEAR)	
Livingston et al (2012) [54]	To evaluate interventions designed to reduce stigma related to substance use disorders.	Mixed design-(only 1 MI study n = 100)	General public.; 40–45 years	MI/ *Not stated*	Attitudes to Mental Illness Questionnaire	Effective strategies for addressing social stigma include motivational interviewing	NR (LOW)	
McMurran (2009) [29]	To systematically review the evidence of the impact of MI or MET with offender populations.	19 studies including 10 RCTs (n = 40–490)	Varied, mainly adults with alcohol and drug dependency.	MI or MET/ *varied- relaxation techniques*, *non-trained probation offices delivering MI*, *no intervention*	Improved retention to treatment, motivation to change and reduced offending.	Effects were inconsistent, and only a minority of lowest risk of bias RCTs improved both retention with treatment and clinical outcomes.	NR (HIGH)	
Seigers and Carey (2010) [55]	To provide a critical review of the efficacy of brief interventions for alcohol use in college health centres.	8 RCTs 4 uncontrolled studies	College students	MI / TAU/ *no additional treatment) and minimal alternative interventions (e*.*g*., *pamphlets)*.	Alcohol consumption, consequences of drinking, client satisfaction.	Findings support continued use of time-limited, single-session interventions with MI and feedback components.	NR (HIGH)	
Smedslund et al (2011) [56]	To assess the effectiveness of MI for substance abuse on drug use, retention in treatment, readiness to change and number of repeat convictions.	59 RCTs (n = 13,342)	College drinkers, outpatient alcohol clinics, and drink drivers	MET or MI / *no treatment control;* *no treatment as usual; other active intervention for substance abuse*	Extent of substance abuse, retention in treatment, motivation for change, repeat conviction.	MI can reduce the extent of substance abuse compared to no intervention. The evidence is mostly of low quality.	M-A (LOW)	
Tanner-Smith et al (2015) [39]	To examine how much, when, for whom, and for how long brief alcohol interventions may be effective in youth populations.	161 RCTs or quasi RCTS including young adults	Adolescents young adults (age 19–30)	MI and MET/ *no treatment*, *a wait-list control*, *or some form of routine treatment as usual*	Alcohol consumption; alcohol-related problems	Brief alcohol interventions (up to 5hrs) associated with statistically significant post-intervention reductions in alcohol consumption and alcohol-related problem outcomes among young adults.	M-A (UNCLEAR)	
Terplan et al (2007) [57]	To evaluate the effectiveness of psychosocial interventions in pregnant women enrolled in illicit drug treatment programmes	9 RCTs (4 including MI) (n = 266)	Pregnant women, Majority African American, single women.	MI /MET. *Pharmacological intervention*, *placebo or no intervention*, *other psychosocial intervention*	Retention to treatment	There is insufficient evidence to support the use of MI. MI may reduce retention to treatment.	M-A (LOW)	
Terplan et al (2015) [38]	To evaluate the effectiveness of psychosocial interventions in pregnant women enrolled in illicit drug treatment programmes	14 RCTs (5 used MI or MET) Study sizes ranged from 12 to 168 (N = 603)	Pregnant women; women on methadone treatment. mean age for those was approx. 28 years.	Motivational interviewing based (MIB) interventions including MET /*Usual care*, *including pharmacological treatment*, *counselling*, *prenatal care*.	Neonatal outcomes: Time spent in hospital post-delivery. Maternal drug use measured by: Maternal toxicology; Maternal self-reported drug use. Adverse events for the mother.	Little evidence that psychosocial interventions reduce continued illicit drug use in pregnant women enrolled in drug treatment. Overall, the quality of the evidence was low to moderate.	M-A (LOW)	
Vasilaki et al [58]	To examine whether or not MI is (1) more efficacious than no intervention in reducing alcohol consumption; (2) is as efficacious as other interventions.	RCTs (n = 2767)	Dependent or abusive drinkers.	BMI *No treatment or another treatment*.	Standard drinks per week, per day. per drinking occasion.	Brief MI is effective	M-A (UNCLEAR)	
**Domain 1: Substance Misuse in People with Co-existing Mental Health Problems**								
Baker et al (2012) [33]	To determine whether psychological interventions that target alcohol misuse among people with psychotic disorders are effective.	7 RCTs (n = 942)	People with psychotic disorders Mean age 25–45	MI /*Mixed comparison ranging from one 60-minute session to treatment compared with CBT or other psychological treatments*	Units of alcohol per day/week in the previous month from baseline to first follow up.	Poor quality studies included. No clear difference between outcome of alcohol consumption between MI and comparison group of CBT and Brief educational intervention	NR (UNCLEAR)	No moderate quality evidence of effectiveness (assessed by GRADE). Narrative reviews suggest there is some support that MI may help reduce substance abuse in the short term, and there is potential for effects of MI for reducing substance misuse for people with mental health problems but further high quality research is needed, focusing on long-term outcomes. Lower quality reviews conflict with meta-analysis data. Further research on MI for people in psychiatric settings with mental illness to address addiction and depression is justified.
Baker, et al (2012) [34]	To determine whether psychological interventions that target alcohol misuse among people with co-occurring depressive or anxiety disorders are effective.	8 RCTs (3 included MI) (n = 318).	Mixed sex, Inpatients and outpatients with various diagnosis	MI / *brief intervention*, *information pack control*, *attention control group*	Alcohol use depressive mental health outcomes,	There is accumulating evidence for effectiveness of MI (and CBT) for people with co-occurring alcohol and depressive or anxiety disorders	NR (UNCLEAR)	
Boniface et al (2018) [59]	To review the evidence on the effect of brief interventions (BIs) for alcohol among adults with risky alcohol consumption and comorbid mental health conditions.	17 RCTs; 9 included MI (n = > 1530)	Adults with common and severe mental Health problems and illness.	BMI or MET/*control*, *TAU*, *education leaflet*, *CBT*	Alcohol consumption measured by self-report, including quantity or frequency measures, or composite scores.	Evidence is mixed regarding the effects of BIs for alcohol in participants with comorbid mental health conditions. Non-specific relating to MI.	NR (LOW)	
Cleary et al (2009) [35]	To assess current evidence for the efficacy of psychosocial interventions for reducing substance use, improving mental state and encouraging treatment retention, among people with dual diagnosis	54 studies. 9 included MI	People with severe mental illness	MI/ *TAU; psycho-education*, *self-help booklet; psycho-education*	Addiction severity index, alcohol use inventory; Beck depression index; mental health outcomes	These results indicate the importance of MI in psychiatric settings for the reduction of substance use, at least in the short term.	NR (LOW)	
De Man-Van Ginkel et al (2010) [36]	To explore the nursing role in the management of post stroke depression and to identify effective non-pharmacological interventions that nurses can use in the daily care of patients with post stroke depression	15 studies. 1 included MI (n = 411)	Patients with stroke	MI/ Care *as usual*, *placebo*,	Occurrence of depression or severity of depression	Three months after stroke MI showed significant effect on the number of depressed patients	NR (UNCLEAR)	
Hjorthoj et al (2009) [31]	To review literature on treatments of Cannabis use disorders in patients with schizophrenia spectrum disorders.	41 RCTs and Non-RCT (n = range of 7 to 694).	Schizophrenia spectrum disorders patients	Psychological interventions including MI/ *Treatment as usual (TUA)*	Reduction in substance use	Insufficient evidence exists on treating dual-diagnosis. Studies grouping several types of substances as a single outcome may overlook differential effects.	NR (UNCLEAR)	
Kelly et al (2012) [37]	To update clinicians on the latest in evidence-based treatments for substance use disorders (SUD) and non-substance use disorders among adults.	24 reviews and 43 trials (not all RCTs)	Adults with dual diagnosis, comorbidity and co-occurring disorders	MI/ Not *specified*	Substance use e.g. cannabis	MI has robust support as a highly effective psychotherapy for establishing a therapeutic alliance	NR (HIGH)	
Laker et al (2007) [32]	To examine the clinical effectiveness of HR and MI in reducing the use of harmful substances in dually diagnosed patients.	RCTs (n = 2); 11 other mixed designs	Patients with psychiatric disorders	MI /*control varied (e*.*g*. *information package*, *a self-help booklet*, *an educational treatment)*.*"*	Substance misuse e.g. Alcohol consumption	MI was effective in reducing substance misuse in short term. There may be a cost benefit in an HR approach compared with MI	NR (HIGH)	
**Domain 1: Gambling**								
Cowlishaw et al (2012) [30]	To synthesise evidence from randomised trials of psychological therapies for pathological and problem gambling.	14 RCTs including cross-over trials 4 include MI. (n = 1245 range 13–231)	Adults (mean age 44 years). 11/ 14 studies evaluated pathological gamblers	Manualised MI treatment and MI/ *No treatment’ controls*, *referral to Gamblers Anonymous and non-specific treatment component controls*.	Gambling symptom severity, financial loss from gambling; mental health outcomes	Evidence for some benefits from MI in the short-term (0–3 months) for reduced gambling behaviour, although not necessarily other symptoms of pathological and problem gambling.	M-A (LOW)	No moderate quality evidence of effectiveness (assessed with GRADE). Very low quality evidence of small effect on reducing gambling severity and financial loss at 3–12 months. MI was associated with significant reduction in gambling frequency up to a year after treatment delivery, but the long-term effects are unclear
Petry et al (2017) [63]	To review trials for psychosocial treatments of gambling problems.	21 studies. 2 trials included MI alone (n = 240)	Adults with gambling problems. Patients at medical and substance abuse clinics	MI / MI and CBT, brief education, TUA	Gambling symptom severity, financial loss from gambling e.g. DSM-IV Screen for gambling problems	2 studies that evaluated MI as a stand-alone intervention provide little evidence that MI is beneficial for reducing gambling when not combined with CB treatments.	NR (HIGH)	
Yakovenko et al (2015) [7]	To examine the effects of MI interventions compared to no treatment or interventions without MI on gambling frequency and gambling expenditure in adult disordered gamblers. A secondary objective was to assess the stability of the effects of MI over time.	8 RCTs (n = 730; range 50–165)	adult gamblers, including pathological, problem, or concerned gamblers	MI or MET/*no treatment (assessment only; control interview; CBT-based workbook; feedback session)*	Change in gambling frequency and gambling expenditure assessed post treatment	Significant short-term benefit of MI in reduction of gambling symptoms. Meta-analysis of 5 studies provided evidence of a positive effect following treatment for both outcomes.	M-A (LOW)	

**Table 3:** Characteristics of included reviews of Motivational Interviewing (MI) and summary of findings for Domain 2. Abbreviations: MI = Motivational Interviewing, BMI Brief Motivational Interviewing, RCT = randomised controlled trial, MET = Motivational Enhancement Therapy, HAART = Highly Active Antiretroviral Therapies, ETS = Environmental Tobacco Smoke, SUMSM = Substance-using men who have sex with men, T2D = Type 2 Diabetes, CVD = Cardiovascular disease, NVD = neurovascular disease, BMI = Body Mass Index, BCT = Behaviour change techniques.

**Domain 2: Musculoskeletal problems**								
**Review author**	**Objective**	**Type and Number of studies**	**Participants**	**Intervention / *Comparison***	**Outcomes**	**Authors conclusions**	**Meta-analysis (M-A) or Narrative review (NR) and overall Risk of Bias (ROBIS score)**	**Implication for clinical practice and research (Interpretation of authors of overview)**
Alperstein and Sharp (2016) [102]	To examine the efficacy of MI on the primary outcome of adherence to treatment. In addition, to investigate the efficacy of MI on the secondary outcomes of pain intensity and function	7 RCTs (n = 962)	Age 18 yrs+ with benign chronic pain (> 3 months) due to MSK problems e.g. low back pain, chronic pain, fibromyalgia and rheumatoid arthritis	MI/ *2 studies included education*, *1 placebo ultrasound*, *2 usual care*, *1 other treatment unspecified*	Primary outcome adherence to treatment for pain post treatment and at follow up; Secondary measures pain and physical function	Small to moderate effect of MI for increasing adherence to treatment for pain at short but not long term follow up. No gains in physical function.	M-A (LOW)	Low quality evidence (Assessed by GRADE) for small effects on adherence to treatment for pain. (See S3 Table) Limited evidence but promising for adherence to treatment measures.
Chilton et al (2012) [103]	To summarise the available literature and provide a detailed overview of the application and effectiveness of MI for musculoskeletal conditions.	10 studies, 3 RCTs.	2 studies of LBP, I chronic pain, 1 fibromyalgia and 1 osteoporosis.	Trans theoretical model (TTM)-based motivational counselling or MET or MI /*self-efficacy*, *workshop attendance and exercise adherence*, *pain intensity*	Self-efficacy; workshop attendance and exercise adherence; pain intensity.	The evidence base for effectiveness of MI for musculoskeletal problems is limited due to methodological factors.	NR (UNCLEAR)	
**Domain 2: Oral Health**								
Cascaes et al (2014) [64]	To analyse the effectiveness of MI at improving oral health behaviours and dental clinical outcomes	10 RCTs (n = 1989).	Subjects attending university programs or dental clinics.	MI / *traditional educational intervention" (i*.*e*. *presenting oral hygiene guidelines*, *video programs or delivering leaflets)*.	Oral health behaviours; Oral health clinical outcomes: e.g. dental caries, Dental plaque	Inconclusive effectiveness for most oral health outcomes.	NR (UNCLEAR)	Low quality evidence (assessed by GRADE) for no statistically significant difference in gingivitis measures from 3 studies (See S3 Table) Narrative reviews found inconclusive evidence due to poor methodology of the primary studies within the reviews. High quality studies required.
Gao et al (2014) [68]	To synthesize the evidence on the effectiveness of MI compared with conventional (health) education in improving oral health.	20 papers including 16 studies (n = 3252)	Dental patients, special-needs groups (adults with mental illness), disadvantaged communities	MI/ *Conventional (health) education (CE)*, *focusing on "disseminating information and giving normative advice"*	Oral Hygiene, motivation/ readiness/ confidence; knowledge of periodontal health	The potential of MI in dental health care, especially on improving periodontal health, remains controversial. Additional studies with methodologic rigor are needed for a better understanding of the roles of MI in dental practice.	NR (UNCLEAR)	
Kay et al (2016) [65]	To review the evidence regarding the use of motivational interviewing to promote positive oral health behaviours in a one-to-one setting,	8 studies 5 RCTs, 2 Quasi RCTs and 1 qualitative study	Mainly healthy adults, age up to 70 yrs. old. I trial focused on children	MI/ *TAU*, *2 mins oral hygiene*, *Traditional education and pictures of periodontal disease*	Oral hygiene, plague levels, Gingivitis, bleeding score	MI technique, which is based on the concept of autonomy support, has potential for helping patients with poor oral health	NR (LOW)	
Kopp et al (2017) [66]	To reveal the effects of MI as an adjunct to periodontal therapy.	5 RCTS (n = 481) (2 trials only provide MI without CBT)	Patients with periodontal disease	MI + Periodontal therapy / *Periodontal therapy alone*	Oral hygiene, Gingival values; plaque values, bleeding on probing; probing pocket depth.	MI as an adjunct to periodontal therapy might have a positive influence on clinical periodontal parameters and psychological factors related to oral hygiene. 3 out of 5 RCTs positive. Future studies should include fidelity measures, several MI sessions.	NR (UNCLEAR)	
Werner et al (2016) [67]	To study the effectiveness of psychological interventions in adults and adolescents with poor oral health.	11 RCTs (3 include MI n = 151)	Patients with moderate to severe chronic periodontitis. The majority of patients were >50 y of age.	MI /TAU or traditional oral health education, delivered by a dental hygienist	Dental caries, periodontitis, gingivitis, and peri-implantitis	No statistically significant difference in gingivitis when MI was compared with treatment as usual. Small but statistically significant improvement in plague. The clinical relevance of results is debatable. No statistically significant difference in oral health–related quality of life.	M-A (UNCLEAR)	
**Domain 2: Eating Disorders**								
Macdonald et al (2012) [70]	To examine the effectiveness of interventions that includes the principles and techniques of MI and its adaptations in the treatment of eating disorders.	RCT and non-controlled design (n = 783, in patient group; n = 204 carers group	Mainly female; patients and carers included	MI; MET or adapted MI / *Varied*. *7 of 13 studies included a control*	Psychological distress; Self-esteem/quality of life; Stage of change/readiness/motivation to change; Eating behaviours, attitudes and symptomatology; Carer burden	Promising results to encourage readiness to change but not conclusive	NR (UNCLEAR)	No high or moderate quality evidence to support MI for people with eating disorders. Very low quality evidence (assessed by GRADE) from 1 study in meta-analysis suggests positive results to support people with eating disorders otherwise other results are inconclusive. High quality studies required in this field to support the use of MI for eating disorders (See S3 Table).
Knowles et al (2013) [10]	To investigate whether the use of interventions specifically designed to enhance motivation in people with eating disorders is supported empirically.	5 RCTs (n = 601 ranged from 27–225)	Mean age ranged from 16.1 to 42.5 years., 97% of participants were female	MI/ TAU *or TAU+ MI or control group*	Varied outcomes and depression questionnaires	No support for widespread dissemination of MI interventions for eating disorders. The enthusiasm for the use of MI outweighs the reality of the current evidence base	NR (HIGH)	
Dray et al (2012) [69]	To reviews the empirical literature on the application of the Transtheoretical Stage of Change model and MI for the treatment of eating disorder.	9 studies, 5 RCTs.	People with Anorexia Nervosa (AN), Bulimia Nervosa (BN) and Eating Disorder	MI / *waitlist (n = 1); TAU (in-patient) (n = 2); self-help (n = 2) and CBT (n = 1)*.	Motivation, depression and self-esteem, eating attitudes, BMI and treatment dropout.	There are insufficient numbers of good quality studies and future research needs to focus on evaluating the efficacy of manual-based MI interventions	NR (HIGH)	
**Domain 2: Weight Loss Management**								
Armstrong et al (2011) [71]	To systematically review randomized controlled trials (RCTs) that investigate the effectiveness of MI for reducing body mass, measured by change in body weight or BMI in adults who are overweight or obese.	12 RCT (n = varied from 22 to 599)	Hypertension (n = 2), Diabetes (n = 3), Hyperlipidaemia (1), Firefighters, sedentary people (n = 5) and inactive adults (n = 1).	MI /*The comparison conditions* *varied from usual care*, *to print materials*, *to attention* *control*.	Change scores in body weight (kg) in standardized change scores in body mass in.	MI is moderately effective. MI associated with a greater reduction in body mass compared to controls (SMD = -0.51 [95% CI -1.04, 0.01]). Optimal dose and delivery of MI for successful weight loss have yet to be determined.	M-A (LOW)	No high or moderate quality evidence to support weight loss management. There is low quality evidence (assessed by GRADE) that MI may reduce BMI in mixed populations with moderate effects (See S3 Table) Further research of higher quality is required focusing on long-term sustainability and fidelity of treatment. As obesity is a high-profile problem worldwide, further high quality research is justified to assess the effectiveness of MI as part of a weight loss programme compared with other methods of weight control.
Barnes et al (2015) [72]	To review randomized controlled trials of MI for weight loss in primary care centres.	24 RCTs (n = 7448)	Overweight individuals with mixed diagnosis age 40s to 60; 8% men (45) to 55% men (38); 2 studies (8.3%) recruiting African–American or Hispanic/Latino participants	MI/ *Usual care*: *written discharge contract listing recommended outpatient* *medications*, *cardiac rehabilitation recommendations and health behaviour changes*, *as* *well as numerical values for ejection fraction and cholesterol*	Primary weight loss; secondary physical activity, food intake, metabolic and physiological outcomes	Potential for MI to help primary care patients lose weight. Conclusions drawn cautiously as more than half of the reviewed studies showed no significant weight loss compared with usual care and few reported MI treatment fidelity.	NR (HIGH)	
Hill et al (2013) [73]	To (1) systematically evaluate the overall effectiveness of GWG interventions derived from theories of behaviour change using a generalized health psychology perspective (2) assess the behaviour change techniques reported in the interventions.	14/21 RCTs; 7/21. 2/21 studies used MI as a BCT (n = 411 out of 3853)	Women of any pre-pregnancy BMI category in their intervention;	MI / *No information for control*	Differences in GWG, rate of GWG, or adherence to guidelines	The provision of information, motivational interviewing, self-monitoring of behaviour, and providing rewards contingent on successful behaviour may be key strategies when intervening in GWG	M-A (UNCLEAR)	
VanWormer, et al (2004) [74]	To provide a brief overview of MI and to synthesize and critically review the literature regarding its efficacy for diet modification.	RCTs (1 cluster RCT) (n = 1298)	3 x adult population (1 adolescent)	Motivational learning/ *TAU; group sessions involving dietary* *and behavioural skill training*	Blood pressure Weight Sodium intake Alcohol intake Dietary intake (Attendance at group session. Self-monitoring of fat intake	MI used in combination with nutrition education is at least moderately efficacious for facilitating diet modification, offering an advantage beyond standard education alone	M-A (HIGH)	
**Domain 2: Management of Metabolic Disorders (Diabetes)**								
Clifford Mulimba, and Byron-Daniel(2014) [75]	To systematically examined the evidence of MI in improving health behaviours in adults with diabetes. In particular lifestyle and non- pharmacological self-management techniques.	8 studies, 6 RCTs (n = 1721)	Adults with type 1 and 2 diabetes. diagnosed adults, mixed sex. Age range 16–80	MI / *Varied including usual care*, *attention placebo*, *diabetes education and treatment recommended for achieving glycaemic* control.	Physical activity, smoking, blood-glucose control, diet and weight management, managing cholesterol, blood pressure, alcohol consumption.	Only four of the studies found positive and significant effects of MI on diabetes self-management outcomes in four of the eight health behaviour topics investigated. These behaviours were smoking, blood-glucose control, diet and weight management.	NR (UNCLEAR)	Very low quality evidence (Assessed by GRADE) for no statistically significant difference for standardised measurement used in diabetes treatment (See S3 Table). Narrative reviews are inconclusive. High quality research is needed to investigate the effects of MI on diabetes management.
Ekong and Kavookjian (2016) [76]	To examine empirical evidence for the impact of MI on behaviour change and resultant clinical outcomes in adults with T2D.	14 RCTs	Adults > 18 years with T2D.	MI based intervention/ *usual care or a non-MI intervention*.	Dietary changes, physical activity, smoking cessation, and alcohol reduction		NR (UNCLEAR)	
Jones et al (2014) [77]	To review the evidence for the efficacy of MI in promoting glycemic control in people with diabetes by examining the mean magnitude of effect in change in levels of glycated haemoglobin (HbA1c) as a function of MI.	13 RCTs (n = 1223 type 1; n = 1895 type 2)	Adults with Type 1 (n = 4); Type 2 (n = 7); Type 1 and 2 (n = 1); NR (n = 1).	MI/ *UC (diet counselling; support visits; diabetes education session & support club; videophone healthy lifestyle sessions; meetings at diabetes clinic; varied visits; structured diabetes education sessions)*	Measure of HbA1c. HbA1c is a standardised measurement used in diabetes treatment and a direct indicator of diabetes management	MI in the management of blood glucose levels appears to be limited. Change in glycemic control in people who received a MI compared to a control group was not statistically significant. MI aimed at helping people manage their diabetes may need to be re-examined.	M-A (HIGH)	
Lin et al (2014) [78]	To evaluate the literature on the effectiveness of lifestyle modification programs (LMPs) on the metabolic risks in adults with metabolic syndrome (MetS); To determine whether the LMPs are effective in improving patient-reported outcomes (PROs).	5 RCTs (n = 256 (In MI RCT Fitch et al 2006 N = 30)	Adults over 18 years old diagnosed with MetS based on NCEP-ATP III or IDF.	MI/ *Usual care*	Waist circumference, HDL, triglyceride, BP, and FBG. The PROs related to quality of life; other psychological health factors	LMPs exhibited positive effects on some metabolic risks and on quality of life in adults with Mets	NR (UNCLEAR)	
Soderlund (2018) [79]	To examine the effectiveness of MI for physical activity self-management for adults diagnosed with diabetes mellitus type 2 (TD2).	9 studies (RCTs, quasi studies and pilot studies n = >3260)	Adults with T2D. Mean age 50–60 years old	MI/ *Usual care*	PA accelerometer, blood glucose monitor Self-report: medication usage, self-care outcomes	MI sessions should target a minimal number of self-management behaviours, be delivered by counsellors proficient in MI, and use MI protocols with an emphasis placed on duration or frequency of sessions.	NR (HIGH)	
Thepwongsa et al (2016) [80]	To evaluate the effects of MI delivered by GPs to Type 2 diabetes patients on the change of GPs’ attitudes, knowledge and practices and patients’ clinical outcomes.	8 studies. 5 RCTs (n = 181 GPs and n = T2D patients)	GPs and Adults with T2D.	MI / *unclear but 1 study had no control*	GP satisfaction, knowledge, behavioural changes, process of care and clinical outcomes e.g. blood sample tests	Few studies have examined evidence for the effectiveness of MI delivered by GPs to T2D patients. Evidence to support the effectiveness of MI on GP and patient outcomes is weak.	NR (UNCLEAR)	
**Domain 2: Management of Neurovascular (Stroke) and Cardiovascular disease (CVD)**								
Cheng et al (2015) [81]	To investigate the effect of MI for improving activities of daily living after stroke.	1 RCT (n = 411)	18 years and over	MI/ *participants received usual stroke care*, *including inpatient care and discharge planning through regular multidisciplinary team meetings"*	Primary measure—Barthel Index, Functional Independence Measure, Modified Rankin Scale, Katz Index of Activities of Daily Living, Rehabilitation Activities Profile. Secondary outcomes Changes of mood, e.g. GHQ-28)	Insufficient evidence to support the use of MI for improving activities of daily living after stroke. Limited evidence that participants receiving MI were more likely to have a normal mood than those who received usual care at 3 months and 12-months follow-up.	M-A (LOW)	There is insufficient evidence to make conclusions about the impact of MI on outcomes of neurovascular disease and CVD.
Hildebrand (2015) [82]	To evaluate the effectiveness of occupational therapy interventions to prevent or mitigate the effects of psychological or emotional impairments after stroke.	39 RCTs (only 1 MI) (n = 240 men, 171 women)	Stroke patients mean age70 years;	MI/ *usual care medical*, *nursing and therapy care in-patient setting*	Health measures. MI trial included GHQ and Yale Depression questionnaire	MI was found to be effective in improving depression and mental HRQOL.	NR (HIGH)	
Lee et al (2016) [83]	To systematically review the effectiveness of MI on lifestyle modification and physiological and psychological outcomes for clients at risk and diagnosed with CVDs	9 RCTs (n = 4684)	Participants at risk of developing CVDs or with diagnosed CVDs, age 16 to 89. 7yrs	MI and MET/ Seven *studies included usual care provided*, *whereas two studies did not clearly mention the components"*	Lifestyle modification, cessation or reduction in smoking, physical activity levels, intake of fruits and vegetables and dietary fat. Physiological and psychological outcomes. e.g. BP	Insufficient evidence to be confident about conclusions. MI positively, improved client’s systolic and diastolic blood pressures but not significantly. MI might have favourable effect on improving clients’ depression. No effect of MI for other outcomes.	NR (LOW)	
**Domain 2: Management of Sexual Health Behaviour**								
Berg et al (2011) [84]	Review of the effectiveness of behavioural interventions adapting the principles and techniques of MI on HIV risk behaviours for men who have sex with men (MSM).	Mainly RCTs and quasi-RCTs (n = 6051)	Gay, homosexual, or bisexual men	MI or MET / "*no intervention*, *waiting list control*, *placebo psychotherapy or other active therapy*, *or pharmacotherapy*.*"*	iSTI/HIV acquisition; unprotected sex, AOD use; (STI/HIV testing. Enhanced motivation for change concerning sexual risk	The effectiveness of MI as an intervention strategy for unsafe sexual and substance use behaviours among MSM is uncertain. It was largely equivalent to other active and minimal treatments for HIV-related behaviours.	M-A (LOW)	No high or moderate quality evidence for of the effectiveness of MI on sexual behaviour. (Assessed by GRADE). There is moderate quality evidence of no benefit or harm on some outcome related to sexual health behaviour (See S3 Table). There is low quality evidence for small effects for men who have sex with men on some outcomes relating to unprotected anal sex but the evidence is inconclusive. (See S3 Table) Very low quality evidence of effect of contraceptive use in the short term for some women at risk of pregnancy (See S3 Table). Further high quality research required.
Carrico et al (2016) [85]	To examine RCTs testing the efficacy of behavioural interventions to reduce CAI and substance use among SUMSM.	12 RCTs 2 MI only (n = 293) MI + TTM (n = 235)	Substance-using men who have sex with men (SUMSM)	MI / *Education*	Level of unprotected sex; level of substance and alcohol use	Further research is needed to examine if integrative approaches that cultivate resilience and target co-occurring conditions demonstrate greater efficacy	NR (HIGH)	
Dillard et al (2017) [86]	To examine the use of MI to improve health outcomes in persons living with HIV (PLWH).	19 studies (14 adults)	Male or Female diagnosed with AIDS or HIV.	MI/ *Advice or education*, *health promotion programme*, *video information*, *Standard care or TAU*	Behavioural or health outcomes. e.g. adherence, viral load, and CD4+ T-cell counts.	MI can be an effective method of therapeutic communication for PLWH, who struggle with adherence, depression, and risky sexual behaviours.	NR (UNCLEAR)	
Naar-King, et al (2012) [88]	To identify the efficacy of MI in relation to sexual risk and substance use.	12 studies, 6 RCTS (n = ranged from 40–490)	Male or female	MI/ *1x assessment only education only (same dose as MI group); single session video; 4 sessions MI vs 1 session MI; referral only; hand-outs*	Level of unprotected sex; level of drug use; level of substance abuse, level of alcohol	MI has the potential to reduce sexual risk behaviour, but the effects on reducing substance use were less consistent	NR (HIGH)	
Wilson et al (2015) [87]	To review evidence on the impact of MI on effective contraceptive use in women of childbearing age	8 RCTs (n = 3424 (6 RCTs include adults)	Women of reproductive age at high risk of pregnancy. use.	MI/ *Usual care or standard practice*	Contraceptive use Unwanted pregnancy at 12 and 24 months	MI significantly increased effective contraceptive use immediately after and up to four months post-intervention. The effect without reinforcement is short lasting. No difference in subsequent pregnancies or births at the two-year period.	M-A (UNCLEAR)	
**Domain 2—Engagement with Interventions**								
Karmali Kunal et al (2014) [98]	To determine the effects, both harm and benefits, of interventions to increase patient uptake of, or adherence to, cardiac rehabilitation	18 RCTs (1 MI n = 252)	Adults over 18 with mixed coronary heart disease eligible for cardiac rehabilitation. Mean age ranged from 51 to 66	MI including motivational leaflets/ *Usual care*	Measures of uptake of or adherence to cardiac rehabilitation and its exercise, education and lifestyle components	Weak evidence to suggest that interventions to increase the uptake of cardiac rehabilitation are effective but only 1 trial included MI.	NR (LOW)	Low quality evidence (Assessed by GRADE) for statistically significant difference in engagement with interventions for adults with mental health issues. (See S3 Table)
Lawrence et al (2017) [100]	To examine the published research on MI as a pre-treatment to enhance attendance among individual’s treatment-seeking and non-treatment-seeking for mental health issues	14 RCTs (12 included in Meta-analysis; n = 803)	Patients diagnosed with a mental illness according to validated diagnostic tools	MI as a pre-treatment/ any *alternative intervention which did not contain elements* *MI; or TAU or no* *treatment*	Post-MI treatment attendance	MI pre-treatment improved attendance relative to comparison groups. Individuals not seeking treatment for mental health issues benefited the most from MI.	M-A (LOW)	
Miller et al (2017) [99]	To examine the efficacy of MI for improving health screening uptake.	14 studies 11 RCTs (n = 6059)	Patients referred for cancer screening uptake (N = 8); HIV testing (N = 3); attendance of a hepatitis C screening appointment and sexually transmitted infections.	MI and BMI/ Group *Mailed cover letter*, *generic pamphlet about CRC screening*, *TAU*	Health screening attendance e.g. mammogram, HIV, colonoscopy, sigmoidoscopy, or faecal occult blood testing	MI shows promise for improving health screening uptake. Variability amongst the studies, limited number of RCTs makes it difficult to draw conclusions on impact of MI on health screening uptake.	NR (UNCLEAR)	
**Domain 2 –Adherence to Medication Interventions**								
**Review author**	**Objective**	**Type and Number of studies**	**Participants**	**Intervention / Comparison**	**Outcomes**	**Authors conclusions**	**Meta-analysis (M-A) or Narrative review (NR) and overall Risk of Bias (ROBIS score)**	**Implication for clinical practice and research (Interpretation of authors of overview)**
Al- Ganmi et al (2016) [89]	To critically appraise and synthesize the best available evidence on the effectiveness of interventions suitable for delivery by nurses, designed to enhance cardiac patients’ adherence to their prescribed medications.	14 RCTs (3 include MI (n = 533)	≥18 years old with a diagnosis of a cardiac disease	Behavioural intervention strategy suitable for delivery by nurses and had either a primary or secondary aim to increase the adherence to medication	Adherence to cardiac medication	Substantial heterogeneity limited robustness of conclusions, MI appeared promising as means to enhance cardiac medication adherence.	NR (LOW)	No high or moderate quality evidence for adherence to medication interventions or engagement with interventions. Low and very low quality evidence (Assessed by GRADE) for small effects on medication adherence e.g. adults with chronic diseases (See S3 Table) The most promising results are for adherence to HAART medication in people who are HIV-positive, but the higher quality reviews concluded that the methodology within the trials was poor. Further higher quality research required.
Binford and Altice(2012) [90]	To systematically review the literature on interventions to improve combination antiretroviral therapy (cART) adherence and virologic outcomes among HIV-infected persons who use drugs	One RCT and 2 pilot trials using MI/CBT 1 RCT (n = 65)	HIV infected people who use drugs	MI/ *time and content equivalent without MI in 1 RCT*.	adherence to drug talking and Biological and Immunological impact	Good short-term gains in cART adherence but limited efficacy in sustaining adherence improvement and viral load reduction at follow-up points	NR (UNCLEAR)	
Easthall et al. (2013) [91]	To describe and evaluate the use of cognitive-based behaviour change techniques as interventions to improve medication adherence.	26 RCTs (n = 5216)	A range of conditions including asthma, diabetes and hypertension. HIV infected people	BCTs including MI/ *TAU; for 13 studies (50%)*, *standard care involved some form of technique to improve adherence*	Medication adherence (the definition for this differed across trials).	Cognitive-based behaviour change techniques are effective interventions eliciting improvements in medication adherence. Nonspecific for MI	M-A (LOW)	
Hill et al (2012) [96]	To systematically examine the MI intervention literature and report evidence and gaps regarding outcomes of MI as an intervention to improve HAART adherence in patients infected with HIV.	5 RCTs (n = 963 ranged from 141 to 326 patients)	Patients with HIV. Mean age of 38 and 43.6 years old.	MI/ *standard care/normal care; Medical consultation; eight* *session educational* *programme; 1 educational audiotape*	Adherence to treatment (HAART) all studies measured viral load.	MI is a promising intervention to improve HAART adherence in HIV-positive individuals, but further studies of rigorous methodological quality are needed to fully understand the effect of this intervention	NR (UNCLEAR)	
Hu et al (2014) [94]	“To provide a systematic review of interventions to increase medication adherence in racial and ethnic minority populations”.	36 RCTs and Quasi RCTs (n = 658 for 7 trials of MI only)	African-American population. Patients’ with chronic conditions, HIV/AIDS, hypertension, asthma.	MI/ *Unclear*	Adherence to medication	MI appeared to be an effective intervention for some African-American populations. Studies conducted with HIV positive patients, patients with asthma and hypertension found MI improved adherence.	NR (HIGH)	
Nieuwlaat et al (2014) [92]	To assess the effects of interventions intended to enhance patient adherence to prescribed medications for medical conditions, on both medication adherence and clinical outcomes.	182 in total (n = 46,96) 13 studies included MI	Patients prescribed medication for medical disorder, not for addictions.	MI alone and in combination with CBT/ *varied*: *e*.*g*. *usual care*, *Treatment as usual*, *GP advice with no training in MI*	Adherence and clinical outcomes	Effects were inconsistent from study to study, and only a minority of lowest risk of bias RCTs improved both adherence and clinical outcomes.	NR (LOW)	
Palacio et al (2016) [95]	To evaluate the impact of MI and of the MI delivery format, fidelity assessment, fidelity-based feedback, counsellors’ background and MI exposure time on adherence.	17 RCTs (n = 2529)	Patients with HIV, Asthma, Osteoporosis, CVD and RA prescribed medication e.g. (HAART). 12 focused on Minorities.	MI /*TAU*, *other counselling*, *health education session*, *Pharmacotherapy*	Medication adherence	MI improves medication adherence at different exposure times and counsellors’ educational level. Results inconsistent.	M-A (UNCLEAR)	
Rueda el al (2006) [97]	To conduct a systematic review of the research literature on the effectiveness of patient support strategies and education for improving adherence to highly active antiretroviral therapy (HAART) in people living with HIV/AIDS.	19 RCTs (n = 2159)	General HIV-positive populations, women, Latinos, or adults with a history of alcohol dependence	MI/ *control arm received usual or standard adherence support or an alternate intervention*	Adherence to HAART at least 6 weeks after study initiation. electronic monitoring, pill counts, medication diaries, patient self-report, provider report, clinic and pharmacy records.	Interventions targeting practical medication management skills, interventions administered to individuals’ vs groups, and those interventions delivered over 12 weeks or more were associated with improved adherence outcomes.	NR (LOW)	
Zomahoun et al (2017) [93]	To assess whether MI interventions are effective to enhance medication adherence in adults with chronic diseases and to explore the effect of individual MI intervention characteristics.	19 RCTs with 17 included in meta-analysis. 6 RCTs MI only 11/16 compared MI with TAU (n = 4221)	Patients with epilepsy, kidney disease, diabetes, HIV/AIDS, hypertension, schizophrenia, osteoporosis and psychotic disorder	MI / *Control TAU*, *Education video*, *psychiatric interview*, *self-monitoring condition*	Medication adherence and health-related behaviour	MI interventions might be effective at enhancing medication adherence in adults treated for chronic diseases. Interventions based on MI only were more effective than those based on MI plus other interventions.	M-A (LOW)	
**Domain 2: Cancer care**								
Spencer and Wheeler (2016) [101]	To explore the use of (MI) interventions among cancer patients and survivors	14 studies; 8 RCTs 6 cohort studies (n = 1554)	Cancer patients or survivors. Most common Breast cancer.	MI / *TAU or leaflet*	Smoking cessation; body weight; physical activity; psychological measures; fatigue; self-care; pain; cancer related stress.	Solid evidence exists for the efficacy of MI to address lifestyle behaviors as well as the psychosocial needs of cancer patients and survivors.	NR (HIGH)	Limited available evidence from small sample size.
**Domain 2: Management of patients with irritable bowel disorder (IBD)**								
Wagonera, & Kavookjianb (2017) [104]	To determine: 1) the extent to which MI impacts outcomes for those diagnosed with IBD, and 2) optimal MI methods used to achieve desired outcomes	4 studies (n = 45 to 278 total 460)	Patients with IBS ulcerative colitis. age from 20 to 82 years-old	MI / unclear	Adherence, patient satisfaction with provider, quality of life, and patient-perceived provider empathy.	MI can be effective in improving outcomes for individuals with IBD e.g. improved adherence rates, greater advice-seeking behavior, and perceived providers as having more empathy.	NR (UNCLEAR)	Limited evidence from very small sample size difficult to draw conclusions.

**Table 4:** Characteristics of included reviews of Motivational Interviewing (MI) and summary of findings for Domains 3 and 4. Abbreviations: MI = Motivational Interviewing, BMI Brief Motivational Interviewing, RCT = randomised controlled trial, MET = Motivational Enhancement Therapy, HAART = Highly Active Antiretroviral Therapies, ETS = Environmental Tobacco Smoke, SUMSM = Substance-using men who have sex with men, T2D = Type 2 Diabetes, CVD = Cardiovascular disease, NVD = neurovascular disease, BMI = Body Mass Index, BCT = Behaviour change techniques.

**Domain 3: Reviews focused on multiple health related problems and /or multiple health behaviour**								
**Review author**	**Objective**	**Type and Number of studies**	**Participants**	**Intervention / Comparison**	**Outcomes**	**Summary of authors results**	**Meta-analysis (M-A) or Narrative review (NR) and overall Risk of Bias (ROBIS score)**	**Implication for clinical practice and research (Interpretation of authors of overview)**
Burke et al (2003) [106]	To review individually delivered interventions that incorporated the four basic principles of MI.	30 trials (n = 6275). ranged from 22 to 952, mean of 206	Multiple groups of people from different settings	Adapted MI (AMI) / *varied*: *AMI + relapse prevention (RP); RP alone; CBT; No treatment; placebo control; education booklet; brief feedback*	Drinking frequency); BAC (peak) blood alcohol concentration exercise adherence and HIV risk behaviour	Only 11 /30 studies produced statistically significant effect of MI. AMIs were equivalent to other active treatments and superior to no-treatment or placebo controls for problems involving alcohol, drugs, and diet and exercise.	M-A (UNCLEAR)	No high quality evidence. For all behaviours combined there is Low quality evidence of small effects of MI judged against a “weak” comparison but no benefit over a “strong” comparison Moderate quality evidence (assessed by GRADE) that MI increases physical activity participation in some populations, but the data is limited by small trials (See Tables 5 & 6) High quality trials are required and justified due to the large number of people who remain inactive. Focus should be on intervention fidelity. As the narrative reviews in this section are judged as high chance of bias, no further conclusion can be drawn with confidence. More research is needed to assess fidelity of technology assisted MI
Dunn et al (2001) [107]	To examine the effectiveness of brief behavioural interventions adapting the principles and techniques of MI to four behavioural domains	29 RCTs (n = 6330 ranged from 23–1726)	Mixed male /female, with health problems; substance abuse, smoking, HIV risk and diet/exercise problems	MI /*no treatment or a comparison treatment*	Binge drinking, exercise participation, drug usage, cigarette usage,	Only modest evidence that MI works at least as well as other treatments for clients with low baseline readiness. The evidence is inconclusive	NR (UNCLEAR)	
Hettema et al (2005) [8]	To assess the effectiveness of MI across multiple behavioural problems	72 RCTs and controlled studies. (n = 14,26)	16/37 (43%) were predominantly or entirely African American	MI/ *no treatment or placebo; MI added to standard or specified treatment; standard or specified treatment*	alcohol use, treatment compliance	Large variation in effect size across studies. No relationship between outcomes and methodological quality or other outcomes e.g. time of follow-up assessment, comparison group type or provider. Manualised interventions yielded weaker effect.	M-A (UNCLEAR)	
Lundahl et al (2010) [108]	To investigate the unique contribution MI has on counselling outcomes and how MI compares with other interventions.	119 studies (some RCTS) (n = 9618)	Majority sample were white, African American Or Hispanic. Other groups not recorded	MI/ *Waiting list/control groups; TAU with a defined or specifically named program; written materials; an attention control group*.	Multiple outcomes	Judged against weak comparison groups, MI produced statistically significant small effects. Judged against specific treatments, MI produced nonsignificant results	M-A (UNCLEAR)	
Martins et al (2009) [109]	To critically review the research in three emerging areas in which (MI) is being applied: diet and exercise, diabetes, and oral health.	37 empirical studies; 24 exercise and diet; 9 diabetes; 4 oral health (n = 15012)	Adult obese women, southern Asian women; adults with diabetes, smokers physically inactive adults,	MI / *behaviour therapy*	Varied weight loss, fat intake, oral health, exercise uptake.	MI effective in supporting health behaviour change for 3 health behaviour domains, Oral health, diabetes and diet and exercise.	M-A (HIGH)	
O'Halloran et al (2014)	To determine if MI leads to increased physical activity, cardiorespiratory fitness or functional exercise capacity in people with chronic health conditions.	10 RCT or controlled trial (n = 981)	People 18 or over with a chronic health condition.	MI / *Supervised exercise x 1; behavioural weight loss x1; WLC x2*, *Standard written information/education x 2; usual care x 2*	Physical activity levels; cardiorespiratory Fitness; functional exercise capacity	Moderate quality evidence that MI may have a small positive effect on self-reported physical activity in people with chronic health conditions.	M-A (LOW)	
Rubak et al (2005) [9]	To evaluate the effectiveness of MI as an intervention tool and to identify factors shaping outcomes in the areas reviewed.	72 RCTs (19 meta-analysis) (n = 4173)	Mainly adults (older adolescents also included)	MI/ *Traditional* *advice giving’ e*.*g*. *patients’ problem is viewed from a biomedical perspective*.	Health outcome; e.g. blood glucose, blood cholesterol; BMI, smoking cigs/day, blood alcohol, BP; utilisation of healthcare services; length of hospital stay, subjective reports.	MI outperforms traditional advice giving in the treatment of a broad range of behavioural problems and diseases. A prolonged follow-up period increased the percentage of studies showing an effect.	M-A (UNCLEAR)	
Shingleton et al (2017) [105]	To describe and evaluate the methods and efficacy of technology-delivered MI interventions (TAMIs).	41 studies most RCTs (34 adults’ population n = approx. 11000)	Mainly adults with substance abuse problems; other health or social problem e.g. weight gain, addiction, criminals,	Technology-delivered MI interventions (TAMI) (some combined with other therapy) / various TAU e.g. Follow-up with school nurse	Acceptability/ feedback regarding the intervention and/or behavioural or psychological change related to the target health behaviour	Limited data regarding efficacy. Strategies to deliver relational components remain a challenge. Future research should incorporate fidelity measures. TAMIs are feasible to implement and well accepted.	NR (HIGH)	
Thompson (2011) [110]	To review MI and to inform education, research and practice in relation to cardiovascular health.	9 studies, 3 including MI (n = 546 (MI = 266)	Adults with at least one or more newly diagnosed or existing cardiovascular risk factors	MI/ *TAU*	Obesity, Smoking, treatment non-compliance, physical inactivity medical outcomes e.g. BP.	MI is an effective approach to changing behaviour. It offers promise in improving cardiovascular health status.	NR (HIGH)	
**Domain 4: Reviews Focused on Behaviour Change Interventions in Specific Settings**								
**Review author**	**Objective**	**Type and Number of studies**	**Participants**	**Intervention / Comparison**	**Outcomes**	**Summary of authors results**	**Meta-analysis (M-A) or Narrative review (NR) and overall Risk of Bias (ROBIS score)**	**Implication for clinical practice and research (Interpretation of authors of overview)**
Kohler et al (2015) [112]	To examine changes in alcohol consumption after brief MI for young people with existing alcohol use problems, who were admitted to an **emergency care unit** alcohol positive, with an alcohol-related trauma, or with a history of elevated alcohol consumption	6 RCTs (2 specifically over 18) n = 1433 age 18–25)	Young people in emergency care who screened positively for past or present risky alcohol consumption.	BMI/ *standard care*, *including written information (e*.*g*. *alcohol-use risk handout*, *educational brochure*.	Alcohol consumption, frequency and quantity	MI was never less efficacious than a control intervention. Two trials found significantly more reduction in one or more measures of alcohol consumption in the MI intervention group.	M-A (UNCLEAR)	Narrative reviews support the meta-analyses suggesting there is no difference in outcome between professional groups who deliver MI. High quality research assessing competency and fidelity of MI interventions is needed to confirm if any benefits reported by Merz et al (2015) are sustained over 12 months.
Knight et al (2006) [118]	To identify the extent to which MI has been used in different physical **health settings** and appraise the effectiveness of MI	4 RCTs, 1 non-random controlled trial and 3 pilot studies.	Hypertension, diabetes, asthma, hyperlipidaemia and heart disease.	MI/ *TAU (usual care)*	Psychological, physiological and life-style change outcomes	MI has high face validity across several domains in physical health care settings. Recommendations for its dissemination in this area cannot yet be made.	NR (UNCLEAR)	
Lundahl, et al (2013) [6]	To investigate MI’s efficacy in **medical care settings**	48 RCTs (n = 9618)	Reported as moderator analyses rather than general participant description	MI in medical setting/ *7 studies used a traditional waiting list group*, *(2) 16 studies used information only groups*, *28 studies employed ‘‘treatment-as-usual”*	Prognostic markers, disease endpoints, risk reduction behaviours; physical functioning and quality of life, substance abuse, patient adherence to medical advice and patient approach to change.	The emerging evidence for MI in medical care settings suggests it provides a moderate advantage over comparison interventions and could be used for a wide range of behavioural issues in health care.	M-A (UNCLEAR)	
Merz et al (2015) [113]	To identify evidence to reduce alcohol use and prevent alcohol related consequences in young adults (18–24 years old) admitted to the **emergency department** following acute alcohol intoxication.	4 RCTs (n = 618)	Young adults (18–24).	Brief MI/ *usual care (2 trials); 1 x personalised feedback + phone booster at 1 & 3 months; 1 x education brochure + 5 min discussion*	Various alcohol-related outcomes: change in alcohol use, alcohol-related problems/risks, drinking & driving	Inconclusive evidence. Most effective interventions include at least one therapeutic contact several days after the event. Successful interventions included booster sessions. Benefits were sustained over 12 months.	NR (LOW)	
Noordman et al(2012) [114]	To review effectiveness of face-to-face communication-related BCTs provided in **primary care** and to explore which health care provider is more effective in using face-to-face communication-related BCTs?	50 RCTs. 9 include MI	18+ years. People with risky lifestyle behaviour. Patients with heart or vascular disease	BCTs including MI/ *advice*, *pamphlets (or booklets) unstructured information*, *minimal care "usual care" to no intervention*.	Subjective (self-reported) and objective outcome measures related to patients’ lifestyle behaviour.	MI, education and advice can be used as effective communication-related BCTs delivered by physicians and nurses.	NR (LOW)	
Purath, et al (2014) [117]	To review MI interventions used to elicit health-related behaviour change among older adults in **primary care settings**.	8 RCTs and Pilot RCTs (n = 1388)	Older people. Average participant age was over 60 years	MI / *varied 1 x newsletter; 4 x usual care; 1 x tailored information; 1 x telephone information call*	Weight loss, participation in physical activity; smoking cessation; fruit and vegetable consumption	MI may be effective when incorporated into health promotion and disease prevention interventions.	NR (UNCLEAR)	
Taggart et al (2012) [115]	To evaluate the effectiveness of interventions used **in primary care** to improve health literacy for change in smoking, nutrition, alcohol, physical activity and weight.	52 studies	Adults aged 18 years and over. Mixed sex, different socioeconomic backgrounds	MI/ *no description*	Health literacy outcomes; Knowledge Skills; Self efficacy	Individual MI counselling and written materials were more effective in achieving impacts around smoking cessation compared to group education.	NR (LOW)	
VanBuskirk et al (2014) [116]	Is MI effective in improving behaviour modification in patients seeking treatment for health conditions in **primary care settings**?	12 RCTs varied from 26–515 (n = 3326)	Primary care patients; mixed race and sex.	*MI /* no treatment; mailed pamphlet; usual care; usual care + pamphlet; anti-smoking advice	Substance use outcomes; bodyweight reduction; physical activity, adherence.	MI is useful in clinical settings. 1 MI session may be effective in increasing change-related behaviour on certain outcomes.	M-A (UNCLEAR)	

### Results of meta-analyses

Thirty-nine reviews reported meta-analyses but it was not possible to extract data from all. [6–9, 12, 21–23, 26, 27, 30, 38, 39, 41, 45, 46, 48, 49, 56–58, 67, 71, 73, 74, 77, 81, 84, 87, 91, 93, 95, 100, 102, 106, 108, 111, 112, 116]. Table 5 provides a brief summary of results from the reviews with pooled data comparisons.

**Table 5:** Summary of reviews contributing data to comparison that provide moderate, low and very low quality evidence of effects of Motivational Interviewing (MI).

Sub-groups	Reviews contributing data to overview	Reviews with data, but superseded by more up-to-date or higher quality review judged by overview authors using ROBIS	Reviews in which there was no data suitable for extraction	Moderate quality evidence relating to effect of MI	Low or very low quality evidence relating to effect of MI
**Domain 1- Interventions aimed at stopping / preventing behaviour**					
Smoking cessation	Lindson-Hawley et al 2015 [12] (update of Lai et al 2010) [119] Rabe et al 2013 (subgroup) [26] Hettema et al 2010 [23] (pregnancy subgroup)	Burke et al 2003 [106] Hettema et al 2005 [8] Rubak et al 2005 [9] Lundahl et al 2010[108] Heckman et al 2010[22]	Ebbert et al (2015) Smokeless tobacco [21] Stead et al (2006) [27]	Small effect on smoking cessation compared with usual care or brief advice at 6–12 months follow-up	Small effect on smoking cessation in pregnant women and, in emergency departments
Substance abuse (Alcohol)	Foxcroft et al 2014 [49] Vasilaki et al 2006 [58]	Burke et al 2003 [106] Hettema et al 2005 [8] Rubak et al 2005 [9] Lundahl et al 2010 [108]	Tanner-Smith (2015) [39]	Moderate effect on alcohol consumption. Small effect on binge drinking, frequency and quantity of drinking in mixed populations (including young people < 25) mainly in short term <4 months. Evidence of no benefit or harm for drunk driving and risky behaviour relating to alcohol or binge drinking in the long term >4 months	Small effects for short term reduction in drunk driving, average blood alcohol concentration (BAC), and alcohol related problems < 4months
Substance abuse (Drugs)	Darker 2015 [48] Lundahl et al 2010 [108] Smedslund et al 2011 [56] Terplan et al (2015) [38]	Burke et al 2003 [106] Terplan (2007) [57]	Carey 2012 [45] (computer delivered alcohol interventions)		Currently there is insufficient evidence to support the use of MI to reduce Benzodiazepines use. Small effects on readiness to change and extent of substance abuse. Little evidence that psychosocial interventions reduce continued illicit drug use in pregnant women enrolled in drug treatment.
Substance abuse (Drugs) Marijuana	Lundahl et al 2010 [108] Lundahl et al 2013 (medical care settings) [6] Gates et al 2016 [41]				Small effects on abstinence and number of drugs taken in people attending general medical care settings. No intervention was consistently effective at nine-month follow-up or later.
Substance abuse (drugs or alcohol)	Smedslund et al 2011 [56]	Burke et al 2003[106]		Small effects on drug /alcohol in mixed population e.g. college drinkers, outpatient alcohol clinics, and drink drivers at < 6 month when compared with no treatment. Evidence of no benefit or harm compared with other active treatment or treatment as usual	
Gambling	Cowlishaw et al 2012 [30] Yakovenko 2015 [7]	Lundahl et al 2010 [108]			Very low quality evidence of small effect on reducing gambling and financial loss at 3–12 months Significant short-term benefit of MI in reduction of gambling symptoms.
Risk Behaviour (HIV risk)	Hettema et al 2005 [8]	Burke et al 2003[106]			Small effects on risk behaviour for HIV
**Domain 2- Interventions aimed at promoting specific health behaviour**					
Physical activity promotion	O’Halloran et al 2014 [111]			Small effect on self-reported physical activity in people with some, but not all, chronic health conditions immediately post intervention	Very low quality evidence of very small effect on cardiorespiratory fitness immediately post interventions
Weight loss management	Armstrong et al 2011 [71]	Burke et al 2003 [106] Hettema et al2005 [8] Rubak et al 2005 [9] Lundahl et al 2010 [108]			Greater reduction in body mass and BMI compared with controls
Management of metabolic disorders	Jones et la 2014[77]				MI in the management of blood glucose levels is limited. Effects not statistically significant. MI aimed at helping people manage their diabetes may need to be re-examined.
Management of neurovascular disorders	Cheng et al 2015 [81]				Insufficient evidence to support the use of Motivational Interviewing for improving activities of daily living after stroke (1 study only).
Engagement with interventions and adherence to medication	Hettema et al 2005 [8] Lundahl et al 2013 (medical care settings)[6], Palacio et al (2016) [95], Lawrence et al (2017) [100] Zomahoun et al (2017) [93]		Easthall et al(2013) [91]		Low quality evidence of small effects on medication adherence and treatment compliance e.g. breast feeding, self-care, reducing sedentary behaviour. Attendance with treatment for people with mental health issues
Management of Musculoskeletal problems	Alperstein and Sharp (2016) [102]				Low quality evidence of small effects on, adherence to treatment for pain management and reduction in pain
Eating disorders	Lundahl et al2010 [108]	Hettema et al 2005 [8]			Very low quality evidence (1 study) to support eating disorders
Parenting practice	Lundahl et al 2010 [108]				Small effect on health related behaviour (2 studies only)
Drinking safe water	Lundahl et al 2010 [108]	Hettema et al 2005 [8]			Very low quality evidence (1 study) Small effects on behaviour relating to drinking safe water
Sexual health	Berg et al 2011 [84] (HIV risk promotion for men who have sex with men Wilson et al 2015[87]	Hettema et al (2005) [8]		Evidence of no effect or benefit on behaviour related to sexual health in men who have sex with men with HIV	Small effect on men who have sex with men on condom use, alcohol use, and reducing unprotected anal sex. Small effect on contraceptive use in women at 1–12 months follow up. Moderate effect on HIV knowledge and behaviour. Some short-term evidence for increasing effective contraceptive use immediately after and up to 4 months post-intervention. No difference in subsequent pregnancies or births at the two-year period.
Oral Health	Werner et al (2016) [67]				Evidence of no statistically significant effect on Gingivitis measures.
**Domain 3 &4 –Reviews focused on behaviour change interventions for multiple health related problems and/or multiple behaviour problems in specific settings**					
*ALL BEHAVIOURS COMBINED*	Lundahl et al 2010 [108] Lundahl et al 2013 (medical care settings) [6] Van Buskirk [116] (primary care settings)				Small statistically significant effect when all behaviours combined for different populations and settings judged against a weak comparison group e.g. usual care or no treatment. No difference between groups when judged against other interventions. Small effect of MI when all behaviours combined in general medical care and primary care settings.

Of the 155 meta-analysis comparisons that were extracted, we found no high quality evidence. Twenty seven comparisons provide moderate quality evidence according to the GRADE criteria. Most of this evidence was categorised in Domain 1 (Stopping an unhealthy behaviour). Further details of the outcomes for the moderate quality evidence are reported in Table 6.

**Table 6:** Summary of meta-analyses comparisons judged using the GRADE criteria to provide moderate quality evidence of effect of motivational interviewing.

Health behaviour	Review authors	Comparison	Population	Outcome	Assessment times	No of studies	n (total)	Effect size	Confidence intervals	Effect	GRADE Reasons for downgrade (GRADE judgement made by review or overview authors)
Alcohol	Vasilaki et al 2006[58]	other treatments	Any	Reducing alcohol consumption	unclear	9	?	ES 0.43	[0.17, 0.70]	**Beneficial**	Downgrade 1^a^ (overview)
	Foxcroft et al 2014[49]	No MI intervention comparison	young people (<25 years)	Average blood alcohol concentration (BAC)	4+ months	4	798	SMD -0.08	[-0.22, 0.06]	No benefit or harm	Downgrade 1^a^ (review)
				Binge drinking	<4 months	11	1340	SMD -0.23	[-0.42, -0.04]	**Beneficial**	Downgrade 1^c^ (overview)
					4+ months	16	4028	SMD -0.05	[-0.12, 0.01]	No benefit or harm	Downgrade 1^a^ (review)
				Drink driving	4+ months	4	1353	SMD -0.11	[-0.31, 0.09]	No benefit or harm	Downgrade 1^c^ (review)
				Frequency of alcohol consumption	<4 months	15	1928	SMD -0.26	[-0.44, -0.09]	**Beneficia**l (decrease in drinking days)	Downgrade 1^a^ (overview)
					4+ months	16	4390	SMD -0.11	[-0.19, -0.03]	**Beneficial** (decrease in drinking days)	Downgrade 1^a^(review)
				Peak BAC	<4 months	5	753	SMD -0.27	[-0.44, -0.11]	**Beneficial**	Downgrade 1^a^ (overview)
					4+ months	9	2042	SMD -0.14	[-0.23, -0.05]	**Beneficial**	Downgrade 1^a^ (review)
				Quantity of alcohol consumed	<4 months	22	2677	SMD -0.25	[-0.37, -0.14]	**Beneficial** (decrease in drinks consumed each week)	Downgrade 1^c^ (overview)
					4+ months follow-up	28	6676	SMD -0.14	[-0.20, -0.08]	**Beneficial** (decrease in drinks consumed each week)	Downgrade 1^a^ (review)
				Risky behaviour	<4 months	6	1048	SMD -0.09	[-0.30, 0.13]	No benefit or harm	Downgrade 1^c^ (review)
					4+ months	7	1781	SMD -0.14	[-0.30, 0.02]	No benefit or harm	Downgrade 1^a^ (review)
Physical activity	O'Halloran et al 2014[111]	Control (or usual care)	Any chronic health condition	Adherence	Immediately post-intervention	8	921	SMD 0.19	[0.06, 0.32]	**Beneficial**	Downgrade 1^d^ (review)
			Cardio-vascular disease	Adherence	Immediately post-intervention	2	115	SMD 0.22	[–0.15, 0.59]	No benefit or harm	Downgrade 1^d^ (review)
			Overweight/obese people	Adherence	Immediately post-intervention	4	498	SMD 0.14	[-0.06, 0.33]	No benefit or harm	Downgrade 1^d^ (review)
			Chronic health conditions	Functional exercise capacity	Immediately post-intervention	2	333	SMD 0.13	[-0.08, 0.34]	No benefit or harm	Downgrade 1^d^ (review)
Sexual health	Berg et al 2011[84]	control	Men who have sex with men	Sexual partners	unclear	3	4219	SMD 0.01	[-0.11, 0.13]	No benefit or harm	Downgrade 1^a^ (overview)*
				Unprotected anal intercourse	medium term	3	4191	SMD -0.04	[-0.10, 0.02]	No benefit or harm	Downgrade 1^a^ (overview)*
				Unprotected anal intercourse	long term	3	4021	SMD -0.02	[-0.08, 0.04]	No benefit or harm	Downgrade 1^a^(overview)*
				Unprotected anal intercourse (UAI) with non-primary partner	unclear	2	553	RR 1.04	[0.73, 1.47]	No benefit or harm	Downgrade 1^a^ (overview)
Smoking	Lindon-Hawley et al 2015[12]	brief advice/usual care	Mixed	Abstinence (strictest definition)	longest duration	28	16803	RR 1.26	[1.16, 1.36]	**Beneficial**	Downgrade 1^e^ (review)
Substance abuse	Smedslund et al 2011[56]	no intervention	people with substance abuse, dependency or addiction	Extent of substance use	short follow-up (0–6 months)	15	2327	SMD 0.17	[0.09, 0.26]	**Beneficial**	Downgrade 1^a^ (review)
		other active intervention	people with substance abuse, dependency or addiction	Extent of substance use	short follow-up	12	2137	SMD 0.02	[-0.07, 0.12]	No benefit or harm	Downgrade 1^a^ (review)
		other active intervention	people with substance abuse, dependency or addiction	Extent of substance use	medium follow up	6	1586	SMD -0.02	[-0.16, 0.13]	No benefit or harm	Downgrade 1^a^ (review)
		treatment as usual	people with substance abuse, dependency or addiction	Extent of substance use	post-intervention	9	1940	SMD 0.01	[-0.09, 0.11]	No benefit or harm	Downgrade 1^a^ (review)
		treatment as usual	people with substance abuse, dependency or addiction	Extent of substance use	short follow-up	10	2102	SMD 0.01	[-0.08, 0.10]	No benefit or harm	Downgrade 1^a^ (review)

Reasons for downgrading evidence

a-serious limitation in the Risk of bias

b-imprecision (e.g. wide confidence intervals or small sample size)

c- Inconsistency (e.g. high I^2^)

d–indirectness (e.g. variation in participants, intervention, comparisons or outcomes)

e–publication bias.

GRADE Working Group grades of evidence

**High quality**: Further research is very unlikely to change our confidence in the estimate of effect.

**Moderate quality**: Further research is likely to have an important impact on our confidence in the estimate of effect and may change the estimate.

**Low quality**: Further research is very likely to have an important impact on our confidence in the estimate of effect and is likely to change the estimate.

**Very low quality**: We are very uncertain about the estimate.

* Berg (2011) reported that they GRADED the evidence as low or moderate quality but no details were available in the publication other than a note to contact the authors for more detail. Therefore the overview authors judged the evidence.

Seventy one comparisons provided low quality evidence and 57 provide very low quality evidence judged by the GRADE criteria. S3 Table summarises the comparisons that were judged as providing low or very low quality evidence. The key reasons for downgrading the evidence to low or very low quality primarily relate to; risk of bias of the review was unclear; heterogeneity was judged to be moderate to high, or confidence intervals very large; volume of evidence was judged to be insufficient to support a definitive conclusion and concerns about the quality of the trials included within the comparison judged by review authors.

### Moderate quality evidence for effectiveness of Motivational Interviewing

Table 6 summarises the 27 comparisons, which provide moderate quality evidence for Motivational Interviewing interventions judged from six reviews [12, 49, 56, 58, 84, 111]. Eleven of these 27 comparisons (7% (11 of 155) of all meta-analyses’ comparisons) provide moderate quality evidence for mainly short term (<6 months) statistically significant beneficial effects of Motivational Interviewing. The remaining 16 comparisons demonstrate no benefit or harm, compared with a control of usual care or other active interventions. Moderate quality evidence of a beneficial effect of Motivational Interviewing was available for;

*Alcohol use*. 13 comparisons from two reviews [49, 58] explored the effect of Motivational Interviewing on outcomes relating to alcohol use in mixed populations. Eight of the 13 comparisons provide consistent evidence that Motivational Interviewing has a beneficial effect on outcomes relating to the frequency and/or volume of alcohol consumption, for short term outcomes (< 4 months), but the evidence relating to sustained (>4 months) outcomes is less consistence. Comparisons relating to risky behaviour and drink driving demonstrated no benefit (or harm) of Motivational Interviewing. There is evidence of beneficial effects from one review of young adults (<25 years), for reducing binge drinking, frequency, quantity of alcohol consumption and peak blood alcohol concentration[58].

*Smoking cessation*. One comparison from a review on smoking cessation was judged to provide moderate quality evidence. This review comparing Motivational Interviewing with usual care or brief advice, provides evidence of beneficial effects on abstinence from smoking, particularly when attention was paid to treatment fidelity[12].

*Substance abuse (drugs)*. One comparison from a review of people with substance abuse dependency and addiction provides evidence of a benefit of Motivational Interviewing when compared with no intervention. The other four comparisons derived no benefit or harm when Motivational Interviewing was compared with usual care or any other treatment [56].

*Physical activity*. Four comparisons from a review of Motivational Interviewing for promoting physical activity participation were judged to provide moderate quality evidence when Motivational Interviewing was compared with a control or usual care. One out of the four comparisons provide evidence of benefits. No benefit was found for the other three comparisons, including outcomes for people with cardiovascular disease and obesity [111].

*Sexual health*. Four comparisons from one review provide moderate quality evidence of no benefit or harm of Motivational Interviewing relating to changing high risk sexual behaviours in men who have sex with men[84] when compared with a control.

### Exploration of moderator variables

Of the six reviews that provide any evidence judged to be of moderate quality, three did not report the results of any subgroup analyses [56, 84, 111]. The three reviews that contain moderate quality evidence and report subgroup analyses are:

· Lindson-Hawley 2015 [12]–smoking cessation (Table A in S1 File)

· Foxcroft 2014 [49]–alcohol use in young people (Table B in S1 File)

· Vasilaki 2006 [58]–alcohol consumption (Table C in S1 File)

Exploration of the reported subgroup analyses provides consistent evidence which suggests that Motivational Interviewing is beneficial when compared to ‘weak’ comparison groups such as no treatment, assessment only or non-specified treatment as usual, but Motivational Interviewing is not beneficial when compared to other ‘strong’ interventions.

Generalisable conclusions relating to the most effective delivery of Motivational Interviewing (e.g. face-to-face or group), dose, or characteristics of provider or patient across behavioural domains are difficult to draw.

### Results of narrative reviews

Of the 104 reviews included in this synthesis, 65 did not combine any data within meta-analysis. The main findings from the narrative reviews are summarised in Tables 1 to 4. The majority focus on behaviour change in a general population, but also include people with specific mental and physical problems.

Narrative reviews of people with mental health problems include psychotic disorders[33], comorbid schizophrenia, combined mental health problems [31, 32, 35], general depression [10, 33–35, 69], post-stroke depression [36] and eating disorders [10, 69, 70]. One review in this category judged as low risk of bias suggests that Motivational Interviewing is important in psychiatric settings for reduction of substance use in the short term.

Narrative reviews of physical health problems include: cardiovascular problems (Motivational Interviewing for increasing physical activity) [83, 110]; musculoskeletal health (adherence with intervention for back pain) [103]; diabetes self-management (effect of smoking, blood-glucose control, diet and weight management [62, 75, 76, 78–80]; oral health hygiene[64–66, 68] (use of dental fluoride, increasing dental utilization and reducing sugar consumption); obesity (adherence to weight loss programmes); management of neurovascular disorders [82]. The most recent reviews report outcomes for the effectiveness of Motivational Interviewing for cancer care [101] and outcomes related to the treatment of irritable bowel disorder [104].

### Quality of narrative reviews

In total 20 narrative reviews were judged as low risk of bias graded using the ROBIS tool [15] [11, 20, 25, 35, 42, 44, 47, 51, 53, 54, 59, 65, 83, 89, 92, 97, 98, 113–115]. Five of these reviews report positive effects of Motivational Interviewing. Rueda et al (2006) found beneficial effects of Motivational Interviewing for adherence to highly active antiretroviral therapy where there appears to be promising results for interventions delivered over 12 weeks or more [97]. Taggart et al (2012) found further support for benefits of Motivational Interviewing in achieving impacts around smoking cessation compared to other group education [115]. Cooper et al (2015) reported positive results for some but not all outcomes for reducing cannabis use [42]. Noordman et al (2012) conclude that Motivational Interviewing can be effectively delivered by physicians and nurses as a face-to-face communication-related behaviour change technique[114]. Reviews published since 2016 report mixed results. Kay et al (2016) suggest that Motivational Interviewing has potential for use in oral care [65]. Chatters et al (2016) report short term benefits for reducing cannabis use in younger adults [47]. However, most were unable to make firm conclusions about effectiveness of Motivational Interviewing [20, 44, 59, 89]. In a review of brief non face-to-face Motivational Interviewing interventions Jiang et al (2017) found promising evidence for telephone delivery in the treatment of substance abuse, but the results were not consistent for other alternative modalities such as text messages in groups or internet-based interventions.

## Discussion

This overview is the first to integrate and systematically grade the quality of the evidence for the effectiveness of Motivational Interviewing interventions across a wide range of settings and populations for people with many different health problems and diseases. We have created a comprehensive map of all reviews relating to Motivational Interviewing to provide clarity relating to an intervention for which there have been multiple overlapping (and sometimes conflicting) reviews. Conflicting review evidence can create barriers and challenges to practitioners wanting to deliver evidence-based practice. This overview provides practitioners, policy makers and researchers with a summary of the quality and strength of the evidence for Motivational Interviewing. It signposts practitioners to the most up to date reviews, enabling them to efficiently access best review evidence to support clinical decisions. We found no high-quality evidence from the meta-analysis data within any review, mainly due to methodological flaws in the reviews and poor quality of the included studies.

Motivational Interviewing appears to be most effective for stopping or preventing unhealthy behaviours (categorised as Domain 1) such as binge drinking, reducing the quantity and frequency of drinking, smoking and substance abuse. For gambling behaviour, low quality evidence of short to long-term effectiveness suggests that further research on the effectiveness of Motivational Interviewing is warranted to address this significant public health problem [62]. For promoting healthy behaviour (categorised as Domain 2) where people may have little desire to change, most of the evidence is inconclusive or of low quality. For example, there is low quality evidence for the effectiveness of Motivational Interviewing for weight loss outcomes in obese and overweight adults. The exception in Domain 2 is physical activity promotion where there is moderate quality evidence of beneficial effects of Motivational Interviewing for increasing physical activity in people with chronic health conditions. However, the trials assessing adherence to physical activity participation were small and further high quality research in this field is justified to investigate the effectiveness of Motivational Interviewing in different populations, settings and context.

### Mode of delivery

The exploration of moderator variables from meta-analysis data does not provide enough data to be confident about the effects of different modes of delivery for Motivational Interviewing. Reviews that focus on the mode of delivery report inconsistent results [45, 51, 95, 105]. The TIDieR guidelines [14] capture some of the features that are relevant to intervention delivery but the mode of delivery is considered to be an important component of intervention and is not reported consistently in the literature [121]. Recent reviews have compared telephone [51] or technology-delivered Motivational Interviewing interventions (TAMIs) [105] and report inconsistent results or no beneficial effects. For example, Shingleton et al (2016) [105] found that TAMIs are feasible to deliver but there is limited evidence of effectiveness. For an intervention that relies on building and developing a relationship between client and provider it seems unlikely that this mode of delivery could be successfully adapted for Motivational Interviewing without considerable focus on training and fidelity measures.

### Implication for clinicians and policy makers

The National Institute for Health and Care Excellence (NICE) guidelines [2] include Motivational Interviewing as a component associated with some effective interventions for behaviour change strategies. However, the NICE (2014) Programme Development Group (PH49) are cautious about making general recommendations due to lack of details of intervention components reported in this field of research [2].

This overview has identified clear gaps in the evidence in support of most of the interventions categorised in Domain 2 (e.g. weight loss programmes for obesity, oral health behaviour, management of diabetes and musculoskeletal disorders, adherence to medication and engagement with interventions). The high quality reviews on smoking cessation [12] and alcohol abuse [49] both recommend caution when interpreting results. However, the overall effect size reported by Lundahl et al [108] of 0.22 (95% CI 0.17 to 0.27) is similar to other complex behavioural intervention [122, 123]. If applied to the 1 million smokers in the UK, or the millions of physically inactive people globally [124], it is plausible that the impact of Motivational Interviewing on health at a population level may be larger. Further rigorous research is required to support this assumption.

### Training and fidelity

Many different health care professionals including nurses, counsellors, physicians, medical students, social workers, and physiotherapists deliver Motivational Interviewing interventions, but there is little information about their training. Reviews that compared different health care providers found either no difference between groups [114] or reported limited conclusions due to small sample size [12].

Details of the fidelity of training of professionals delivering the interventions were generally poor although this is not unique to reporting of Motivational Interviewing. Training issues are fundamental to the success of any complex intervention and Motivational Interviewing, like other surgical, therapy or other behavioural interventions, requires practice of skills and a basic level of competency. There is no formal requirement for training in Motivational Interviewing or evaluation therefore practitioners can claim to use the approach without assessment, and competency is likely to influence outcome. Hall et al (2016) suggest that investment in training would need to be large to impact on change in practice [125].

It is difficult to comment on the cost effectiveness of Motivational Interviewing as it was not the focus of this overview, however we identified very little health economic data. Where cost data was available from a trial of smoking cessation in the UK, no clear conclusions could be drawn as the sustained quit rates did not reach statistical significance[12].

### Strengths and limitations of the overview

This overview is the first to synthesise systematic review evidence on the effectiveness of Motivational Interviewing from a wide range of populations and settings with an aim to provide information that informs practice and policy. It highlights the discrepancy between the widespread recommendations of Motivational Interviewing as a universal behaviour change strategy and the available evidence supporting this approach. We carried out a comprehensive search with an inclusive selection criteria and it is unlikely that we missed any reviews written in English prior to our initial search, but this overview is not exhaustive.

The conclusions of this overview are highly dependent on, not only the quality of the reviews but the studies within the reviews. We extracted data according to the TIDieR guidelines [14] but many intervention details were missing, making it difficult to draw conclusions with confidence. This problem needs to be addressed in future trials to facilitate data synthesis and provide clear recommendation to all stakeholders. Our assessment of review quality (ROBIS) [15] and evidence quality (GRADE) [17] are subjective judgements and we used these judgements to categorise the evidence, concentrating our conclusions on those judged to be moderate quality (or low bias for narrative reviews). Some may consider our methods overly critical, but authors of the higher quality reviews are equally cautious with their recommendations [11, 12, 49].

### Recommendations and implication for future research

The established Network of Trainers (MINT) alone have delivered Motivational Interviewing around the world to millions of people [126] but many questions remain unanswered regarding effectiveness.

#### Recommendations for clinical practice

Many different health professional groups are using Motivational Interviewing but the evidence for training reported in the literature is limited. The ‘Motivational Interviewing Treatment Integrity code’ (MITI) has evolved over the last 10 years [127] with an aim to standardise the delivery of Motivational Interviewing interventions. Guidelines for the minimum intervention content and training requirements for Motivational Interviewing are available and should be followed to standardise intervention delivery [127, 128].

#### Recommendations for future reviews

This overview has identified and brought together systematic reviews relating to Motivational Interviewing interventions; however further systematic reviews are warranted to inform clinical practice and future primary research in this field. Recommendations include, but are not limited to;

1. Research should address the fact that in clinical practice Motivational Interviewing is often delivered in combination with another psychological intervention. Systematic reviews exploring combined interventions were excluded from this overview; consequently, it is important to identify and appraise any existing systematic reviews relevant to this, prior to planning new reviews or primary research.
2. Future systematic reviews would benefit from the development of a taxonomy to ensure meaningful categorisation of the delivered intervention which considers the theoretical basis for Motivational Interviewing. Meaningful categorisation of Motivational Interviewing should be central to informing clinically relevant analyses and subgroup analyses.
3. A systematic review to explore the cost-effectiveness of Motivational Interviewing as an intervention for those health conditions where there is moderate quality evidence of a beneficial effect of Motivational Interviewing on patient outcomes.
4. A systematic review to explore the barriers and facilitators to delivery of Motivational Interviewing, focussed on those health conditions where there is moderate or high quality evidence of a beneficial effect.
5. A systematic review of qualitative evidence to explore the acceptability and perceptions of this intervention to people who are offered Motivational Interviewing.
6. Stakeholder involvement should be conducted in future reviews of the Motivational Interviewing literature particularly relating to categorising interventions and outcomes.
7. The use of reporting templates, recognised guidance and best practice for the conduct of systematic reviews and primary research is essential. e.g. PRISMA [129] and TIDieR [14].

#### Recommendations for future primary research

1. Exploration of the effect of Motivational Interviewing should consider long-term outcomes and cost-effectiveness. Subgroup analyses should explore the length of intervention delivery and time since the end of the intervention.
2. Investment in training would need to be large to impact on change in practice [130] and this along with other issues relating to sustainability of the intervention e.g. context, should be considered in future trials.
3. To ensure avoidance of research waste [131, 132] it is essential that researchers are fully aware of existing reviews before embarking on further reviews, and that critical systematic reviews of evidence are completed prior to further primary research.

## Conclusion

For the health problems that Motivational Interviewing was originally developed to address such as smoking cessation and alcohol misuse, the evidence provides some support for implementation particularly if fidelity of the intervention is prioritised. However, Motivational Interviewing has been implemented already for a wide range of other health and social problems where a “one size fits all” approach has been adopted with inconsistent effects.

## Supporting information

S1 Checklist PRISMA checklist. (DOC)

S1 Table Characteristics of interventions according to TIDIER checklist reporting guidelines. (DOCX)

S2 Table Quality assessment of included reviews based on ROBIS (risk of bias in systematic reviews) tools. (DOCX)

S3 Table Summary of comparisons judged to provide low or very low quality evidence. (DOCX)

S1 File Exploration of moderator variables. (DOCX)

S1 Appendix Medline search string. (DOCX)
