# Supplementary material for: Effectiveness of Motivational Interviewing on adult behaviour change in health and social care settings: A systematic review of reviews
Source: PLoS One. 2018 Oct 18;13(10):e0204890. doi: 10.1371/journal.pone.0204890 (PMC6193639; doi:10.1371/journal.pone.0204890)
Supplement: S1 Table — (DOCX) [file pone.0204890.s002.docx]

# S1 Table: Characteristics of interventions according to TIDIER checklist reporting guidelines

MI = Motivational Interviewing; MEI = Motivational Enhanced Interviewing, Brief Motivational Interviewing = BMI; Motivational Interviewing Network of Trainers (MINT); Motivational Interviewing Treatment Integrity (MITI)

| **Domain 1 – Smoking / Tobacco use** | | | | | | | | | |
| --- | --- | --- | --- | --- | --- | --- | --- | --- | --- |
| **"First author (year)"** | ***NAME/ DEFINITION OF INTERVENTION*** | | ***WHAT Materials were used and where accessed?*** | ***Procedures*** | ***WHO DELIVERED THE INTERVENTION?*** | ***DETAILS ABOUT THE INTERVENTION PROVIDER*** | ***HOW? Mode of delivery*** | ***WHERE: LOCATION*** | ***WHEN AND HOW MUCH?*** |
| Baxi, et al (2014) [1] | Family and carer smoking control programmes including MI | | Varied (e.g. pamphlet and sticker depicting a smoke-free home; self-help materials targeting ETS reduction and smoking cessation strategies were also provided. Other information brochures (no details) were also supplied to control groups. | Varied across the 11 trials and little information provided about specific MI intervention. | Nurse counsellor, trained health educator, nurses | Unclear | Intervention delivered face-to-face in majority of studies but follow-up usually by phone | Majority of studies from USA (n =33). Canada, Australia, UK, Europe and China. Community level. The majority of studies targeted parents within healthcare contexts, with 23 targeting parents in ’well child’ settings and 24 reporting interventions in ‘ill child’ healthcare settings." | Length of MI sessions ranged from 10 - 45 mins; number of sessions also varied from 1-4, with a number of the follow-up sessions taking place by phone. |
| Baxter, et al, (2011) [2] | MI | | Unclear | MI intervention was followed by four telephone counselling calls. A key component of the intervention was feedback from baseline household air nicotine assessments followed by tailored goal setting | Trained health educators | No specific training mentioned | Face to face, 4 telephone counselling sessions | 1 study including MI =USA) Others 10 =USA United States and Canada European countries including two from Sweden one from Finland, Italy, 1 = UK, 1 = China | One 30- to 45-min motivational interviewing session followed by four telephone counselling calls. |
| Behbod et al (2018) [3] | MI and BMI. | | Several studies combined motivational  interviewing with other frameworks and other training not well described | Process indicators provided information regarding the  integrity of the way in which interventions were implemented.  However, only 32 of the 78 studies described process indicators. 1 study conducted focus groups to better understand Latino  culture and to modify the motivational interviewing technique accordingly. | Nurses/ nurse counsellor/ paediatric respiratory physician/ psychologist | no specific training mentioned | Varied, 1 face-to-face/on phone | 27 studies in well-child healthcare setting; 26= ill-child healthcare setting. 2 + paediatric clinics.  45 studies = USA, 22 from other high-income countries, and 11 from low- or middle-income countries. | Varied, mainly brief e.g. 30 minutes with nurse counsellor;  appropriate stage-matched intervention used to “increase motivation and lower  resistance to quit”; telephone reminder 1 week after the intervention |
| Ebbert et al (2015) [4] | MI | | Manuel and video | Invited to receive mailed manual and video during a telephone call using a MI style. Two further 10 min support calls after receipt of materials and telephone call using a MI style. | Unclear | Unclear | Unclear | USA | 1 initial telephone conversation followed by a 10 minute support call |
| Heckman et al. 2010 [5] | MI for smoking cessation | | Pamphlets, videos | "Most (74%) MI arms combined MI with some additional intervention. These included 12% personalized risk feedback (e.g., carbon monoxide level), 6% educational pamphlets, 12% other types of interventions (e.g., CBT, relapse prevention, videos), and 44% included multiple other interventions (e.g., feedback and pamphlet)” | "Of the 33 MI arms in which data were reported, providers were 36% counsellors/therapists, 18% staff/interventionists, 12% nurses/ midwives, 9% mixed, 6% psychologists, 6% physicians, 6% health educators, and 6% trainees". | "mean duration of staff training in MI was 52 hours (reported for 11 MI arms)" | Unclear | "Seventy-one percent of the studies were conducted in the USA (all others were conducted in Europe and Australia), and sixteen percent of the studies were cluster randomized (e.g., by provider or clinic)." | "Mean duration of the MI interventions was 101 minutes (reported for 32 MI arms)". |
| Hettema, & Hendricks (2010) [6] | MI for smoking cessation | | Unclear | "Wide variability in the administration of MI across the 23 main studies. MI was combined with a variety of other intensive and minimal active treatments in the majority (74%) of trials. Of the included studies, 30% combined MI with some form of pharmacotherapy, and 13% combined MI with a skills-based behavioural intervention such as CBT or relapse prevention". | "MI was delivered by range of providers, including physicians, psychologists, master’s level counsellors and social workers, nurses, and health educators". "Of the studies that reported intervention agents, mental health and medical providers were fairly evenly represented."" | "As a proxy to intervention training, studies were coded as mentioning or not mentioning the training of MI interventionists, and 16 out of 23 studies (70%) made such mention. Duration of training was reported in seven studies and ranged from 2 to 75 hr (M 28.14, SD 25.89)". | "All included studies used human interventionists to administer MI, although several studies conducted the intervention via telephone or computer." "MI was also conducted in a variety of formats, including individual and group, and several studies had some telephone based component." | "MI conditions were administered in a variety of settings, including specialty and primary care medical settings, emergency departments, residential treatment programs, schools, patients’ homes, and research clinics. Overall, medical settings were the most common site of implementation." Studies undertaken in USA, Australia, Northern Ireland, Sweden and Spain. | "The duration of the MI treatment condition also varied widely across studies, with participants assigned to this condition attending a mean of 5.50 (range 1–24, SD 5.21) treatment sessions, across 12.7 weeks (range 1–72, SD 18.52). Average session duration was 29.93 min (range 10–60, SD 29.93), with MI participants spending a total of 188.11 min (range 10–635, SD 195.30) in treatment on average. Average number of minutes in comparison condition treatments was much shorter (M 11.10 min, range 0–62.5, SD 16.71)." |
| Lindson- Hawley et al (2015) (update of Lai et al 2010) ( [7] | MI for smoking cessation | | Unclear | "Most commonly used approach to motivational interviewing (MI) has been one in which the smoker is given feedback intended to develop discrepancy between smoking and personal goals in a non-threatening manner". | Primary care physicians, hospital clinicians, nurses or counsellors. | Unclear | MI was delivered in face-to-face sessions in all the studies except for 3 studies, in which the counselling was telephone-based. Individual - none of the included studies used MI in groups. | USA, UK, Spain. Settings included general practices; one in participants’ homes and one was delivered through a telephone quitline service. Two programmes were provided through screening clinics, four in specialist outpatient clinics (Glasgow 2000; Curry 2003; and four in hospitals. | "Trials were conducted in one to four sessions, with the duration of each session ranging from 15 to 45 minutes. All but two of the trials used supportive telephone contacts and supplemented the counselling with self-help materials". |
| Mantler and Morrow (2012) [8] | MI strategies in smoking cessation programmes | | Text messages, Internet site, Quitline, (a telephone hotline), weekly calls to a check in and track progress, and medication (e.g. Nicotine replacement therapy), health education materials (booklet and video), workbook and four audio CDs, | Varied. Included text messages; a hotline, proactive calls from counsellors, group sessions targeting "preparation for quitting, quitting, and maintenance/ relapse prevention or clarifying value of quitting and acceptance of quitting", check in phone calls, medications, treatment sessions, health education materials (booklet and video). | Counsellors | Unclear | Face-to-face; telephone, text and internet; individual and group sessions reported across included studies. | Unclear | Varied. E.g "group-based treatment consisting of six weekly sessions based on the “withdrawal- oriented” model of cessation and assigned buddy", |
| Pelletier et al (2014) [9] | MI | | 3 x brochure on smoking cessation; 1x Computerized screening, advice to quit, MI counselling in ED, 4 telephone follow-up sessions | varied; most include brochures on smoking cessation, 1 x Computerized screening, advice to quit, MI counselling in ED. | Unclear | Unclear | Internet; face to face( not specified but most likely) phone call follow up x 3 | USA ( not specified in text) | Brief interventions 13-30 minutes; 3-4 follow up call in 2 studies. 3-6 month follow up of trial. |
| Rabe et al (2013) [10] | MI in 4 out of 7 studies | | Phone calls, workbook with audio; pamphlets | MI on site; workbook with audio, booster phone calls, self-help brochures, | Physicians or nurse | provided | Face to face, group and telephone. 1 x MI and workbook and personal postcard; 1 x MI +2 booster phone calls; 1x MI + 3 booster phone calls; 1 x MI + 4 booster phone calls | 6 x USA 1 Germany (4 used MI 1 x Germany 3 x USA) | Varied 1-4 single sessions and follow up at 3-12 months |
| Stead et al (2016) [11] | Behavioural support including MI (e.g. brief advice, tailored materials, in person or telephone counselling) combined with pharmacotherapies | | Mail or pre-recorded phone messages; phone calls | The typical intervention involved multiple contacts with a specialist cessation adviser or counsellor, with most participants using some pharmacotherapy and receiving multiple contacts. However, there was a great deal of variation | Specialist cessation counsellors or trained trial personnel, GPs /family physicians, nurse counsellor, trained nurse, pharmacist, mental health professional or Dentists/ dental hygienists; peer group counsellor (ex-smoker), or lay advisor. | Unclear | One intervention was delivered  by mail or pre-recorded phone messages, using an expert system for tailoring contact, and two by telephone  counselling alone. All others included face-to-face contact sometimes provided by telephone. | Half the studies were conducted in the USA. Of the others, Canada x5, Australia x4, Denmark x3, Spain x3, UK x4 and one from Brazil the Netherlands, Sweden, Japan and Hong Kong. Recruitment in community settings | More than half the trials (n = 28, 53%) offered between four and eight sessions and a quarter (n = 13) over eight sessions. The planned maximum duration of contact was typically more than 30 minutes but less than 300 minutes. |
| **Domain 1 – Substance abuse (alcohol and drugs)** | | | | | | | | | |
| **"First author (year)"** | | ***NAME/ DEFINITION OF INTERVENTION*** | ***WHAT Materials were used and where accessed?*** | ***Procedures*** | ***WHO DELIVERED THE INTERVENTION?*** | ***DETAILS ABOUT THE INTERVENTION PROVIDER*** | ***HOW? Mode of delivery*** | ***WHERE: LOCATION*** | ***WHEN AND HOW MUCH?*** |
| Appiah-Brempong et al (2014) [12] | | MI and interventions  that used adaptations of MI  underpinned by the key principles of MI | Unclear | "MI with feedback component  (MIF) used in some trials " | Unspecified practitioners | Unclear | 6 studies face to face one-on-one intervention, 4 group sessions and 3 unclear | Unclear | Unclear |
| Barrio and Gual (2016) [13] | | MI and BMI | Unclear | All psychosocial interventions based on motivational interviewing (MI) principles, MI is defined as “a directive, client-centred counselling style for eliciting behaviour change by helping clients to explore and resolve ambivalence”. | Nurses / Primary care physician / Unspecified practitioners | Unclear | Face to face individual session and telephone | Mainly USA, Holland, Denmark, Switzerland, Poland, Canada.  ED department and student campus | Varied. Most BMI 1 session with follow up telephone call. 15 studies include >1 session. Time varied from 10 minutes to 90 for 1 session. |
| Branscum, et al (2010) [14] | | MI based interventions” | Various. Checklists, take-home journals, audio-recording equipment, financial incentives e.g. (one study 'forgave' an outstanding fine if participants completed the programme) | Various. Mandatory education-based program studies; "brief MI-based intervention (basic BMI) with two separated additive features; a timeline follow-back assessment (TLFB) before the intervention to raise awareness of the individuals drinking behaviours, and a decision balance activity (enhanced BMI), to raise awareness of pros and cons of heavy drinking". Another study reported the use of "‘‘Drinker’s Check-Up’’ intervention consists of individual MI sessions, using personalized feedback." "Peer-enhanced MI-based intervention (individual MI session, with an addition of a close peer." | Referred to as the "interventionist" | Unclear | Unclear but some studies reported individual session and group sessions. Face-to-face is implied in the text but not explicitly stated. | Unclear | Varied. Duration of intervention ranged from 30 -120 mins. "Most studies had adequate follow-up assessments of at least 3 months", with one study reporting follow-up at 12 months. |
| Carey et al (2007) [15] | | Individual-level interventions including MI | Most interventions were informed by theory (82%) and guided by manuals (61%); manuals were used in 66% of the interventions delivered face-to-face, 46% of the interventions delivered via computer or print, and 57% of the combined intervention delivery modes." Alcohol-related materials (e.g., brochures, pamphlets) were provided in 47% of the interventions". | MI techniques were used in 44% of the interventions. Intervention components frequently included alcohol/BAC education (73%), normative comparisons (56%), and feedback on consumption (49%); they often included moderation strategies (43%), feedback on problems (37%), goal setting (35%), or feedback on expectancies and/or motives (34%). Less frequent components included; identification of high-risk situations (24%), decisional balance exercises (17%), skills-training (16%), an expectancy challenge (12%), or values clarification (7%). Alcohol-related materials (e.g., brochures, pamphlets) were provided in 47% of the interventions." | Varied. "Of the 73 interventions delivered face-to-face, session leaders were professionals-in-training (66%), professionals (21%), peers (18%), or paraprofessionals (12%); some interventions used more than one type of facilitator." | Unclear | Most interventions involved face-to-face delivery by a facilitator to an individual or a group (70%), with a minority using computer or print delivery (22%), or a combination (7%) | United States (85%). Most studies were conducted at public universities (72%) of large size (>10,000; 89%) | "Interventions delivered in groups (44%) consisted of a median of 2 sessions, of 50 minutes each, with a median of 1 facilitator and 9.8 participants; individually delivered interventions (40%) consisted of a median of 2 sessions, of 50 minutes each, with a median of 1 facilitator and 1 participant; and interventions with no face-to-face contact (e.g., computer/internet, mailing; 30%) consisted of a median of 1 session of 15 minutes with no facilitators and 1 participant." |
| Carey et al (2012) [16] | | Most of the FTFIs evaluated consist of brief motivational interventions (BMIs; single-session, feedback interventions conducted in motivational interviewing style), | 25 interventions were guided by BASICS.  general alcohol-related material used in some studies | Alcohol education, feedback on consumption as well as alcohol related risk factors and problems, and normative comparisons | peers = 3); Parents =1); Paraprofessionals (8); Professional -in training =25; Professionals' =7 |  | Individual face to face | USA | Single sessions 5 to 120 minutes for FTFIs; most FTFIs ranged between 30 to 60 minutes, with a cluster at 50 to 60 minutes. |
| Chatter et al (2016) [17] | | MI/MET/BMI | Unclear | Unclear | Unclear | Unclear | Face to face, telephone | Inpatient setting (i.e. study participants are resident within  A ward or treatment facility. | Unclear |
| Cooper et al (2015) [18] | | MI/motivational enhancement  therapy (MET). | Unclear | Unclear | Unclear | Unclear | Individual face to face and group | USA (13 studies), Australia (7), Germany (3), Brazil (2), Canada (2), Switzerland (2), Denmark (1), Ireland (1) and multicounty (2). | Ranged from 1-4 sessions , 1 condensed to 1 week others over time |
| Darker et al (2015) [19] | | MI/motivational enhancement  therapy (MET). | A detailed manual was developed one study that drew from existing MI manuals and guides and adapted them to be  used in the single-session format and which anticipated a participant sample with a wide  range of substance use problems | MI strategies (e.g. practicing empathy, providing choice, removing barriers,  providing feedback and clarifying goals) and that used an MI interviewing style (e.g.  Psychosocial interventions for benzodiazepine harmful use, abuse or dependence | Trained therapists | “Trained” no further details | Face to face, telephone, letters | Two studies took  place in opiate dependency clinics (34 participants) and the other  two in the acute hospital setting, gynaecology (39 participants) and  psychiatry (seven participants). Studies were conducted in Norway, the  Czech Republic, USA and Germany. | Ranged: some sessions included participated in an approximately  2 hour assessment |
| Gates et al (2016) [20] | | Motivational interviewing/motivational enhancement  therapy (MET). | Unclear | MI approaches tend to emphasise the importance of self-efficacy and positive change and attempt to build motivation in an empathic and non-judgemental environment (Miller 2002). This approach is often enhanced by personalised feedback and education. |  |  | Face to face |  | Ranged from 2 sessions to 14. |
| Joseph et al (2014) [21] | | Nurse conducted brief intervention (NCBI) | Health promotion booklet | "BI comprised several interventions, in the form of simple structured advice on general health issues related to alcohol, motivational  counselling, health promotion booklet, or combinations of these." | Nurses. 2 trials that specifically included MI delivered by a nurse or counsellor. | Psychiatric nurses (n = 3 trials), nurse practitioners (n = 2 trials), research nurse (n = 1 trial) and who had experience from primary care, outpatient care of alcoholics, oral and maxillofacial surgery department, emergency department. Physician x 2 trials, off-site alcohol counsellor x1 trial. All the trials reported a separate training programme in BI for all the nurses or the therapists to provide an individualized intervention. | Face to face | "Three trials took place in the UK, two in Australia, one each in USA,  Canada, Scotland, Sweden, China and Taiwan. Most of the interventions (n = 7 trials) were administered in general practice-based  primary care and clinics. 3 trials were  carried out in hospital inpatient settings and one trial in accident and emergency department." | "Intervention exposure ranged from 5 to 30 min.  Most trial evaluated a single BI session, and two trials used two to three sessions during a 12 months follow-up  period." |
| Jiang et al (2017) [22] | | MI or alternative mode of MI | Unclear | “Telephone delivery was the most commonly used alternative medium for MI (11 studies) followed by Internet (4 studies)). Group MI was tested in 5 studies, with a group size of 4–10 participants. In 8 studies, other behavioural interventions were combined with MI, such as cognitive behavioural therapy motivational enhancement therapy” | “The MI counsellors included nurses or nurse health educator, clinicians, psychologists, psychiatrist, students in related areas (health sciences, public health or psychology), research assistant, and social worker.” | 3 out of 9 trials included a fidelity measure. 1 used Audiotaped, reviewed and rated by MITI and MISC .2 others used audiotapes, 1 used questionnaire.  4 trials included some sort of training. | “Telephone was the most frequently used medium for delivering MI (11 studies), followed by Internet communication (4 studies) and short message service (SMS) (2 studies)”. | 1 included emergency department and college students | Sessions duration varied from 1 session of 20 minutes to various combinations including 24 sessions (a series of text messages) The number of main MI sessions ranged from 1 to 24. Single session MI was adopted in 5 studies. Duration of follow up varied from 8 weeks to 1 year, with 15 trials reported 6- month follow-up or longer. The attrition rate ranged from 2% to 74%. |
| Joseph and Basu ( 2017) [23] | | Brief motivational counselling sessions based on the principles of motivational interviewing | Unclear |  | The professionals involved in the delivery of brief interventions were nurses (63%) and other health professionals such as social workers, psychologists and research assistants (27%). | All the trials reported the quality assurance of intervention by incorporating the training for the professionals involved in brief intervention. | Face to face | One study carried out in India, 2 in Thailand, 4 in South Africa. | Sessions ranged from 10 to 20 min. The duration of individual sessions in three trials varied from 45 to 60 min. |
| Klimas, et al (2012) [24] | | Psychosocial interventions in problem alcohol use in illicit drug users | A written change plan, ("designed to reduce the link between alcohol consumption and hazardous behaviours that may lead to negative consequences of drinking, including HIV risk behaviour")  NS | MI: focused on alcohol use and HIV risk-taking in 1 trial. In the second trial, there were two MI arms: (1) MI-group: focus: alcohol, risky behaviours, MI spirit. Content of the individual and group sessions was identical, guided by a detailed protocol and biweekly meetings with the investigator and therapists. | "PhD-prepared psychologist and a researcher" | "Interventionist trained by studying the manual and watching MI tapes from Project MATCH" | Face-to-face in both MI studies but one trial compared group vs individual MI. | "three studies were conducted in USA and one in Switzerland" 2/4 trials reported have used MI or BMI as the intervention and were conducted in an opioid substitution clinic setting in one study and in a needle exchange programme in the other. | 2-3 therapist sessions, sessions 30-60 mins. Follow-up ranged from 1-6 months |
| Foxcroft et al (2014)[25] | | MIs approach including MI principles (an empathic non-judgemental stance, listening reflectively, developing discrepancy, rolling with resistance and avoiding argument, supporting efficacy to change) as the core of the intervention as well as a feedback element or other non-MI techniques. | 1 study referred to using interventions modelled on the BASICS intervention, and involved the delivery of personalized feedback in an MI-based framework | MI used in all studies; In one study the treatment was facilitated by a computer that displayed screens to prompt sections of content for the therapist to deliver, including tailored feedback. For each intervention a key component was described. E.g. contracting and goal-setting, diary cards and take-home exercises; (a) Opening strategy: lifestyle and alcohol use, alcohol use within a typical day session; (b) The good things and the less good things about drinking alcohol (decisional balance); (c) Evoking a hypothetical change; (d) Exploring importance, ability, and confidence to change; and (e) Eliciting commitment to change, identification of an eventual change. Contracting and goal-setting, diary cards and take-home exercises; The intervention considered 12 points to be discussed during the session: (1) contact, (2) feedback from the evaluation, (3) analysis of an episode of substance-use, (4) pros and cons of substance-use, (5) personal goals, (6) problems and risks of substance-use, (7) explore preoccupations, (8) making decisions, (9) questions and answers, (10) decisional balance, (11) planning changes, (12) self-monitoring. | Unclear | Unclear | In 49 of the trials the MI consisted only of 1 face to face individual session. In 12 studies young people attended mixtures of both individual sessions and group sessions. Five studies used group MI sessions only. | 56 trials in the USA, four in the UK (one in Australia (two in Switzerland (one in Spain (one in Brazil and one in Canada and the USA. Settings for the trials varied and 37 of the 66 studies took place on college (university) campuses. The remaining 29 studies took place in further education colleges, a youth centre, local companies, a job-related training centre, with army recruits, UK drug agencies, and youth prisons | In 43 trials sessions took one hour or less; the shortest was a single 15 minute intervention  (Doumas 2008) and the longest (Nirenberg 2013) had five MI sessions over a 19 hour period. One study (Barnett 2007) reported a “brief ” intervention without specifying a duration, and five studies did not specify any information at all about session duration |
| Gilinsky, et al (2011) [26] | | Only one pilot intervention described using MI to reduce alcohol consumption during pregnancy. | Unclear | The study implemented a 1-h single session of face-to-face MI amongst women that had reported drinking during pregnancy in the past month. The authors gave participants personalised feedback on the severity of alcohol consumption and encouraged them to quit drinking at any point during pregnancy. | Principle investigator (unknown profession) |  | face- to- face | (1 MI study was carried out in USA) others 4 =USA, 2=Up, 1=Norway | 1 hr session between baseline assessment and 8 week follow up |
| Livingston, et al (2012) [27] | | Social stigma intervention including "motivational interviewing" | Unclear | Unclear | Unclear | Unclear | Unclear | "Majority of studies were conducted in the US (n=7), with the remaining studies conducted in the United Kingdom, Canada and Australia". The MI study took place in the community. | Unclear |
| McMurran, Mary 2009 [28] | | MI defined as "directive psychosocial intervention used to identify and resolve discrepancies between desired behaviours and actual behaviours, and to increase motivation to facilitate behaviour change (Miller & Rollnick, 2002)". | 1 study include financial incentive to participate and adhere to intervention | Varied, some programmes included CBT alongside MI and Drug counselling. | Probation officers with and without training, | Unclear | Face to face and group | USA , 2= UK, 2= Canada, 1=New Zealand | varied delivery ; group MI for 12 weeks, 8 weeks,, 6 sessions, 1 session of 90 minutes, 1 session of 45-60 minutes, 2 sessions. |
| Seigers, et al 2010 [29] | | “most interventions used motivational interviewing and/or personalized feedback. Two studies also used timelines or calendars to raise awareness of drinking patterns. Six studies (50%) had Web-based components and/or used computerized assessments for screening and/or intervention”. | “Timelines or calendar to raise awareness of drinking patterns. Six studies (50%) had Web-based components and/or web based programme “ | Empirically supported techniques, primarily motivational enhancement and personalized feedback. | “(a) trained research staff that included interventionists outside the recruitment site or by (b) onsite staff that included primary caregivers (e.g., nurse practitioners, physicians) or counselling centre staff” | Unclear | face to face and computer delivered | New Zealand x 2. Ref list suggest mainly USA. college- or university based student health centres or emergency departments | Brief intervention less than 75 minutes. The majority of studies only measured outcomes at 1 time point, usually 3 months or earlier. |
| Smedslund, et al (2011) [30] | | “The intervention has four basic principles. A brief variant of MI is called Motivational Enhancement Therapy (MET). MET is manual-based, and was developed as part of Project MATCH (Project MATCH 1997).” | Training by a member of MINT Motivational Interviewing Network of Trainers was used in some studies  <http://motivationalinterview.org/clinical/principles.html> | The intervention could be offered in three ways: (1) as a stand-alone therapy, (2) MI integrated with another therapy, or (3) MI as a prelude to another therapy (e.g. cognitive behavioural therapy). | Counsellors and therapist (no other details) | Unclear | Face to face only. Excluded group interventions and computer/  telephone interventions | 44 studies in USA, five in Australia, three in Netherlands, three in UK, two from Canada, and one each from Germany and New Zealand. | Various and typically lasting for 1-4 sessions |
| Tanner-Smith et al (2015) [31] | | MI and MET | Unclear | Unclear | Varied vary widely in terms of background training, education level, and therapeutic expertise; e.g., undergraduate peers trained by research staff, trained professional clinicians, | Personnel characteristics inconsistently reported across studies | Face to face and computerized self-administered interventions., group, or family session | Mainly in the United States in university settings. Other delivery sites: primary health care or university health centres, emergency room or self-administered. | Intervention aimed at providing motivation for behaviour change in a relatively circumscribed time (1-5 sessions). treatment duration (single session less than 5 minutes, single session of 5-15 minutes, single session longer than 15 minutes, or multi-session). Interventions for young adults (55 total minutes, 3 days covered). |
| Terplan et al (2007) [32] | | MI +MET (motivational enhancement therapy (MET) a standardised form of MI). 3 studies included brief MI intervention. |  |  |  |  |  | All USA except 1 took place in Australia. All drug treatment facilities that were either academic- based, or hospital-based, or both.  Predominately outpatient. | 1 = three one hour MI sessions. 1 = a total of six session lasting 60-90 minutes each. The first session was MI, In Haug 2004, the most standardized form of MI was employed, motivational enhancement therapy (MET). This involved four sessions each tailored to the individual’s stage of change. |
| Terplan et al (2015)[33] | | MI +MET (motivational  enhancement therapy (MET) and MIB | Unclear | Unclear | Mental health providers all with formal training in MI (Mullins 2004); other MIB trials. | Varied widely in terms of background training, education level, and therapeutic expertise; e.g., undergraduate peers trained by research staff, trained professional clinicians, and computerized self-administered interventions | Face to face. | USA x13, Australia x1. Outpatient drug treatment facilities (academic-based, or hospital-based, or both). sites where these psychosocial treatments were delivered also included supplementary services for both the experimental and control groups, including child care services, transportation, and housing. | treatment duration (single session less than 5 minutes, single session of 5-15 minutes, single session longer than 15 minutes, or multi-session). Interventions were notably longer for adolescents than for young adults (100 versus 55 total minutes, 6 versus 3 days covered). |
| Vasilaki, et al 2006[34] | | Brief intervention delivered according to the principles of MI were selected on the basis of Miller and Rollnick’s (2002) definition of MI and briefest interventions (30 mins) | Unclear | Procedures met the following criteria: (i) claimed to adopt the principles and techniques of MI as described by Miller and Rollnick (1991), | 1 nurse; 1 therapist; other clinicians and PhD students | No details of specific training but registered as having had training in Table | Face to face. | USA. 6 = outpatient community settings; 5 = emergency-room or clinic settings with patients reporting alcohol-related problems, such as a physical injury; 2 studies examined the efficacy of MI in specialist substance-abuse treatment agencies | Varied from 15 minutes to 240 minutes MI. Follow-up assessments varied and conducted at 3, 6, 12, and 24 months. |
| **Domain 1 Substance misuse in people with co-existing mental health problems** | | | | | | | | | |
| **"First author (year)"** | | ***NAME OF INTERVENTION*** | ***WHAT Materials were used and where accessed?*** | ***Procedures*** | ***WHO DELIVERED THE INTERVENTION?*** | ***DETAILS ABOUT THE INTERVENTION PROVIDER*** | ***HOW? Mode of delivery*** | ***WHERE: LOCATION*** | ***WHEN AND HOW MUCH?*** |
| Baker, A. et al (2012) [35] | | Motivational Interviewing, as well as Cognitive Behavioural therapy (CBT) and psycho-educational. | Unclear | Motivational interviewing, cognitive behavioural therapy, and educational intervention in combination and delivered separately. Entry criteria included use of a treatment manual for excessive alcohol consumption, but no details were given. | Psychologist (n=5), nurse therapist ( n=1), social workers (n=3), clinic staff (n=1) | Unclear | face to face varying in length of time and follow up | Locations were in USA (3), Australia (3), Netherlands (1) and Canada (1). Combination of Voluntary (n=5) and non- voluntary Outpatient clinics (N=1) and inpatient clinics ( n= 3) | Sessions varied from 1, 30 -45 minute, 3 x 1 hour indicial, 2 x 1 hour individual, 10 x 60 min weekly, 4-6 hours individual, up to 26 x 60 minute sessions. Follow up varied between 3 months, 6 months and 1 trial followed up to 18months |
| Baker et al (2012) [36] | | Motivational Interviewing (no further description) | One RCT used a template guided intervention but no mention of source | 1 RCT =1 initial integrated session comprising MI and case formulation + 9 extra sessions of MI and CBT; 1RCT included group MI consisting of 2 2hourly sessions. 1RCT included a 45 minute single template guided MI session | 1 = nurses or clinical psychologists, 1=psychologist, 1= group session by therapist | No specific training mentioned | Face to face in 2 RCTs and group session in 1 RCT | 2 in Australia, 1 in USA others not including MI in treatment = Netherlands, USA and Australia | Varied across studies 1= 45 Minutes, 1 = 2 120 minute sessions group MI , 1 60 minute session face to face plus 9 60 minute sessions of MI |
| Boniface et al (2018) [37] | | MI | One RCT included web-based survey  Providing personalized web- based feedback. | Varied. Most included motivational interviewing style principles. I study followed by a personalized  feedback letter | Mental health providers / clinicians | no specific training mentioned | Majority face-to-face fewer  trials delivering BI through web, phone or blended methods | USA (10 trials),  Australia (2 trials), Sweden (2 trials), UK, Norway and Germany (1 trial each). | Varied but all brief e.g. / 15–20-min session,  followed by a personalized  feedback letter; One 45-min session, using MI  techniques, followed by two 15-min booster telephone sessions |
| Cleary et al 2009 [38] | | Motivational interviewing in people with a dual diagnosis | Unclear | Simply referred to as MI, with a general introduction to MI in the introduction, but not clear whether MI was adapted or adhere to any manualised intervention. | Unclear | Unclear | Face-to-face, individual and group sessions | Inpatient and outpatient settings; Australia, USA | Varied. Duration of sessions ranged from 1-3 hours delivered over 1- 9 sessions. Limited details reported in Table 2. |
| de Man-van Ginkel et al 2010 [39] | | MI | Unclear | The personal recovery goals and perceived barriers were discussed in individual sessions. Therapists worked with patient’s dilemmas and ambivalence and enabled them to identify their own solutions by supporting and reinforcing optimism and self-efficacy (Watkins et al. 2007) | n = 1 therapist unknown | Unclear | face to face | Unclear | Four individual sessions in four following days |
| Hjorthoj, et al (2009) [40] | | MI | Unclear | Unclear | Unclear | Unclear | Unclear | USA, Nordic countries, Canada, Brazil, Belgium, Australia and Taiwan |  |
| Kelly et al (2012) [41] | | MI | Unclear | Unclear | Unclear | Unclear | Unclear | Unclear | Unclear |
| Laker (2007) [42] | | Harm reduction and motivational interviewing interventions | Unclear | Unclear | "MI was delivered by either psychologists with experience in the field of addictions or psychologists and nursing staff who received extra MI training for the purpose of the research". | "Psychologists with experience in the field of addictions, or psychologists and nursing staff who received extra MI training" | Unclear | Unclear | Unclear |
| **Domain 1 – Gambling** | | | | | | | | | |
| **"First author (year)"** | | ***NAME OF INTERVENTION*** | ***WHAT Materials were used and where accessed?*** | ***Procedures*** | ***WHO DELIVERED THE INTERVENTION?*** | ***DETAILS ABOUT THE INTERVENTION PROVIDER*** | ***HOW? Mode of delivery*** | ***WHERE: LOCATION*** | ***WHEN AND HOW MUCH?*** |
| Cowlishaw et al (2012) [43] | | Psychological therapies including MI | Handouts, worksheets, audio-recording equipment. | The patient was encouraged to make a decision about gambling and a change plan" Another study reported that "MI therapists employed the counselling skills from Miller and Rollnick (1991, 2002). The intervention was manualised to ensure that all participants were offered a similar experience. However, the therapists were free to follow the discussion as it occurred rather than force participants to follow the pre-determined format" Two RCTs used the same protocol with "therapists provided personalised feedback about participants’ gambling. They subsequently explored positive and negative consequences of gambling and discussed how gambling fit with their goals and values. Participants also completed a change plan worksheet. | "Interventions administered by psychologists, cognitive therapists, Masters level counsellors, or therapists with bachelors, masters or doctoral training. Some studies relied on mixed groups of clinical professionals or used doctoral students under supervision to deliver therapy." Two RCTs reported that therapists "received didactic training and close supervision of at least 1 case. Supervision consisted of reviews of therapy notes and audiotapes, and case discussion." | One RCT reported that the "therapists (1 licensed clinical psychologist with psychotherapist training and 20 years MI experience, 1 licensed clinical psychologist with 2 years clinical MI experience, and 2 licensed social workers, 1 of whom had 10 years’ experience and the other was newly trained in MI) supervised themselves as a group once a month through assessment of their own audiotaped sessions, using the Motivational Interviewing Treatment Code 2.0." | Face-to-face when delivering MI but follow-up assessment was done by telephone in one RCT. | "Studies were conducted in the U.S, Canada, Australia and Sweden. Nine of the 14 studies were conducted in outpatient settings. These included addiction or mental health treatment centres, university clinic, or more general community or research outpatient settings. The remaining studies did not fully describe the context of the trial." | Four of the 14 studies assessed MI. 2 trials considered motivational interviewing (or motivational interviewing counselling)." "Two of the 14 studies considered an integrative therapy (MET and condensed CBT, which involved a single session of motivational interviewing and personalised feedback about gambling (i.e.MET), and three optional sessions of condensed CBT". Length of interventions ranged from 10 minutes to 4 sessions with many of the sessions reported as lasting 50 minutes, and follow-up ranged from 3 months to 12 months. One study reported that , total therapist time per patient was 2.5 hours on average. The typical patient attended 2.94 (SD = 1.1) sessions. |
| Petry et al (2017) [44] | | “Motivational interventions  (MI) are designed to enhance engagement in treatment and  address ambivalence while guiding patients toward healthy behavioral  choices” | Unclear | Unclear | Unclear | Unclear | Unclear | USA and Sweden | 1 session of MI; 4 sessions over 4 weeks |
| Yakovenko et al 2015 [45] | | Motivational interviewing, or motivational enhancement therapy (MET) | Unclear | Motivational interviewing, or motivational enhancement therapy (MET) is a client entered  counselling approach that addresses the question of why certain individuals avoid change and persist in maladaptive behaviours (Miller & Rollnick, 2002). The goal of the therapist is to assess the client's readiness for change and to facilitate the transition toward behaviour modification by resolving any ambivalence about change. | Trained therapists Masters/Bachelors/PhD ; social workers & PhD level psychologists; doctoral students; PhD level psychologists | Unclear | Face to face x 6, phone x2 | Unclear | Unclear |
| **Domain 2 – Musculoskeletal problems** | | | | | | | | | |
| **"First author (year)"** | | ***NAME OF INTERVENTION*** | ***WHAT Materials were used and where accessed?*** | ***Procedures*** | ***WHO DELIVERED THE INTERVENTION?*** | ***DETAILS ABOUT THE INTERVENTION PROVIDER*** | ***HOW? Mode of delivery*** | ***WHERE: LOCATION*** | ***WHEN AND HOW MUCH?*** |
| Alperstein et al (2016) [46] | | MI aimed to improve individual’s readiness to change their behaviour by endorsement of a person-centred approach to resolve their ambivalence about behaviour change and strengthen their own motivation and commitment to change, to result in increased adherence to available treatment. |  | Each intervention included MI in addition to other exercise and educational interventions, 1 study included physiotherapy in combination with MI. 1 study include 2 hr guidelines training | Physiotherapist (2), Psychologists (2) Nurse (1), health Educator, pharmacist | ? | 1 study delivered MI via 6 telephone calls over 12 weeks, 1 as part of a physical therapy session, 1 included group sessions, Face to face in other studies. | Unclear | Ranged from 1 to 6 sessions. Session duration from 15-20mins to 1 1/2 hours. One study treatment duration delivered over a 12 week period. Others unclear |
| Chilton, et al (2012) [47] | | Trans theoretical model (TTM)-based motivational counselling approach. Motivational enhancement therapy (MET) |  | TTM based motivational counselling, MET; preparation for pain management; brief interventions, exercise-based motivational interviewing, Script assist telephonic counselling programmes | n= 1 practice nurses, n=1 registered practising psychologists ; n=1 third year doctoral student in clinical psychology; n=1 call centre nurses | Practice nurses were trained in general counselling skills (such as active listening, paraphrasing, verbal affirmation and reinforcement), TTM-based counselling and the MI style. Emphasis was placed on interactive exercises and role play, nurses received supportive material such fact sheet, wording suggestions, reminders and were provided with all written material used throughout the training. | Face to face, group and telephone | 1=Germany, others unclear | n=1; 10 sessions of 30 minutes in 8 weeks; n=1 brief (2 session) intervention of 1 -1.5 hrs. With no follow up n= 1; -3 sessions of 15-20 minute duration with follow up at 6-12 months; n=1 2 weekly 30 minutes each for 1-2 weeks. n=1; median of 3-5 telephone contacts depending on low or high risk. Average call duration was 15.3 mins. |
| **Domain 2 – Oral Health** | | | | | | | | | |
| **"First author (year)"** | | ***NAME OF INTERVENTION*** | ***WHAT Materials were used and where accessed?*** | ***Procedures*** | ***WHO DELIVERED THE INTERVENTION?*** | ***DETAILS ABOUT THE INTERVENTION PROVIDER*** | ***HOW? Mode of delivery*** | ***WHERE: LOCATION*** | ***WHEN AND HOW MUCH?*** |
| Cascaes et al (2014) [48] | | MI aimed at improving oral health | Unclear | Two phone feedback sessions targeting infant ETS reduction. Reduced measurement | "trained counsellor with extensive experience in MI" "two interventions were delivered by community members that received MI training, one was performed by two dentists and another two studies did not report who delivered the intervention sessions." | Unclear | "most studies received individualized MI intervention" Unclear if the video sessions were performed in groups or individualized in one study. In one study, MI sessions were conducted by telephone and in a separate study, follow-up sessions were also conducted by telephone" | "performed in high income countries in North America and Europe" | "MI interventions varied in duration (15 to 90 minutes) and number of sessions (one to seven), as well as by the addition or not of the traditional approach, as available, to the control group." |
| Gao,et al, (2014) [49] | | MI aimed at improving oral health | Workbook | Limited details, simply reported as MI. "In nine studies, MI was delivered in addition to CE (additive design). The ‘‘conventional education’’ often took the form of information advice given through printed materials, videos, and/or talks, whereas studies targeting oral hygiene for better periodontal health incorporated oral hygiene instruction or demonstration and some other elements, such as viewing of bacteria in plaque under microscope34 and reminder and telephone follow-ups." | "MI counsellors were dentists or dental hygienists (six studies), psychologists or social workers (four studies), community workers (three studies), researchers (two studies), or individuals with unknown background (one study"). | "In 15 of 16 studies, counsellors were trained on MI before delivering the intervention". Training ranged from 8-20 hours, with varying degrees of supervision. | Face-to-face, individual sessions and telephone follow up. | ? | Varied. Number of sessions ranged from a single session to 'multiple' sessions. In four studies, each participant joined more than one MI session, whereas in 11 studies, a single MI session was conducted. MI sessions lasted 5 to 90 minutes. Post-MI follow-up phone calls were made in four studies. |
| Kay et al (2016) [50] | | Motivational interviewing and other psychological interventions | Unclear | Unclear | Oral health professionals | Unclear | Mainly Individual face to face. Tailored to each individual | Very little information provided. Dental practice.  1 study = Periodontic clinic in Sweden | Varied. Little information provided. 1 study = 6 follow up sessions by phone. |
| Kopp et al (2017) [51] | | MI + periodontal therapy | Unclear | Lack of precise information about the MI-interventions in., The definition of Motivational  Interviewing changed over the years as did the style of MI can amongst the counsellors. | Dental students; MI trained counsellors; psychologist, dental hygienists | 2 studies evaluated  and controlled the application and quality of the MI elements  (Stenman et al., 2012; Woelber et al., 2016). E.g use of German version of the MITI 2.0. MITI values  MI was not applied in its full potential | Mainly Individual face to face. Tailored to each individual | Periodontics clinics  e.g. Germany | Varied; 1- 4  appointments of 15 -40 minutes |
| Werner et al (2016) [52] | | MI and other Behavioural interventions | Unclear | MI based on self-regulatory theory. Other RCTs used  components of the MI only | Dental hygienists (n = 4;  dentists (n = 2), a clinical psychologist (n = 1) a trained counsellor (n = 1), and unspecified. | Unclear | Face to face. | United States (n = 3), France (n = 1), Japan (n = 1), Belgium (n = 1), and Sweden  (n = 5), published between the years 1992 and 2014. Dental clinics. | Varied; 1 -2 sessions for 15-20 minutes. |
| **Domain 2: Eating disorders** | | | | | | | | | |
| **"First author (year)"** | | ***NAME OF INTERVENTION*** | ***WHAT Materials were used and where accessed?*** | ***Procedures*** | ***WHO DELIVERED THE INTERVENTION?*** | ***DETAILS ABOUT THE INTERVENTION PROVIDER*** | ***HOW? Mode of delivery*** | ***WHERE: LOCATION*** | ***WHEN AND HOW MUCH?*** |
| Macdonald, et al (2012) [53] | | MI is a ‘‘client-centred directive method for enhancing intrinsic motivation to change by exploring and resolving ambivalence’’ MET or adapted MI included (1)stand-alone motivational interventions,(2)motivational interventions combined with another intervention, e.g. CBT or (3)as one element of an intensive programme. Very brief interventions were also included. | 1 X self-help book, 1 x DVD, 1 x booklet on eating disorders | Behaviour change taxonomy devised to include discussion of; Elicit concerns about eating behaviours.  Exploration of ambivalence.  Discussion of trans-theoretical model of change and brief assessment of participant’s stage of change—assessment of motivation of readiness and confidence to change. Written decisional balance.  Bolster self-efficacy. Exploration of dissonance with values. Elicit ideas for possible behavioural alternatives to current ED behaviour. Work collaboratively on devising a change plan consisting of small, manageable steps. Complete‘ ‘Plans for Change’ ’worksheet. Interventionist empathy.  Use of reflective listening | 4 x clinical psychologists; 1 x doctoral students; 6 x health professionals; 2 x NR, 1 x undergraduate Masters student | 6 of 12 patient and carer participant studies provided details of interventionists having received M training but no further details provide | Face to face and group | In-patients settings; college, outpatient settings, day centres. | Varied 1 x 81 mins /16 x week; 4 x 1.25 hrs; 2 studies =4 x 60 minutes x16 weeks; 4 x 50 minutes; 4 x 50 minute; 12 +1 year and 2.5 year follow up; 5 day programme + 3 days follow up; 2 days/week for 5h/day for 6 months ; 6 x 2hr workshops; 3 x 40 mins -1 hr sessions |
| Knowles et al (2013) [54] | | MI for eating disorders | Self-help workbooks; Treasure and Schmidt’s manual | Adapted MI; MET; MI. Actual MI interventions varied in many ways across studies (e.g. single session to improve motivation to change and engagement to group sessions based on MET). However there is little detail provided about each intervention - just a mention in the introduction about types of MI. Unclear whether this is the actual approach used in each study. | Therapist. Limited details provided. | Unclear | Face-to-face; individual and group sessions. | Setting varied across studies: specialist outpatient (n=3); specialist inpatient (n=1); outpatient (n=1); inpatient (n=1); non-clinical sample (n=2). Geographical locations not stated. | Varied. MI sessions ranged from a single session (lasting 80-90 mins) to 4 sessions (duration not reported). Length of follow-up ranged from 2 weeks - 2.5 years. |
| Dray and Wade (2012) [55] | | Motivational interviewing with eating disorders | 6/9 studies MI manual, workbooks | Limited details, simply reported as MI, MET or adapted MI. (forms of MI used varied across studies, "including structured MI sessions with workbooks, motivational enhancement sessions using MI techniques, motivational assessment interviews, group MI and individual MI") | Unclear | Unclear | Face-to-face; individual and group sessions. Details not reported about individual studies. | Unclear | Limited details reported - not described for individual studies but referred to in the text ("number of MI sessions administered (ranging from 1 to 4 sessions), MI session lengths (ranging from 45 to 80 minutes per session") |
| **Domain 2: Weight loss management** | | | | | | | | | |
| **"First author (year)"** | | ***NAME OF INTERVENTION*** | ***WHAT Materials were used and where accessed?*** | ***Procedures*** | ***WHO DELIVERED THE INTERVENTION?*** | ***DETAILS ABOUT THE INTERVENTION PROVIDER*** | ***HOW? Mode of delivery*** | ***WHERE: LOCATION*** | ***WHEN AND HOW MUCH?*** |
| Armstrong (2011) et al [56] | | Motivational interviewing’ or if within the description of the intervention the authors indicated the use of methods developed by Miller W, Rollnick S. 2002. | Training mentioned in one study of dietician led MI intervention otherwise not described in paper | “Motivational interviewing was used in five studies as an adjunct to a behavioural weight-loss programme, which traditionally involves 16–24 treatment sessions over 6 months with a team of healthcare professionals. Individual studies included a variety of activities; single face to face counselling followed by 5 x15min sessions every 4 weeks,(Woollard 1995); adapted MI used by non-specialist; dietary counselling, 20 session behavioural weight-loss programme” | Professional background of the interventionists was variable and included nurses (n=2), psychologists (n=2), graduate students in psychology ( n=2), dieticians (n=2),health counsellors (n=2) and exercise scientists. (n=2) | No specific training mentioned | Varied from individual face-to-face to telephone and group sessions. Three studies  used face-to-face motivational interviewing at an initial consultation and provided follow-up via telephone. | Unclear | Follow-up duration range from 3 to 18 months. The dose ranged from 50 to 323 min. Motivational interviewing was used in 5 studies as an adjunct to a behavioural weight-loss programme. |
| Barnes et al (2015) [57] | | MI | Unclear | Unclear | Dieticians, nurses/nurse practitioners, medical doctors, or sports and health science specialists. exercise specialists, medical assistant and the remaining did not specify educational background (e.g. ‘trained non-specialists’) | Of the 12 (50%) studies reporting information about MI training most did not specify who provided the training; training provided in the remaining four studies was from MI ‘accredited’ or ‘certified’ trainers (n = 2), the study investigators (n = 1), and a MI Network of Trainers-certified trainer (n = 1) | Unclear | Unclear | Of the 12 studies reporting information about MI training length it is difficult to determine exactly how much training was received as almost half of these studies (n = 5) reported training in terms of ‘days’ or ‘evenings’ instead of hours. Of those that did report hours (n = 7, 29.2%) training length ranged from 3 (29) to 170 hours (48). Most studies did not describe any ongoing MI supervision for clinicians (n = 17, 70.8%). Of those that reported specific information regarding supervision (n = 7, 29.2%), the supervision provided ranged from weekly to every 6 months, most typically once every 3–4 weeks. |
| Hill et al (2013) [58] | | MI designed to limit Gestation Weight Gain | Patient educational materials | Only 2/21 studies used MI as a BCT. Intervention components included MI + patient educational materials, physical activity counselling + 1-2 supervised sessions/week, dietary counselling, weight gain advice. | Midwife trained in motivational lifestyle intervention in the RCT, and a specially trained midwife in the other study | Unclear | Unclear | "Six of the reviewed studies were conducted in the US, six in the Nordic countries, three in Canada, two in Brazil, two in Belgium, and one each in Australia and Taiwan." | 4-13 sessions provided. |
| VanWormer, and Boucher (2004) [59] | | Motivational learning  defined as a client-centred, directive method for enhancing intrinsic motivation to change by exploring and resolving ambivalence. | 1 study = educational manual; 1 study  referred to Miller and Rollnick’s book, Motivational Interviewing: Preparing People for Change, or to the MI Web site (www. motivationalinterview.org) | The following process was followed - Express Empathy; Develop Discrepancies; Roll with Resistance; Support Self-Efficacy. | 1 x nurse 1x clinical dietician | Unclear | Face to face; face to face and phone | 1 x American African. Others not stated but Ref list suggest USA. | Varied from 1 -6 face-to-face MI counselling sessions (45 min) including follow up. Treatment programmes lasted from 3 months to 1 year |
| **Domain 2: Management of metabolic disorders (diabetes)** | | | | | | | | | |
| **"First author (year)"** | | ***NAME OF INTERVENTION*** | ***WHAT Materials were used and where accessed?*** | ***Procedures*** | ***WHO DELIVERED THE INTERVENTION?*** | ***DETAILS ABOUT THE INTERVENTION PROVIDER*** | ***HOW? Mode of delivery*** | ***WHERE: LOCATION*** | ***WHEN AND HOW MUCH?*** |
| Mulimba, and Daniel (2014) [60] | | Motivational interviewing (MI) was initiated by Miller (1983) and later developed in collaboration with Rollnick (Miller and Rollnick, 1991). MI is highly adaptable | Unclear | Motivational enhancement therapy (a more systematic, time-limited, multi-session approach dubbed ‘motivational enhancement therapy’ (Miller et al, 1994). (MET) MET Plus cognitive behavioural therapy ( n=1) therefore study assessed addition of CBT. 42 sessions group weight management programme, diabetes education, 16 session weight-control group, patient education | n=1 MI provided by researchers with 12 hours training n smoking cessation. N=1 nurse educator, n=1 GPs, n=1 dietician, n=1 psychologists. Others not specified. | n=1 Researcher with 12 hours training, n=1 6 months training with supervision, n=1 2-day training in basic MI skills, n=1 MI delivered by psychologist experienced in MI, n= 1 supervision and training in MI skill coding. N=1 gaps trained 1.5 day training and 0.5 follow up. | Face to face and group | 2=UK, 4 = USA, 1 =Denmark, 1 =New Zealand, | 1 = 42 sessions with 6, 12 and 18 months follow up; 1 = 19 sessions with 4 months follow up. Follow up varied in other studies from no follow up to 3- 18 months. Only 3 studies followed up to 12 months |
| Ekong and Kavookjian (2016) [61] | | MI is a patient-centred communication  skills set aimed at evoking the intrinsic motivation of the  individual to develop the behaviour changes needed to manage T2D | Unclear | 8 reviews described MI training procedures and duration. Areas of focus specified for MI trainings included exploration of patient ambivalence, reflective listening, asking open-ended questions, and agenda-setting. Training period durations ranged from 10 to 80 h among the eight studies reporting training detail. 5 studies did not detail the training of interventionists, but simply stated that they were MI trained. One study did not include any references to training details or duration. | Trained medical professionals including one or  combined of general practitioner physicians (n = 2), psychologists  (n = 2), physician assistants (n = 1), nurses (n = 6), diabetes educators  (n = 1), dieticians (n = 1), and unspecified (n = 2). | Training durations ranged from 10 to  80 h among the eight studies reporting training details.  Five studies did not detail the training of interventionists, but simply stated that they were MI trained. 1 study did not include any references to  training details or duration. MITI, Motivational Interviewing Treatment Code  (MITC), and the Behaviour Change Counselling Index (BECCI) for intervention fidelity included in 3 studies. | Face-to-face (n = 11) or  group (n = 1) delivery of MI was applied for one or more MI  sessions (n = 3) included telephone follow-up session(s). The  14 studies had been carried out in a variety of outpatient settings  such as primary care clinics, doctor’s offices, and community  health facilities. | Unclear | Ranged from 30 to  90 min, with frequency of MI sessions ranging from one to  five  times |
| Jones et al (2014) [62] | | MI promoting healthy behaviour and removing barriers to positive change, to address ambivalence, to help individuals manage their diabetes more effectively | Unclear | Unclear | Unclear | Unclear | 1;1; group; video-phone | Netherlands (1); UK (4); Taiwan (1); USA (4); Denmark (2); W Indies (1); Locations: NR | Unclear |
| Lin et al (2014) [63] | | MI and other behaviour modification interventions | Additional educational reading materials x2 | Across the selected trials, the most commonly applied interventions were diet plans, supervised exercise, health education, individual psychosocial counselling including behavioural modification, self-management, and motivational interviewing. All of the interventions for managing MetS described in the selected trials were combined with a healthy diet and physical activity. | Nurses x3: nursing researchers, community  Nurses, or occupational health nurses. non-nurse-led (2) | Unclear | All interventions included face-to-face individual education or counselling and group education sessions. | Rural community (1); community (2); no other information reported | Duration ranged from 4 to 24 weeks. intervention duration of the LMPs varied from 12–15 minutes to 2 hours per session. |
| Soderlund [64] | | Motivational interviewing | Unclear | Various delivery methods but all counsellors used methods to guide conversations toward behaviour change. In  general, counsellors facilitate movement by  helping clients resolve ambivalent feelings  about behaviour change | Nurses and dental professional, health counsellors, trained teacher, GPs | Two of the four studies with significant PA  outcomes measured MI fidelity, and rated  counsellors as MI proficient. Most used some form of fidelity measure. E.g MITI to assess providers proficiency; research; 1 study used a a MI Network Trainer to train providers but MI sessions were not coded to ensure fidelity | Face-to-face sessions or a combination of face-to-face  and phone sessions. | USA, Canada, Europe,  Asia, and South Africa | e.g. MI dose (2), length (30 to 45 min); six MI sessions over 3 months; MI dose (4 to 6), intervals  (every 2 weeks),  length (45 to 60 min); sessions: follow up time varied from 2 months to 1 year |
| Thepwongsa et al (2016) [65] | | MI conducted as a single intervention or a major component of multifaceted interventions. | Unclear | One study included three workshops of different lengths, whilst another included an intensive training session, involving role-playing, rehearsals and a follow-up discussion after the first consultation with the patients | GPs -The majority of the  included studies did not state clearly the details of the training method,  but one provided multiple techniques | None of the  studies reported the effects of MI  training course on GP knowledge after the training program. | Face to face and phone | Primary care settings outpatient  clinics and general practices. 2 studies were conducted in Denmark), 1 in each of the US (52), Italy  , Belgium (and Bosnia and Herzegovina | Duration ranged from 30 minutes and 1 month follow up to 3 45 minute over 1 year. 30 minutes every 3 weeks. |
| **Domain 2: Management of neurovascular disorders (Stroke) and Cardiovascular disease** | | | | | | | | | |
| Cheng et al (2015) [66] | | Motivational interviewing | Unclear | Four key processes and involves the flexible and strategic use  of five core communication skills. The four processes seek to help  people resolve ambivalence through: 1) engaging them in a working relationship, 2) focusing on particular change, 3) evoking intrinsic motivations for change, and 4) planning a reasonable next step toward change (Miller 2002; Miller 2013). | Unclear | Unclear | Unclear | Unclear | One session per week for four individual sessions, with each session lasting for 30 to 60 minutes. |
| Hilderbrand ( 2015) [67] | | MI | Unclear | Unclear | Trained therapists -professional group not specified | Trained in MI by specialist and supervised by a clinical psychologist | Individual face- to-face | unclear | unclear |
| Lee et al (2016) [68] | | MI and MET. All interventions  utilized MI as the major component. | Telephone interviews | Unclear | Nurses, occupational therapist, exercise professionals, life coach, clinical psychologist, clinical nurse specialists. Registered Dietician, physical activity specialists | Six studies mentioned that MI  training was provided to the staffs who delivered the intervention, whereas three studies provided no information | Individual face-to-face sessions, some interventions included  both face-to-face and telephone follow ups. MET delivered during physical therapy session in 1 study; | Western countries, China and Hong Kong | Each face-to-face session lasted for 30–60 min, with a frequency of two to five times. Each telephone follow-ups lasted for 10–30 min, with a frequency of one to four times. |
| **Domain 2: Sexual Health Behaviour** | | | | | | | | | |
| **"First author (year)"** | | ***NAME OF INTERVENTION*** | ***WHAT Materials were used and where accessed?*** | ***Procedures*** | ***WHO DELIVERED THE INTERVENTION?*** | ***DETAILS ABOUT THE INTERVENTION PROVIDER*** | ***HOW? Mode of delivery*** | ***WHERE: LOCATION*** | ***WHEN AND HOW MUCH?*** |
| Berg et al 2011 [69] | | " MI or MET, used alone, as a prelude to other therapy or integrated with other therapy. To qualify as MI or MET for purposes of this review, the intervention had to be referenced with Miller and/or Rollnick and include a description of adherence to the four main principles of MI." | Various. Manuals, audio-recording equipment | Unclear | counsellors and peer-counsellors, clinical psychologists, psychiatrist, peer outreach workers, HIV nurses, psychology students and trainees | Master’s level students, Master's and doctoral level clinical psychologists. "All publications reported on providers’ training, which ranged from 4 to 60 hours of training, averaging approximately 27 hours". | Majority of studies used individual therapy; only one used group sessions to deliver MI. "Four of the interventions were delivered via telephone and the others individually face-to-face (one trial provided MI individually as well as in groups)". | All studies carried out in the USA, except one in the Netherlands | "The intervention “dose” ranged from one 30-minute MI session to 17 sessions of about 40 minutes, with an average of about 4 hours (250 minutes)." |
| Carrico et al 2017 [70] | | “Motivational interviewing (MI) is a client-centred, directive intervention targeting intrinsic motivation and self-efficacy for behaviour change that has been shown to be effective for reducing alcohol and substance use” | Unclear | Unclear | Unclear | Unclear | Face to face | Unclear | Varied ; e.g. 4 individual sessions |
| Dillard et al (2017) [71] | | “Motivational interviewing (MI) is a collaborative, conversation-  al form of counselling that focuses on strengthening patients’  motivation and commitment to change their behaviour” | Unclear | Unclear | Master's level-trained  counsellors; MINT  trainer, Mental health clinicians, Lay counsellors, clinical  psychologist, nurses, research  assistants, Psychologist | Master's level-trained  counsellors; MINT  trainer, Trained MI nurses, therapists,  Psychologist trained in  MI by MINT trainer | Individual sessions, group sessions, telephone sessions, and  combined modalities (face-to-face and telephone sessions). | Fourteen studies were conducted in the United  States]. Two studies were from  South Africa one from Nigeria and two from Thailand | Dose varied from 1 BMI session to 4 -5 60 minute sessions e.g. Four 60 min MI- based sessions (weeks 1, 2, 6, and 12 after baseline) with personalized feedback, plus SOC |
| Naar-King. et al (2012) [72] | | MI is a collaborative, goal-oriented method of communication to elicit and strengthen motivation for change. | 1 x video. 2X educational hand-outs; 1 x phone based MI | Unclear | 8 RCTs included MINT training. 1 x MI counsellors were advance degree clinicians with MINT training ; 1 x MI therapist with MINT training; 1 x advanced training but not specified | MINT training mentioned in RCTs http://www.motivationalinterviewing.org/ | face to face and group; telephone and video doctor via laptop and educational worksheet | Reference list suggests some studies carried out in USA but not mentioned in text/ African American mentioned in 1 study | Varied; 1 x 4 monthly 50 minute sessions; 1x 5 weekly 90 minute sessions followed by 3 monthly booster sessions; 3 x single sessions 1 with booster session at 3 months 1 lasting 90-120 minutes; 1 telephone session lasted on average 48 minutes. |
| Wilson et al (2015) [73] | | MI interventions that aimed to promote or inform contraceptive use | "motivational interviewing  Guide and training materials designed for intervention. . 1 intervention used CAMI computer-assisted motivational intervention. Information brochures ." | All MI with varied content/activities include: use of contraceptive journal, feedback, review goals, development & implementation of change plan, recording data on drinking, use of charts, referral to community health resources, informational brochures. | Trained counsellors: health educators; post graduate educated); locally recruited and trained lay counsellor. | Trained counsellors, training duration & content varied (30-40hrs; 2.5-day course, duration of others not specified). Trained by psychologist. Video-taped sessions, Weekly supervision or regular meetings. No details on training. | Face to face. One face to face followed by nine phone calls), booster by phone or to face to face, Enhanced home visit for over half participants. | USA=7, South Africa=1. Reproductive health clinic, primary healthcare facilities, universities, other diverse settings including women's jail, alcohol treatment centres. | Three studies offered single sessions, lasting between 60 and 75 min, one study offered two sessions including a booster session at two months, three studies delivered the intervention through 5-9 sessions (over varied time period 2mths to 2yrs), whereas another study initiated 1 face to face session with up to 9 follow-up phone calls |
| **Domain 2: Engagement with interventions** | | | | | | | | | |
| **“First author (year)”** | | ***NAME OF INTERVENTION*** | ***WHAT Materials were used and where accessed?*** | ***Procedures*** | ***WHO DELIVERED THE INTERVENTION?*** | ***DETAILS ABOUT THE INTERVENTION PROVIDER*** | ***HOW? Mode of delivery*** | ***WHERE: LOCATION*** | ***WHEN AND HOW MUCH?*** |
| Karmali et al (2014)[74] | | Interventions to improve uptake of and adherence to cardiac rehabilitation | **Unclear** | Varied and multifaceted e.g. supervised sessions; self-monitoring; goal setting; action planning; feedback; coping and problem-solving strategies; small group interaction; peer modelling; written & telephone communication; early appointment. | Cardiac rehabilitation staff, nurse, occupational therapist, social worker, researcher | **Unclear** | Varied: telephone call/visit ; combination of face to face & telephone; motivational in-hospital visit & telephone call from social worker; telephone coaching; motivational letters based on TBP; home visits; peer support group | Four studies were conducted in Canada, three studies were conducted in the UK, two studies were conducted in the US and one study was conducted in Germany. | Varied |
| Lawrence et al (2017) [75] | | MI | Motivation enhancement  treatment protocol; therapist manual; exposure and response and  motivational interviewing + MI  manual. Some interventions used audio/video assessment | Most studies described a script or protocol for the MI intervention. | Nurses, psychologist, PhD level clinical psychologist, graduate students, Nonspecific therapist. | Fidelity was absent in most of the studies. One study included 6 hr of didactic instruction,  shadowing, training cases; 1 study including training from 1^st^,  intensive workshop training, postworkshop  practices, relevant readings, 6 hr of didactic  instruction, role play with feedback | Face to face session, 1 with telephone follow up | Unclear | Varied; ranging from 1  to 2 phone calls for 15 min each (total 30 min) (Zanjani et al., 2008) to  three face-to-  face sessions totalling 6.5hr (Buckner & Schmidt, 2009) |
| Miller et al 2017 [76] | | MI and BMI | Telephone interviews, and screening Computer-assisted counselling telephone call. | Unclear | Unclear, health provider | 6 studies conducted fidelity monitoring | Telephone intervention, face-to-face or via computer. | USA, Black/African American0. Native American4.0Asian/Pacific Islander, Non-Hispanic | Dose of the interventions ranged from a one-time 6-minute phone call to a multi session intervention |
| **Domain 2 - Adherence to medication interventions** | | | | | | | | | |
| Ali Hussein Al-Ganmi (2016) [77] | | MI Counselling based on Social Cognitive Theory and MI | Unclear | 1 study included Motivational interviewing (MINT) for improving medication adherence | Nurses | Unclear | Face-to-face | China, Community health centres (1 hospital-based practice; 1 community-based practice); Spain and USA | MINT group in 1 study received behavioural counselling about medication adherence for 30-40 minutes at three, six, nine, and 12 months |
| Binford, et al 2012 [78] | | Motivational Interviewing (no further description) | Unclear | MI and cognitive-behavioural skills building (8 sessions over 8–12 weeks). Pilot studies included MI+ feedback and MI +CBT. | Unclear | No specific training mentioned | individual face to face | Unclear | 1 RCT = 8 sessions over 8-12 weeks, 2 pilot studies not reported |
| Easthall, et al 2013 [79] | | "Behaviour change techniques as interventions to improve medication adherence" | Self-help workbook, Software-supported intervention (based on transtheoretical model of change and MI), educational DVDs, telephone | "MI was used in 11 studies (42.3%). Variety of procedures: Comprehensive programme integrating a skill-orientated self-help workbook with one to-one counselling and adherence-enhancing strategies; Software-supported intervention based on transtheoretical model of change and MI, individual counselling sessions, "Adherence intervention guided by the Information-Motivation-Behavioural Skills (IMB) model | "routine healthcare providers such as nurses, pharmacists and general medical practitioners. ‘Non-routine’ healthcare providers were considered to be those such as psychologists or psychotherapists, who would not ordinarily be involved in the patient’s care." | Unclear | Face-to-face, individual counselling reported in the majority of studies. Telephone delivery of MI also used in some studies. | "primarily undertaken in the USA, followed by the UK, Australia, and the Netherlands. Interventions were delivered in community-based clinics, hospital clinics, home visits or by telephone. | Intervention period ranged from four (15·4%) studies reporting singular sessions, to six (23·1%) studies reporting multiple sessions over 12 months. interventions were delivered over a period of 6 months or less, which was the case for 17 studies (65.4%). |
| Hill, et al 2012 [80] | | MI is defined by its patient-centred approach and the three traits of its ‘‘spirit’’: collaboration, evocation, and patient autonomy. |  | Three of the five studies used MI sessions in person, but one study also utilized phone calls for some MI sessions, and another study chose to include behavioural audiotapes, a workbook, and a mailed booster with the MI sessions. |  | Three of the five studies trained providers specifically for the study, while the remaining two used practitioners previously trained in MI. Provider training length varied from 24 hours to 5 days. | face to face and telephone follow up | 4 USA, 1 France | varied from 2-6 months in duration including 2-8 session; follow up varied from 1 month to 10 months |
| Hu et al (2014) [81] | | Motivational interviewing | motivational letters; leaflets, pamphlets | Unclear | Nurses | Unclear | Unclear | African-American population. Location unclear | Unclear |
| Nieuwlaat, et al (2008) [82] | | Medication review including adherence intervention targeting more than one medication, while 27 targeted one medication (the number of medications was unknown for the remaining 15RCTs | 1 study used a MI script to guide the interaction with the participants. 1 used a resource of tools for coaches to facilitate motivational interviewing | Varied | Allied Health Professionals such as pharmacists; counsellors, nurse, trained GPs | One study trained GPs. The intervention group courses consisted of a 1½ -day training sessions with a half day follow-up twice during the first year. | Face to face; group, telephone | 44 from USA, 17 from middle-income countries, five from low-income countries, and 7 + from unknown | Varied |
| Palacio et al ( 2016) [83] | | The methods specifically  described MI or the MI principles as the main strategy of  counselling. | 3 studies provided additional materials. These included video or audiotapes, educational materials, calendars or journals. | Most studies included an adequate description of MI  and the intervention procedures. The authors reported using  known MI strategies and many described topics for specific  sessions e.g. 6 MI sessions plus feedback and skills building over 8 weeks. | Nurses, clinical psychologist, clinical nurse specialists, Health workers, psychology  doctoral students | Training of providers reported in 10 studies.  Ranged from 4 to 40 h. Most studies reported approximately  24 h of training. 9 of the 17  studies used a fidelity assessment tool | Telephonic,  in-person individual, group or mixed delivery. | 14 studies conducted in the USA. 13  in outpatient clinics most commonly in large metropolitan areas | The intervention lasted 3 to 12 months; ranged from 1 week apart to 12 weeks. 11studies ranged from 30 min to 2 h (for group MI). Most of the studies described MI lasting 45 to 60 min. |
| Rueda et al (2006) [84] | | "patient support and education to improve adherence to HAART " | "interventions that focused on medication management skills consistently used reminders or memory aids, such as beepers, alarms, medication boxes, planning cards, paging systems, text messaging, or visual aids." RCTs specifically reporting on MI used "motivational materials (e.g., videotape, journal, calendar)" Another RCT used a "video-taped presentation" | The interventions included all types of patient education, counselling, support, health promotion, reminders, provision of resources, supervision, consultation, and telephone hotlines. | Interventions were delivered by lay individuals, health advocates, social workers, psychologists, nurses, pharmacists, and physicians." | Limited. MI specific RCTs report "trained and supervised nurses" or "trained and supervised therapists". Another RCT referred to a "Nurse trained in motivational interviewing" | "All interventions were directed at patients, individually or in groups, rather than at providers or health care systems." | "Twelve studies were conducted in the USA, two in Spain, two in France, two in Australia, and one in Switzerland." | "Length of follow-up ranged from 6 weeks to 15 months.", "The duration of the interventions ranged from a single session to multiple sessions delivered over one year." |
| Zomahoun et al (2017) [85] | | The study authors  had to explicitly describe MI with reference to the  work of its developers, Miller and Rollnick | Varied for each study including feedback | Varied for each study including feedback | Nurses, other health professionals | MI fidelity assessed in 10 studies | Face to face; phone calls, phone call and fance to face combined, group, computer based | Mainly USA, Australia= 1, 1 + china | Varied from 1 to 8 sessions; 20-120 minutes. Duration of intervention ranged from 1 -12 months |
| **Domain 2: Management of patients and survivors of cancer** | | | | | | | | | |
| Spencer & Wheeler (2016) [86] | | MI interventions to address lifestyle behaviours; psychosocial  outcomes, and cancer-related symptom  management. | 5 studies incorporated  diaries or worksheets as part of their MI interventions to further  reinforce the behaviour change | Unclear | MI-trained nurse or dietician  MI-trained, non-nurse counsellors 1 study trained cancer survivors to act as MI counsellors. Training ranged from several days to several months. | 8 studies included some form of review process to ensure the fidelity of the MI protocol. Different methods of evaluation were used ; In 2studies, a certified trainer from the MINT network assessed a randomly selected sample of calls In others, a sample of sessions was assessed by a study member, using (MITI) Two studies held monthly meetings to reemphasize the  principles of MI and discuss previous counselling sessions | 3 studies sessions  Face to face and 7 studies over phone | USA otherwise Unknown clinical setting | Varied; Six studies recruited cancer survivors after treatment e.g. 5 MI calls in 6 months; 3 calls in 3 months, 22 calls over 12 months. |
| **Domain 2: Management of patients with irritable bowel disorder (IBD)** | | | | | | | | | |
| Wagonera, and Kavookjian (2017) [87] | | MI | Unclear | Unclear | Physician (n = 2), a  registered nurse (n  = 1), and MSc level  therapist (n = 1). | One described the interventionist  as experienced and qualified in counselling and psychotherapy. an 8-h program on the study’s specific counselling intervention  coupled with individual supervision and weekly team meetings  with research personnel | Face to face and telephone calls | Unclear | Varied and included: telephone calls over a 6-month period averaging 13 min each, one 45-min routine consultation, one 40- min MI session followed by five 40-min mindfulness-based sessions occurring over 16 weeks, and one 20- to 30-min MI session. |
| **Domain 3: Reviews focused on multiple health related problems and /or multiple health behaviours** | | | | | | | | | |
| **"First author (year)"** | | ***NAME OF INTERVENTION*** | ***WHAT Materials were used and where accessed?*** | ***Procedures*** | ***WHO DELIVERED THE INTERVENTION?*** | ***DETAILS ABOUT THE INTERVENTION PROVIDER*** | ***HOW? Mode of delivery*** | ***WHERE: LOCATION*** | ***WHEN AND HOW MUCH?*** |
| Burke et al 2003 [88] | | Adapted MI (AMI) | Unclear | AMI categorised as :prelude to further treatment = 9; stand -alone treatment = 17; adjunct to standard treatment | training procedures were not carefully described or standardized (e.g., guided by a manual), and integrity checks to measure the implementation of treatment—including videotaping, ongoing supervision, and coding of actual therapist behaviours— were entirely absent from several studies | No specific details given about provider other than mention of training standardised in Miller Clinics. "studies conducted in the clinic of the founder of MI (W. R. Miller) produced higher effect sizes, on average, than studies conducted elsewhere. | Individual (not group) and face-to-face | Not in text- reference list suggest USA and UK | Dose ranged from 15 to 240 min with a mean of 99 total treatment min. The comparative dose of the AMIs (minutes of AMI minus minutes of comparison treatment) ranged from –480 to 270 with a mean of 22 comparative min. Follow-up length ranged from 4 weeks to 4 years, with a mean of 18 weeks, whereas rates of follow-up ranged from 26% to 98%, with a mean of 74%. |
| Dunn et al 2001 [89] | | MI method defined by Rollnick & Miller (1995) as a directive, client-cantered style of counselling that helps clients to explore and resolve their ambivalence about changing. | No technical support used | no further information other than a) claiming to utilize the principles and techniques of MI and also reporting some form of monitoring of MI sessions and measuring behavioural and/or health outcomes rather than only knowledge or attitudes. | 17 studies = PhD psychologist or doctoral student; 4 studies = specialist substance abuse clinician; 6 =college degree or undergraduate student; 7 = health counsellor, nurse or dietician | Of 29 studies, 10 reported the number of hours of training provided to MI interventionists, which ranged from 2 hours to 31 hours (Booth et al., 1998) and averaging 15 hours. Eleven studies reported providing training but did not specify the number of hours, and eight failed to mention anything about training. | Face to face individual or in groups (Specifically excluded computer or telephone) | Not in text- reference list suggest USA settings included 5 specialist substance abuse treatment agency; 5 university campus; 5 hospital (inpatient); 5 outpatient medical clinic; 5 emergency room; 5 outpatient community agency. | Dose varied between 5 to 360 minutes. In the nine studies comparing MI to a no treatment control group, average duration = 104 minutes. In 14 studies comparing MI to a comparison treatment, average = 98 minutes,  the average duration of the comparison treatments was 190 minutes. In seven studies testing MI as an enhancement to usual treatment, the average duration = 70 mean duration of usual treatment ranged from 20 hours to 28 days. |
| Hettema, et al 2005 [90] | | MI across multiple target problems | Of the 72 studies included in the analyses, most (74%) reported that the MI  intervention had been standardized by a manual or a specific training. | Interventions were  specified as including the following components of MI: being collaborative, being  client centred, being non-judgmental, building trust, reducing resistance, increasing readiness to change, increasing self-efficacy, increasing perceived discrepancy, engaging in reflective listening, eliciting change talk, exploring ambivalence, and listening empathically. The total number of these strategies reported to have been  implemented in interventions identified as MI ranged from 0 to 12 (mean = 3.6,  SD = 2.8). | The agents implementing the MI, when specified, included paraprofessionals or students (8), master’s level counsellors (6), psychologists (6), nurses (3),  physicians (2), dieticians (1), and modally a mix of varying levels of professionals  (22). | For 13  studies that reported amount of training time, a mean of 9.92 (SD = 7.35) hours  was spent in training. Only 26 studies (29%) provided any kind of post training  support (such as supervision) for therapists, and only 21 studies (36%) included  any form of competency or fidelity assessment after initial training. | Unclear | MI was delivered in a variety of settings, including aftercare or outpatient clinics, inpatient facilities, educational settings, community organizations, general practitioner offices, prenatal clinics, emergency rooms, employee assistance programs, halfway houses), over the telephone, in  patients’ homes, in jail, in mixed settings, or in unspecified treatment settings. | “The duration of MI interventions varied. In 68 studies that reported these  data, MI duration ranged from 15 minutes to 12 hours, with an average dose of about two sessions (mean = 2.24 hours, SD = 2.15)”. |
| Lundahl et al (2010) [91] | | MI | Unclear | Feedback was provided from standardized assessment instruments in MI style (i.e., MET) in 21 studies, whereas 30 studies delivered basic MI without problem feedback" | Medical doctor;  registered nurse or registered dietician; mental health provider with either a master’s degree or a PhD; mental health counsellor with a bachelor’s degree; or (e) student status, which generally indicates that the student was being supervised by someone with a Master’s or PhD degree. | Unclear | Unclear | Unclear | Unclear |
| Martins and McNeil, (2009) [92] | | MI defined as "directive psychosocial intervention used to identify and resolve discrepancies between desired behaviours and actual behaviours, and to increase motivation to facilitate behaviour change (Miller & Rollnick, 2002)". Motivational Enhancement Therapy | 1 study included online MI (2003). Motivational Interviewing Skill Code (MISC), Version 2.0 <http://www.motivationalinterviewing.org> Motivational Interviewing Skill Code (MISC), Version 2.1 Retrieved December 2, 2008, from http://casaa.unm.edu/ download/misc.pdf | Varied e.g. MI was include in all interventions in combination with healthy eating video/printed educational material, health and fitness guide, weekly group behavioural sessions | n=1 health care professional, n =1 dietician, otherwise not well documented. | Unclear | Face to face, internet online sessions, group, telephone | Reference list suggests that studies were carried out in USA and UK | Dose of MI varied across 37 studies; e.g. Monthly over 6 months; 4-30 minute session, 11 face-to-face with mean of 34 minutes contact. Brief intervention for 30 minutes with weekly follow up, |
| O'Halloran. et al (2014) [93] | | MI defined as "defined as a person-centred directive counselling style used to address individual ambivalence about behaviour change through placing the emphasis on clients producing their own argument for change" Relies on the three core characteristics of motivational interviewing3 delivered individually with personal contact (i.e. phone or in person). | Accelerometer, ergometer, physical activity logs used as outcomes and feedback. Telephone feedback | Varied; 1 = MI + supervised exercise; 2 =MI + weight loss programme; 4= MI alone; 1=MI + written material | Counsellors x3; MI trained Health practitioners X1; Advanced clinical doctoral student X2; trained physiotherapists x1; trained psychologist x 1; unclear x 1, Physical activity specialist or registered dietician x1 | Varied e.g. Training and supervision by doctoral level clinical psychologist. Read textbook on MI; watched training videos, 2 day training session, and simulated counselling sessions; 2 day training course and additional training and supervision from a clinical psychologist experienced in MI; programme in MI and additional training and supervision from a clinical psychologist experienced in MI; 20 hours of readings, video, role play and discussions of MI principles and strategies. | telephone delivery x7; face to face x 8 | Primary care and community care - 6 RCTs =USA, 2 RCTs = UK, 1 RCT =Australia and 1 + Canada | Mean sessions attended 2 x 20-30 minutes (1.58) over 6 /12 month 6 x 24 minute sessions over 12 weeks; 4 X 30-minute sessions over 13 weeks; 60-90 mins face to face; 117.1 (56.6) minutes telephone over 12 weeks; Average 5.4 (45-60 minutes) sessions); received at least 3 sessions. 79%, 6 or more sessions; Single session 30-45 minutes over 12 weeks; Adherence between 83-100% over 52 weeks. |
| Rubak et al (2005) [94] | | Motivational interviewing as defined according to Miller and Rollnick. | Unclear | Unclear | Psychologists (55% [42/76]); medical doctors (30% [23/76]); and others such as nurses, midwives and dieticians (15% [1/76]. | Unclear | individual ( face to face ) group and telephone | No details in text. Reference list suggest USA 1 =UK | Individual interview, 10–20 n = 11; 30–45 n=17; 60 n= 32;  60–120 n= 6;  Group interview n= 3;  Telephone interview n =1. varied <3; 3–5; 6–11;  12–24; >24 |
| Shingleton et al (2017) [95] | | MI/AMIs delivered through electronic media. the term technology assisted motivational interview (“TAMI”) is used to define adaptations of MI delivered via technology and various types of media | Computer, video, mobile phone, animation, telephone | The technologies used to deliver the TAMI varied in terms of both degree of expert interaction (i.e., synchronous, asynchronous, or no communication) and level of media richness (e.g., Text-only Audio files, Video) | Therapist or unspecified | Only 2 studies reported MI treatment fidelity data. E.g. independent coders rate transcripts from Internet chat-therapy sessions. | Varied levels of therapist and client interaction via technology, with n = 9 using expert-guided protocols and n = 32 delivering fully-automated TAMIs (i.e., no therapist/expert interaction). No face to face. | n = 28 studies delivered the TAMI in a hospital, clinic/community centre, school, or university lab  n = 13 conducted completely online without any form of FTF interactions | Unclear |
| Thompson et al.2011 [96] | | Motivational interviewing in relation to cardiovascular health. | "Exercise and nutrition leaflet" | Unclear | Specialist nurse delivered the intervention in one study; details not reported in the remaining 4 primary studies. | Very little detail (e.g. nurse trained in motivational interviewing). | Face-to-face but often unclear how MI delivered in the 5 empirical studies | USA studies home visits; UK hospital visits (ward and clinic) | Ranged from 3 -5 sessions to "eight times per hour" sessions. Sessions length also varied across studies from 20-30 mins to average of 1 hour 42 minutes. |
| **Domain 4: Reviews focused on behaviour change interventions in specific settings** | | | | | | | | | |
| **"First author (year)"** | | ***NAME OF INTERVENTION*** | ***WHAT Materials were used and where accessed?*** | ***Procedures*** | ***WHO DELIVERED THE INTERVENTION?*** | ***DETAILS ABOUT THE INTERVENTION PROVIDER*** | ***HOW? Mode of delivery*** | ***WHERE: LOCATION*** | ***WHEN AND HOW MUCH?*** |
| Kohler et al (2015) [97] | | Brief motivational  intervention | Written information, contact list, education brochure.  Supplementary material is available at Alcohol and Alcoholism online | MI interventions included a treatment more or less similar to the control intervention plus MI. One trial combined MI with normative resetting and skills training. In two trials, the MI groups also received additional ‘booster’ phone calls after 10 days, or after 1 and 3 months that were not part of the control interventions. | Psychologist named in 1 trial | Unclear | Face to face, booster phone call in 2 trials | USA x 5, Brazil x1. Paediatric Emergency department (D) x1, ED or scheduled return visit to hospital if unable to complete during ED visit | Duration was 5 to 45 min and usually longer than 20 min, or had a median length of 37 min. |
| Knight et al (2006) [98] | | MI in physical healthcare settings | Unclear | "6/8 of studies included a detailed explanation of the main components and principles of MI, detailing use of reflective listening, rolling with resistance, developing discrepancy". | Intervention therapists; experience and certified therapists | "two non-RCT described the length and type of training in MI that intervention therapists received, ranging from a combination of workshops, training videos, role play and individual supervision spread over 3 months to 15-20 hours of didactic teach practice, and reading supervised by a certified trainer". | Unclear | Unclear | Unclear |
| Lundahl, et al (2013) [99] | | MI in medical care settings | Unclear | "Feedback was provided from standardized assessment instruments in MI style (i.e., MET) in 21 studies, whereas 30 studies delivered basic MI without problem feedback" | "mental health professionals (13 studies), nurses (6 studies), dieticians, physicians, or mixed provider types" | "Providers spent an average of 18 h learning MI, though there was a wide range (4–40)." | Face-to-face and phone. Data not extracted for individual vs group. | MI was used in a variety of medical locations including home, hospital, primary care, community settings, cancer network, dental practice, HIV clinic - see table 2 for complete list). | "Average time patients received MI was 106 min, longer than the 30 min interventions for comparison groups. The mean number of sessions dedicated to delivering MI in a face-to-face interaction was 2.6 (or 3.0 sessions of phone MI). |
| Merz et al (2015) [100] | | Motivational interviewing | education brochure; manual to guide MI | MI + phone booster at 1 & 3 months; MI + education brochure + 5 min discussion; manual guided MI | 2 x trained staff within ER, 1 x trained psychologist, 1 x nurse | All studies involved therapists specifically trained to deliver the intervention. 3 x regular supervision | Individual face to face x3; face to face & phone follow up x1 | USA=2, UK=1, Brazil=1. 3 in emergency room; 1 in surgical clinic 10 days post injury | 35-40 min MI sessions with telephone boosters at 1 & 3 for some studies. |
| Noordman et al 2012 [101] | | "Face-to-face communication-related behavior change techniques (BCTs)" including MI | Telephone; verbal and written advice; pedometer. | Procedures described here relate only to 9 studies that explicitly reported MI in the intervention. Procedures included brief verbal and written advice including behaviour change advice based on goal setting, self-monitoring and MI from an ES; green prescription programme in 2 studies. | GPs, Physicians assistants, nurses (public health nurse, nurse practitioners, practice nurses, district nurses, health visitors, nurse health counsellors), health educators, exercise scientists, nutritionists, specialists in endocrinology, and internal medicine, dietician, physiotherapist, respiratory experts and lung function technicians. | Not specifically reported. One study mentions "GPs received a motivational interviewing (MI) course" | Face-to-face; telephone. Not clear if individual or groups. | USA, UK, Australia, Netherlands, Denmark, Spain, NZ, Finland, Italy, Canada, Thailand, Taiwan, Norway and Sweden. | "face-to-face communication between patient and health care provider ranged from (seven studies with) one session to (one study with) 15 sessions. The number of face-to-face sessions does not include separate measurement sessions of biomedical or questionnaire data (only if part of the face-to-face BCT), telephone calls or written advice that were sometimes also part of the interventions. In three studies the frequency of the face-to-face communication sessions was not reported. The face-to-face communication sessions lasted from 30 s to 60 min. Fourteen studies did not report the duration of the face-to-face sessions" |
| Purath,et al (2014) [102] | | MI Defined as "a communication method that employs client centred counselling to elicit and strengthen motivation for behaviour change" (Rollnick, Miller, and Butler described an expansion of its use in addressing a wide array of health behaviour changes needed to combat and manage chronic disease) | newsletters and educational information; telephone calls; email communication; videophone calls; telephone calls based on Trans theoretical Model.  [http://www.motivationalinterview.org](http://www.motivationalinterview.org/) | 1 x MI +received quarterly newsletters and invited to attend monthly events.; 1 x tailored MI +Tailored print communication; 1 x video-phone intervention; 1 intervention integrated Transtheoretical Model, flexible scripts and MI techniques; 1 x MI health education; 1 x home-based, unsupervised, information with motivational telephone calls based on Transtheoretical Model; 2 x MI only | 5 nurses; 2 psychologists; 1 research team member; 1 counsellor; 1 unknown | 1 20 hours training session ;1 x trained by person experienced in delivering MI; others not described | face to face; telephone delivery; video calls. | 1 USA, 1 UK others unknown | 1 Initial visit followed with monthly phone calls 10- 15 min each for 6 months; 1 8 weekly home-based sessions;1 MI calls delivered over a 9-month period. MI-only group received 3 bimonthly calls for the first 6 months (months 2, 4, and 6) and one call at 9 months post-baseline; Six-month video-phone intervention. 15-min weekly videophone calls to experimental participants for 3 months followed by 15-min monthly calls for an additional 3 months. |
| Taggart et al (2012) [103] | | “MI used in 1 of 6 Interventions that had to include at least one measure of health literacy and promote positive change in lifestyle behaviours for smoking, nutrition, alcohol, physical activity and/or weight. | 1 x video and written material  Unclear | Unclear | Nurse; physician and registrar | Unclear | Face to face | Primary health care (n = 28), the community (n = 20) or other settings (n = 4) such as hospital outpatients clinic or worksite. Studies were from the US (n = 30), Australia/ New Zealand (n = 4) and other OECD countries (n = 18). NOT ALL MI | Varied 1-3 session for most; 1 study = 12-20 week individual counselling |
| VanBuskirk, and Wetherell (2014) [104] | | MI takes a patient-centred approach that empowers the patient to develop his or her own motivation and creates a therapeutic alliance that is predominantly a partnership, rather than an expert/patient dynamic (Rollnick & Miller, 1995). | Pamphlets and telephone calls | All interventions included 1 or more MI sessions, Seven of the 12 studies used telephone calls in the delivery of the intervention. Five of these studies used phone calls as “booster” or follow-up sessions; however, two studies used the telephone as the only mode of delivery of the intervention | 2 Nurses; 1 trained case manager; 1 health promotion counsellor; 1 physical Activity specialist or registered dietician; 1 master's level student; 2 trained research assistant or interventionist; 2 physicians | Unclear | Face to face; 5 telephone sessions, | 9 in USA; 2 in UK. 1 in Spain; primary care settings in rural communities, universities, urban setting in Spain, 3 low -income communities. | Five of the reviewed = 1 session; 8 of the 12 reviews used 3 or fewer sessions.1x 45-60 minutes; 1 15-20 minute; 2 30-45 minute and 4 follow up; 1 5 20-30 minute sessions; 1 x 20 minutes |

1. Baxi R, Sharma M, Roseby R, Polnay A, Priest N, Waters E, et al. Family and carer smoking control programmes for reducing children's exposure to environmental tobacco smoke. Cochrane Database of Systematic Reviews [Internet]. 2014; (3). Available from: <http://onlinelibrary.wiley.com/doi/10.1002/14651858.CD001746.pub3/abstract>; <http://onlinelibrary.wiley.com/store/10.1002/14651858.CD001746.pub3/asset/CD001746.pdf?v=1&t=ioifgsw9&s=e90df27c627ac8d2192ead2aae3ad2b04b3e26b3>.

2. Baxter S, Blank L, Everson-Hock ES, Burrows J, Messina J, Guillaume L, et al. The effectiveness of interventions to establish smoke-free homes in pregnancy and in the neonatal period: a systematic review. Health education research. 2011;26(2):265-82. doi: 10.1093/her/cyq092. PubMed PMID: 21273185.

3. Behbod B, Sharma M, Baxi R, Roseby R, Webster P. Family and carer smoking control programmes for reducing children's exposure to environmental tobacco smoke. The Cochrane database of systematic reviews. 2018;1:CD001746. doi: <https://dx.doi.org/10.1002/14651858.CD001746.pub4>.

4. Ebbert JO, Elrashidi MY, Stead LF. Interventions for smokeless tobacco use cessation. The Cochrane database of systematic reviews. 2015;10:CD004306.

5. Heckman CJ, Egleston BL, Hofmann MT. Efficacy of motivational interviewing for smoking cessation: a systematic review and meta-analysis. Tob Control. 2010;19(5):410-6. doi: 10.1136/tc.2009.033175. PubMed PMID: 20675688; PubMed Central PMCID: PMC2947553.

6. Hettema JE, Hendricks PS. Motivational interviewing for smoking cessation: a meta-analytic review (Structured abstract). Journal of Consulting and Clinical Psychology [Internet]. 2010; 78(6):[868-84 pp.]. Available from: <http://onlinelibrary.wiley.com/o/cochrane/cldare/articles/DARE-12011000298/frame.html>.

7. Lindson-Hawley N, Thompson TP, Begh R. Motivational interviewing for smoking cessation. Cochrane Database Syst Rev. 2015;3:CD006936. doi: 10.1002/14651858.CD006936.pub3. PubMed PMID: 25726920.

8. Mantler T, Irwin JD, Morrow D. Motivational interviewing and smoking behaviors: a critical appraisal and literature review of selected cessation initiatives. Psychological reports. 2012;110(2):445-60. doi: 10.2466/02.06.13.18.PR0.110.2.445-460. PubMed PMID: 22662398.

9. Pelletier JH, Strout TD, Baumann MR. A systematic review of smoking cessation interventions in the emergency setting. Am J Emerg Med. 2014;32(7):713-24. doi: 10.1016/j.ajem.2014.03.042. PubMed PMID: 24768666.

10. Rabe GL, Wellmann J, Bagos P, Busch MA, Hense H-W, Spies C, et al. Efficacy of emergency department–initiated tobacco control—Systematic review and meta-analysis of randomized controlled trials. Nicotine & Tobacco Research. 2013;15(3):643-55. PubMed PMID: 2013-09864-004.

11. Stead Lindsay F, Koilpillai P, Fanshawe Thomas R, Lancaster T. Combined pharmacotherapy and behavioural interventions for smoking cessation. Cochrane Database of Systematic Reviews [Internet]. 2016; (3). Available from: <http://onlinelibrary.wiley.com/doi/10.1002/14651858.CD008286.pub3/abstract>; <http://onlinelibrary.wiley.com/store/10.1002/14651858.CD008286.pub3/asset/CD008286.pdf?v=1&t=ioif8zin&s=0efea3419f5d11120bf2df4c7fc3bb44257b7a83>.

12. Appiah-Brempong E, Okyere P, Owusu-Addo E, Cross R. Motivational interviewing interventions and alcohol abuse among college students: a systematic review (Provisional abstract). Database of Abstracts of Reviews of Effects [Internet]. 2014; (2):[e32-e42 pp.]. Available from: <http://onlinelibrary.wiley.com/o/cochrane/cldare/articles/DARE-12014024502/frame.html>.

13. Barrio P, Gual A. Patient-centered care interventions for the management of alcohol use disorders: a systematic review of randomized controlled trials. Patient preference and adherence. 2016;10:1823-45.

14. Branscum P, Sharma M. A review of motivational interviewing-based interventions targeting problematic drinking among college students. Alcoholism Treatment Quarterly. 2010;28(1):63-77. PubMed PMID: 2010-07481-006.

15. Carey KB, Scott-Sheldon LA, Carey MP, DeMartini KS. Individual-level interventions to reduce college student drinking: a meta-analytic review. Addict Behav. 2007;32(11):2469-94. doi: 10.1016/j.addbeh.2007.05.004. PubMed PMID: 17590277; PubMed Central PMCID: PMC2144910.

16. Carey KB, Scott-Sheldon LA, Elliott JC, Garey L, Carey MP. Face-to-face versus computer-delivered alcohol interventions for college drinkers: a meta-analytic review, 1998 to 2010 (Structured abstract). Clinical Psychology Review [Internet]. 2012; 32(8):[690-703 pp.]. Available from: <http://onlinelibrary.wiley.com/o/cochrane/cldare/articles/DARE-12012049936/frame.html>.

17. Chatters R, Cooper K, Day E, Knight M, Lagundoye O, Wong R, et al. Psychological and psychosocial interventions for cannabis cessation in adults: A systematic review. Addiction Research & Theory. 2016;24(2):93-110. doi: 10.3109/16066359.2015.1073719. PubMed PMID: 112337071. Language: English. Entry Date: 20160122. Revision Date: 20180516. Publication Type: Article.

18. Cooper K, Chatters R, Kaltenthaler E, Wong R. Psychological and psychosocial interventions for cannabis cessation in adults: a systematic review short report. Health Technol Assess. 2015;19(56):1-130. doi: 10.3310/hta19560. PubMed PMID: 26202542; PubMed Central PMCID: PMCPMC4781488.

19. Darker CD, Sweeney BP, Barry JM, Farrell MF, Donnelly-Swift E. Psychosocial interventions for benzodiazepine harmful use, abuse or dependence. The Cochrane database of systematic reviews. 2015;5:CD009652.

20. Gates P, J, Sabioni P, Copeland J, Le Foll B, Gowing L. Psychosocial interventions for cannabis use disorder. Cochrane Database of Systematic Reviews [Internet]. 2016; (5). Available from: <http://onlinelibrary.wiley.com/doi/10.1002/14651858.CD005336.pub4/abstract>; <http://onlinelibrary.wiley.com/store/10.1002/14651858.CD005336.pub4/asset/CD005336.pdf?v=1&t=ioig2jkq&s=3973c2abe787bfb16f223d7cc0f1339d4e6d1ba4>.

21. Joseph J, Basu DD, M., Krishnan N. Are nurse-conducted brief interventions (NCBIs) efficacious for hazardous or harmful alcohol use? A systematic review (Provisional abstract). Database of Abstracts of Reviews of Effects [Internet]. 2014; (2):[203-10 pp.]. Available from: <http://onlinelibrary.wiley.com/o/cochrane/cldare/articles/DARE-12014021999/frame.html>.

22. Jiang S, Wu L, Gao X. Beyond face-to-face individual counseling: A systematic review on alternative modes of motivational interviewing in substance abuse treatment and prevention. Addictive behaviors. 2017;73:216-35. doi: <https://dx.doi.org/10.1016/j.addbeh.2017.05.023>.

23. Joseph J, Basu D. Efficacy of Brief Interventions in Reducing Hazardous or Harmful Alcohol Use in Middle-Income Countries: Systematic Review of Randomized Controlled Trials. Alcohol and alcoholism (Oxford, Oxfordshire). 2017;52(1):56-64. doi: <https://dx.doi.org/10.1093/alcalc/agw054>.

24. Klimas J, Field C-A, Cullen W, O'Gorman CSM, Glynn Liam G, Keenan E, et al. Psychosocial interventions to reduce alcohol consumption in concurrent problem alcohol and illicit drug users. Cochrane Database of Systematic Reviews [Internet]. 2012; (11). Available from: <http://onlinelibrary.wiley.com/doi/10.1002/14651858.CD009269.pub2/abstracthttp://onlinelibrary.wiley.com/store/10.1002/14651858.CD009269.pub2/asset/CD009269.pdf?v=1&t=i2qmh9bk&s=8643c3b4208684381ba8c57d902b242ca63d42f1>.

25. Foxcroft DR, Coombes L, Wood S, Allen D, Almeida Santimano NML. Motivational interviewing for alcohol misuse in young adults. The Cochrane database of systematic reviews. 2014;8:CD007025.

26. Gilinsky A, VivienPower, Kevin. Interventions delivered during antenatal care to reduce alcohol consumption during pregnancy: A systematic review. Addiction Research & Theory. 2011;19(3):235-50. PubMed PMID: 2011-08678-005.

27. Livingston JD, Milne T, Fang ML, Amari E. The effectiveness of interventions for reducing stigma related to substance use disorders: A systematic review. Addiction. 2012;107(1):39-50. PubMed PMID: 2011-29128-010.

28. McMurran M. Motivational interviewing with offenders: A systematic review. Legal and Criminological Psychology. 2009;14(1):83-100. PubMed PMID: 2009-02935-008.

29. Seigers DKL, Carey KB. Screening and brief interventions for alcohol use in college health centers: A review. Journal of American College Health. 2010;59(3):151-8. PubMed PMID: 2011-03610-003.

30. Smedslund GRC, Hammerstrøm Karianne T, Steiro AK, A., Dahl Helene MK, Kjetil. Motivational interviewing for substance abuse. Cochrane Database of Systematic Reviews [Internet]. 2011; (5). Available from: <http://onlinelibrary.wiley.com/doi/10.1002/14651858.CD008063.pub2/abstracthttp://onlinelibrary.wiley.com/store/10.1002/14651858.CD008063.pub2/asset/CD008063.pdf?v=1&t=i2qmfc7p&s=9cd557e022d5878b20c45f506b715404da5f5945>.

31. Tanner-Smith EE, Lipsey MW. Brief alcohol interventions for adolescents and young adults: a systematic review and meta-analysis. J Subst Abuse Treat. 2015;51:1-18. doi: 10.1016/j.jsat.2014.09.001. PubMed PMID: 25300577; PubMed Central PMCID: PMCPMC4346408.

32. Terplan M, Lui S. Psychosocial interventions for pregnant women in outpatient illicit drug treatment programs compared to other interventions. Cochrane Database Syst Rev. 2007;(4):CD006037. doi: 10.1002/14651858.CD006037.pub2. PubMed PMID: 17943878.

33. Terplan M, Ramanadhan S, Locke A, Longinaker N, Lui S. Psychosocial interventions for pregnant women in outpatient illicit drug treatment programs compared to other interventions. Cochrane Database of Systematic Reviews [Internet]. 2015; (4). Available from: <http://onlinelibrary.wiley.com/doi/10.1002/14651858.CD006037.pub3/abstract>; <http://onlinelibrary.wiley.com/store/10.1002/14651858.CD006037.pub3/asset/CD006037.pdf?v=1&t=ioig2s9v&s=fbc50772d8ebf81e1c60cf130a7a926f1b16f906>.

34. Vasilaki EI, Hosier SG, Cox WM. The efficacy of motivational interviewing as a brief intervention for excessive drinking: A meta-analytic review. Alcohol and Alcoholism. 2006;41(3):328-35. PubMed PMID: 2006-05794-019.

35. Baker AL, Hiles SA, Thornton LK, Hides L, Lubman DI. A systematic review of psychological interventions for excessive alcohol consumption among people with psychotic disorders. Acta Psychiatr Scand. 2012;126(4):243-55. doi: 10.1111/j.1600-0447.2012.01885.x. PubMed PMID: 22632145.

36. Baker AL, Thornton LK, Hiles S, Hides L, Lubman DI. Psychological interventions for alcohol misuse among people with co-occurring depression or anxiety disorders: A systematic review. Journal of Affective Disorders. 2012;139(3):217-29. PubMed PMID: 2012-16582-003.

37. Boniface S, Malet-Lambert I, Coleman R, Deluca P, Donoghue K, Drummond C, et al. The Effect of Brief Interventions for Alcohol Among People with Comorbid Mental Health Conditions: A Systematic Review of Randomized Trials and Narrative Synthesis. Alcohol & Alcoholism. 2018;53(3):282-93. doi: 10.1093/alcalc/agx111. PubMed PMID: 129422917. Language: English. Entry Date: 20180510. Revision Date: 20180519. Publication Type: Article. Journal Subset: Biomedical.

38. Cleary M, Hunt GE, Matheson S, Walter G. Psychosocial treatments for people with co-occurring severe mental illness and substance misuse: systematic review. J Adv Nurs. 2009;65(2):238-58. doi: 10.1111/j.1365-2648.2008.04879.x. PubMed PMID: 19016921.

39. de Man-van Ginkel JM, Gooskens F, Schuurmans MJ, Lindeman E, Hafsteinsdottir TB, Rehabilitation Guideline Stroke Working G. A systematic review of therapeutic interventions for poststroke depression and the role of nurses. J Clin Nurs. 2010;19(23-24):3274-90. doi: 10.1111/j.1365-2702.2010.03402.x. PubMed PMID: 21083778.

40. Hjorthøj C, Fohlmann A, Nordentoft M. Treatment of cannabis use disorders in people with schizophrenia spectrum disorders—A systematic review. Addictive Behaviors. 2009;34(6-7):520-5. PubMed PMID: 2009-07777-005.

41. Kelly TM, Daley DC, Douaihy AB. Treatment of substance abusing patients with comorbid psychiatric disorders. Addict Behav. 2012;37(1):11-24. doi: 10.1016/j.addbeh.2011.09.010. PubMed PMID: 21981788; PubMed Central PMCID: PMC3196788.

42. Laker CJ. How reliable is the current evidence looking at the efficacy of harm reduction and motivational interviewing interventions in the treatment of patients with a dual diagnosis? Journal of Psychiatric and Mental Health Nursing. 2007;14(8):720-6. PubMed PMID: 2007-18385-002.

43. Cowlishaw S, Merkouris S, Dowling N, Anderson C, Jackson A, Thomas S. Psychological therapies for pathological and problem gambling. Cochrane Database of Systematic Reviews [Internet]. 2012; (11). Available from: <http://onlinelibrary.wiley.com/doi/10.1002/14651858.CD008937.pub2/abstracthttp://onlinelibrary.wiley.com/store/10.1002/14651858.CD008937.pub2/asset/CD008937.pdf?v=1&t=i2qmh2a4&s=24442e6c86853f078ee853bb6912770fd69af25b>.

44. Petry NM, Ginley MK, Rash CJ. Systematic review of gambling. Psychology of addictive behaviors : journal of the Society of Psychologists in Addictive Behaviors. 2017;31(8):951-61. doi: <https://dx.doi.org/10.1037/adb0000290>.

45. Yakovenko I, Quigley L, Hemmelgarn BR, Hodgins DC, Ronksley P. The efficacy of motivational interviewing for disordered gambling: systematic review and meta-analysis. Addict Behav. 2015;43:72-82. doi: 10.1016/j.addbeh.2014.12.011. PubMed PMID: 25577724.

46. Alperstein D, Sharpe L. The Efficacy of Motivational Interviewing in Adults With Chronic Pain: A Meta-Analysis and Systematic Review. J Pain. 2016;17(4):393-403. doi: 10.1016/j.jpain.2015.10.021. PubMed PMID: 26639413.

47. Chilton R, Pires-Yfantouda R, Wylie M. A systematic review of motivational interviewing within musculoskeletal health. Psychology, Health & Medicine. 2012;17(4):392-407. PubMed PMID: 2012-18217-002.

48. Cascaes AM, Bielemann RM, Clark VL, Barros A. Effectiveness of motivational interviewing at improving oral health: a systematic review. Revista de saude publica. 2014;48(1):142-53.

49. Gao XML, E. C.Ching Ching Kot, S.Wai Chan, K. C. Motivational interviewing in improving oral health: a systematic review of randomized controlled trials (Provisional abstract). Journal of Periodontology [Internet]. 2014; 85(3):[426-37 pp.]. Available from: <http://onlinelibrary.wiley.com/o/cochrane/cldare/articles/DARE-12013037772/frame.html>.

50. Kay EJ, Vascott D, Hocking A, Nield H. Motivational interviewing in general dental practice: A review of the evidence. British dental journal. 2016;221(12):785-91. Epub 2016/12/17. doi: 10.1038/sj.bdj.2016.952. PubMed PMID: 27982007.

51. Kopp SL, Ramseier CA, Ratka-Kruger P, Woelber JP. Motivational Interviewing As an Adjunct to Periodontal Therapy-A Systematic Review. Frontiers in psychology. 2017;8:279. doi: <https://dx.doi.org/10.3389/fpsyg.2017.00279>.

52. Werner H, Hakeberg M, Dahlstrom L, Eriksson M, Sjogren P, Strandell A, et al. Psychological Interventions for Poor Oral Health: A Systematic Review. Journal of dental research. 2016;95(5):506-14. doi: <https://dx.doi.org/10.1177/0022034516628506>.

53. Macdonald P, Hibbs R, Corfield F, Treasure J. The use of motivational interviewing in eating disorders: A systematic review. Psychiatry Research. 2012;200(1):1-11. PubMed PMID: 2012-16485-001.

54. Knowles L, Anokhina A, Serpell L. Motivational interventions in the eating disorders: What is the evidence? International Journal of Eating Disorders. 2013;46(2):97-107. PubMed PMID: 2013-04604-001.

55. Dray J, Tracey D. Is the transtheoretical model and motivational interviewing approach applicable to the treatment of eating disorders? A review. Clinical Psychology Review. 2012;32(6):558-65. PubMed PMID: 2012-22130-012.

56. Armstrong MJ, Mottershead TA, Ronksley PE, Sigal RJ, Campbell TS, Hemmelgarn BR. Motivational interviewing to improve weight loss in overweight and/or obese patients: a systematic review and meta-analysis of randomized controlled trials. Obes Rev. 2011;12(9):709-23. doi: 10.1111/j.1467-789X.2011.00892.x. PubMed PMID: 21692966.

57. Barnes RD, Ivezaj V. A systematic review of motivational interviewing for weight loss among adults in primary care. Obesity reviews : an official journal of the International Association for the Study of Obesity. 2015;16(4):304-18.

58. Hill B, Skouteris H, Fuller-Tyszkiewicz M. Interventions designed to limit gestational weight gain: a systematic review of theory and meta-analysis of intervention components. Obes Rev. 2013;14(6):435-50. doi: 10.1111/obr.12022. PubMed PMID: 23534901.

59. VanWormer JJ, Boucher JL. Motivational interviewing and diet modification: a review of the evidence (Provisional abstract). Diabetes Educator [Internet]. 2004; 30(3):[404-16 pp.]. Available from: <http://onlinelibrary.wiley.com/o/cochrane/cldare/articles/DARE-12004001639/frame.html>.

60. Clifford Mulimba AA, Byron-Daniel J. Motivational interviewing-based interventions and diabetes mellitus (Provisional abstract). British Journal of Nursing [Internet]. 2014; 23(1):[8-14 pp.]. Available from: <http://onlinelibrary.wiley.com/o/cochrane/cldare/articles/DARE-12014006260/frame.html>.

61. Ekong G, Kavookjian J. Motivational interviewing and outcomes in adults with type 2 diabetes: A systematic review. Patient education and counseling. 2016;99(6):944-52. doi: <https://dx.doi.org/10.1016/j.pec.2015.11.022>.

62. Jones A, Gladstone BP, Lubeck M, Lindekilde N, Upton D, Vach W. Motivational interventions in the management of HbA1c levels: a systematic review and meta-analysis (Provisional abstract). Database of Abstracts of Reviews of Effects [Internet]. 2014; (2):[91-100 pp.]. Available from: <http://onlinelibrary.wiley.com/o/cochrane/cldare/articles/DARE-12014015299/frame.html>.

63. Lin CH, Chiang SL, Tzeng WC, Chiang LC. Systematic review of impact of lifestyle-modification programs on metabolic risks and patient-reported outcomes in adults with metabolic syndrome. Worldviews Evid Based Nurs. 2014;11(6):361-8. doi: 10.1111/wvn.12069. PubMed PMID: 25488565.

64. Soderlund PD. Effectiveness of motivational interviewing for improving physical activity self-management for adults with type 2 diabetes: A review. Chronic Illness. 2018;14(1):54-68. doi: 10.1177/1742395317699449. PubMed PMID: 128071638. Language: English. Entry Date: 20180307. Revision Date: 20180307. Publication Type: Article.

65. Thepwongsa I, Muthukumar R, Kessomboon P. Motivational interviewing by general practitioners for Type 2 diabetes patients: a systematic review. Family practice. 2017;34(4):376-83. doi: <https://dx.doi.org/10.1093/fampra/cmx045>.

66. Cheng D, Qu Z, Huang J, Xiao Y, Luo H, Wang J. Motivational interviewing for improving recovery after stroke. Cochrane Database Syst Rev. 2015;6(6):CD011398. doi: 10.1002/14651858.CD011398.pub2. PubMed PMID: 26037617.

67. Hildebrand MW. Effectiveness of interventions for adults with psychological or emotional impairment after stroke: An evidence-based review. American Journal of Occupational Therapy. 2015;69(1):6901180050p1-9.

68. Lee WW, Choi KC, Yum RW, Yu DS, Chair SY. Effectiveness of motivational interviewing on lifestyle modification and health outcomes of clients at risk or diagnosed with cardiovascular diseases: A systematic review. Int J Nurs Stud. 2016;53:331-41. doi: 10.1016/j.ijnurstu.2015.09.010. PubMed PMID: 26493130.

69. Berg RC, Ross MW, Tikkanen R. The effectiveness of MI4MSM: How useful is motivational interviewing as an HIV risk prevention program for men who have sex with men? A systematic review. AIDS Education and Prevention. 2011;23(6):533-49. PubMed PMID: 2011-29905-005.

70. Carrico AW, Zepf R, Meanley S, Batchelder A, Stall R. Critical review: When the party is over: A systematic review of behavioral interventions for substance-using men who have sex with men. JAIDS Journal of Acquired Immune Deficiency Syndromes. 2016;73(3):299-306. doi: 10.1097/QAI.0000000000001102. PubMed PMID: 2016-52706-010.

71. Dillard PK, Zuniga JA, Holstad MM. An integrative review of the efficacy of motivational interviewing in HIV management. Patient Education & Counseling. 2017;100(4):636-46. doi: 10.1016/j.pec.2016.10.029. PubMed PMID: 122328487. Language: English. Entry Date: 20180108. Revision Date: 20180424. Publication Type: journal article.

72. Naar-King S, Parsons JT, Johnson AM. Motivational interviewing targeting risk reduction for people with HIV: a systematic review. Curr HIV/AIDS Rep. 2012;9(4):335-43. doi: 10.1007/s11904-012-0132-x. PubMed PMID: 22890780.

73. Wilson A, Nirantharakumar K, Truchanowicz EG, Surenthirakumaran R, MacArthur C, Coomarasamy A. Motivational interviews to improve contraceptive use in populations at high risk of unintended pregnancy: a systematic review and meta-analysis. European journal of obstetrics, gynecology, and reproductive biology. 2015;191:72-9.

74. Karmali Kunal N, Davies P, Taylor F, Beswick A, Martin N, Ebrahim S. Promoting patient uptake and adherence in cardiac rehabilitation. Cochrane Database of Systematic Reviews [Internet]. 2014; (6). Available from: <http://onlinelibrary.wiley.com/doi/10.1002/14651858.CD007131.pub3/abstract>; <http://onlinelibrary.wiley.com/store/10.1002/14651858.CD007131.pub3/asset/CD007131.pdf?v=1&t=ioig22rk&s=e2dd028ffe00c7a62f19317069105176b6785a29>.

75. Lawrence P, Fulbrook P, Somerset S, Schulz P. Motivational interviewing to enhance treatment attendance in mental health settings: A systematic review and meta-analysis. Journal of psychiatric and mental health nursing. 2017;24(9-10):699-718. doi: <https://dx.doi.org/10.1111/jpm.12420>.

76. Miller SJ, Foran-Tuller K, Ledergerber J, Jandorf L. Motivational interviewing to improve health screening uptake: A systematic review. Patient education and counseling. 2017;100(2):190-8. doi: <https://dx.doi.org/10.1016/j.pec.2016.08.027>.

77. Al-Ganmi AH, Perry L, Gholizadeh L, Alotaibi AM. Cardiovascular medication adherence among patients with cardiac disease: a systematic review. Journal of advanced nursing. 2016;72(12):3001-14. doi: <https://dx.doi.org/10.1111/jan.13062>.

78. Binford M, Kahana SY, Altice FL. A systematic review of antiretroviral adherence interventions for HIV-infected people who use drugs. Curr HIV/AIDS Rep. 2012;9(4):287-312. doi: 10.1007/s11904-012-0134-8. PubMed PMID: 22936463; PubMed Central PMCID: PMC3495269.

79. Easthall C, Song F, Bhattacharya D. A meta-analysis of cognitive-based behaviour change techniques as interventions to improve medication adherence (Provisional abstract). BMJ Open [Internet]. 2013; 3(8):[e002749 p.]. Available from: <http://onlinelibrary.wiley.com/o/cochrane/cldare/articles/DARE-12013049255/frame.html>.

80. Hill S, Kavookjian J. Motivational interviewing as a behavioral intervention to increase HAART adherence in patients who are HIV-positive: A systematic review of the literature. AIDS Care. 2012;24(5):583-92. PubMed PMID: 2012-11855-006.

81. Hu D, Juarez DT, Yeboah M, Castillo TP. Interventions to increase medication adherence in African-American and Latino populations: a literature review. Hawaii J Med Public Health. 2014;73(1):11-8. PubMed PMID: 24470982; PubMed Central PMCID: PMCPMC3901167.

82. Nieuwlaat R, Wilczynski N, Navarro T, Hobson N, Jeffery R, Keepanasseril A, et al. Interventions for enhancing medication adherence. Cochrane Database Syst Rev. 2014;11:CD000011. doi: 10.1002/14651858.CD000011.pub4. PubMed PMID: 25412402.

83. Palacio A, Garay D, Langer B, Taylor J, Wood BA, Tamariz L. Motivational Interviewing Improves Medication Adherence: a Systematic Review and Meta-analysis. Journal of general internal medicine. 2016;31(8):929-40. doi: <https://dx.doi.org/10.1007/s11606-016-3685-3>.

84. Rueda S-WL, Y.Bayoumi, AhmedTynan, Anne-MarieAntoniou, TonyRourke, SeanGlazier, Richard. Patient support and education for promoting adherence to highly active antiretroviral therapy for HIV/AIDS. Cochrane Database of Systematic Reviews [Internet]. 2006; (3). Available from: <http://onlinelibrary.wiley.com/doi/10.1002/14651858.CD001442.pub2/abstracthttp://onlinelibrary.wiley.com/store/10.1002/14651858.CD001442.pub2/asset/CD001442.pdf?v=1&t=i2qm0u5a&s=f90055300e9c0cc4852f27cea860ba2824ed1afa>.

85. Zomahoun HTV, Guenette L, Gregoire J-P, Lauzier S, Lawani AM, Ferdynus C, et al. Effectiveness of motivational interviewing interventions on medication adherence in adults with chronic diseases: a systematic review and meta-analysis. International journal of epidemiology. 2017;46(2):589-602. doi: <https://dx.doi.org/10.1093/ije/dyw273>.

86. Spencer JC, Wheeler SB. A systematic review of Motivational Interviewing interventions in cancer patients and survivors. Patient education and counseling. 2016;99(7):1099-105. doi: <https://dx.doi.org/10.1016/j.pec.2016.02.003>.

87. Wagoner ST, Kavookjian J. The Influence of Motivational Interviewing on Patients With Inflammatory Bowel Disease: A Systematic Review of the Literature. Journal of clinical medicine research. 2017;9(8):659-66. doi: <https://dx.doi.org/10.14740/jocmr3081w>.

88. Burke BL, Arkowitz H, Menchola M. The efficacy of motivational interviewing: a meta-analysis of controlled clinical trials. J Consult Clin Psychol. 2003;71(5):843-61. doi: 10.1037/0022-006X.71.5.843. PubMed PMID: 14516234.

89. Dunn C, Deroo L, Rivara FP. The use of brief interventions adapted from motivational interviewing across behavioral domains: A systematic review. Addiction. 2001;96(12):1725-42. PubMed PMID: 2001-09576-001.

90. Hettema J, Steele J, Miller WR. Motivational interviewing. Annu Rev Clin Psychol. 2005;1:91-111. doi: 10.1146/annurev.clinpsy.1.102803.143833. PubMed PMID: 17716083.

91. Lundahl BW, Kunz C, Brownell C, Tollefson D, Burke BL. A meta-analysis of motivational interviewing: twenty-five years of empirical studies (Provisional abstract). Research on Social Work Practice [Internet]. 2010; 20(2):[137-60 pp.]. Available from: <http://onlinelibrary.wiley.com/o/cochrane/cldare/articles/DARE-12010003750/frame.html>.

92. Martins RK, McNeil DW. Review of Motivational Interviewing in promoting health behaviors. Clin Psychol Rev. 2009;29(4):283-93. doi: 10.1016/j.cpr.2009.02.001. PubMed PMID: 19328605.

93. O’Halloran PD, Blackstock F, Shields N, Holland A, Iles R, Kingsley M, et al. Motivational interviewing to increase physical activity in people with chronic health conditions: A systematic review and meta-analysis. Clinical Rehabilitation. 2014;28(12):1159-71. doi: 10.1177/0269215514536210. PubMed PMID: 2014-48880-002.

94. Rubak S, Sandbaek A, Lauritzen T, Christensen B. Motivational interviewing: a systematic review and meta-analysis. The British journal of general practice : the journal of the Royal College of General Practitioners. 2005;55(513):305-12. Epub 2005/04/14. PubMed PMID: 15826439; PubMed Central PMCID: PMC1463134.

95. Shingleton RM, Palfai TP. Technology-delivered adaptations of motivational interviewing for health-related behaviors: A systematic review of the current research. Patient education and counseling. 2016;99(1):17-35. doi: <https://dx.doi.org/10.1016/j.pec.2015.08.005>.

96. Thompson DR, Chair SY, Chan SW, Astin F, Davidson PM, Ski CF. Motivational interviewing: a useful approach to improving cardiovascular health? J Clin Nurs. 2011;20(9-10):1236-44. doi: 10.1111/j.1365-2702.2010.03558.x. PubMed PMID: 21492271.

97. Kohler S, Hofmann A. Can motivational interviewing in emergency care reduce alcohol consumption in young people? A systematic review and meta-analysis. Alcohol and alcoholism (Oxford, Oxfordshire). 2015;50(2):107-17.

98. Knight KM, McGowan L, Dickens C, Bundy C. A systematic review of motivational interviewing in physical health care settings. Br J Health Psychol. 2006;11(Pt 2):319-32. doi: 10.1348/135910705X52516. PubMed PMID: 16643702.

99. Lundahl B, Moleni T, Burke BL, Butters R, Tollefson D, Butler C, et al. Motivational interviewing in medical care settings: a systematic review and meta-analysis of randomized controlled trials (Structured abstract). Patient Education and Counseling [Internet]. 2013; 93(2):[157-68 pp.]. Available from: <http://onlinelibrary.wiley.com/o/cochrane/cldare/articles/DARE-12013052286/frame.htmlhttp://www.pec-journal.com/article/S0738-3991(13)00288-7/pdf>.

100. Merz V, Baptista J, Haller DM. Brief interventions to prevent recurrence and alcohol-related problems in young adults admitted to the emergency ward following an alcohol-related event: a systematic review. J Epidemiol Community Health. 2015;69(9):912-7. doi: 10.1136/jech-2014-204824. PubMed PMID: 25841242.

101. Noordman J, Weijden T, Dulmen S. Communication-related behavior change techniques used in face-to-face lifestyle interventions in primary care: a systematic review of the literature (Structured abstract). Patient Education and Counseling [Internet]. 2012; 89(2):[227-44 pp.]. Available from: <http://onlinelibrary.wiley.com/o/cochrane/cldare/articles/DARE-12012055644/frame.html>.

102. Purath J, Keck A, Fitzgerald CE. Motivational interviewing for older adults in primary care: a systematic review. Geriatr Nurs. 2014;35(3):219-24. doi: 10.1016/j.gerinurse.2014.02.002. PubMed PMID: 24656051.

103. Taggart J, Williams A, Dennis S, Newall A, Shortus T, Zwar N, et al. A systematic review of interventions in primary care to improve health literacy for chronic disease behavioral risk factors. BMC family practice. 2012;13:49. doi: 10.1186/1471-2296-13-49. PubMed PMID: 22656188; PubMed Central PMCID: PMC3444864.

104. VanBuskirk KAL, Wetherell J. Motivational interviewing with primary care populations: a systematic review and meta-analysis (Provisional abstract). Journal of Behavioral Medicine [Internet]. 2014; 37(4):[768-80 pp.]. Available from: <http://onlinelibrary.wiley.com/o/cochrane/cldare/articles/DARE-12013048261/frame.html>

<http://www.ncbi.nlm.nih.gov/pmc/articles/PMC4118674/pdf/nihms587843.pdf>.
