# Supplementary material for: Effectiveness of Motivational Interviewing on adult behaviour change in health and social care settings: A systematic review of reviews
Source: PLoS One. 2018 Oct 18;13(10):e0204890. doi: 10.1371/journal.pone.0204890 (PMC6193639; doi:10.1371/journal.pone.0204890)
Supplement: S2 Table — (DOCX) [file pone.0204890.s003.docx]

# S2 Table. Quality assessment of included reviews based on ROBIS (risk of bias in systematic reviews) tool.

*Indicates review including meta-analyses

| **Author and year** | **Domain 1: Concerns regarding specification of study eligibility criteria** | **Domain 2:**  **Concerns regarding methods used to identify and/or select studies** | **Domain 3: Concerns regarding methods used to identify and/or select studies** | **Domain 4: Concerns regarding methods used to identify and/or select studies** | **Risk of Bias** |
| --- | --- | --- | --- | --- | --- |
| Al-Ganmi,et al (2016) | LOW | UNCLEAR | LOW | LOW | LOW |
| Alperstein & Sharpe (2016) * | LOW | LOW | LOW | LOW | LOW |
| Appiah-Brempong et al (2014) | UNCLEAR | HIGH | HIGH | HIGH | HIGH |
| Armstrong et al (2011) * | LOW | LOW | LOW | LOW | LOW |
| Baker, Hiles, S. A.Thornton, et al (2012) | LOW | HIGH | LOW | UNCLEAR | UNCLEAR |
| Baker, Thornton, Hiles, Hides, et al (2012) | UNCLEAR | HIGH | LOW | LOW | UNCLEAR |
| Barnes et al (2015) | HIGH | HIGH | HIGH | HIGH | HIGH |
| Barrio et al (2016) | LOW | LOW | LOW | LOW | LOW |
| Baxi et al (2014) | LOW | UNCLEAR | LOW | LOW | LOW |
| Baxter et al (2011) | LOW | UNCLEAR | HIGH | UNCLEAR | UNCLEAR |
| Behbod et al (2018) | LOW | LOW | LOW | LOW | LOW |
| Berg et al (2011)* | LOW | LOW | LOW | LOW | LOW |
| Binford et al (2012) | LOW | UNCLEAR | HIGH | UNCLEAR | UNCLEAR |
| Boniface et al (2018) | LOW | LOW | LOW | LOW | LOW |
| Branscum et al (2010) | UNCLEAR | HIGH | HIGH | HIGH | HIGH |
| Burke et al (2003)* | LOW | HIGH | LOW | LOW | UNCLEAR |
| Carey et al (2007)* | LOW | UNCLEAR | UNCLEAR | LOW | UNCLEAR |
| Carey et al (2012)* | LOW | UNCLEAR | UNCLEAR | LOW | UNCLEAR |
| Carrico et al (2016) | UNCLEAR | HIGH | HIGH | UNCLEAR | HIGH |
| Cascaes et al (2014) | LOW | LOW | HIGH | UNCLEAR | UNCLEAR |
| Chatters et al (2016) | LOW | LOW | LOW | LOW | LOW |
| Cheng et al (2015)* | LOW | LOW | LOW | LOW | LOW |
| Chilton et al (2012) | LOW | UNCLEAR | LOW | UNCLEAR | UNCLEAR |
| Cleary et al (2009) | LOW | LOW | LOW | LOW | LOW |
| Clifford and Byron-Daniel (2004) | LOW | HIGH | LOW | UNCLEAR | UNCLEAR |
| Cooper et al (2015) | LOW | LOW | LOW | LOW | LOW |
| Cowlishaw et al (2012)* | LOW | LOW | LOW | LOW | LOW |
| Darker et al (2015)* | LOW | LOW | LOW | LOW | LOW |
| De Man-van Ginkel et al, (2010) | LOW | UNCLEAR | LOW | UNCLEAR | UNCLEAR |
| Dillard et al (2016) | HIGH | HIGH | UNCLEAR | UNCLEAR | UNCLEAR |
| Dray and Wade (2012) | UNCLEAR | HIGH | HIGH | HIGH | HIGH |
| Dunn et al (2001) | LOW | UNCLEAR | HIGH | UNCLEAR | UNCLEAR |
| Easthall et al (2013)* | LOW | LOW | LOW | LOW | LOW |
| Ebbert et al (2015)* | LOW | LOW | LOW | LOW | LOW |
| Ekong and Kavookjian (2016) | UNCLEAR | UNCLEAR | LOW | UNCLEAR | UNCLEAR |
| Foxcroft et al (2014)* | LOW | LOW | LOW | LOW | LOW |
| Gao et al (2014) | LOW | UNCLEAR | UNCLEAR | UNCLEAR | UNCLEAR |
| Gates et al (2016)* | LOW | LOW | LOW | LOW | LOW |
| Gilinsky et al (2011) | LOW | UNCLEAR | LOW | UNCLEAR | UNCLEAR |
| Heckman et al (2010)* | UNCLEAR | HIGH | HIGH | HIGH | HIGH |
| Hettema et al (2005)* | UNCLEAR | HIGH | LOW | LOW | UNCLEAR |
| Hettema et al (2010)* | LOW | HIGH | LOW | LOW | UNCLEAR |
| Hildebrand (2015) | HIGH | HIGH | HIGH | HIGH | HIGH |
| Hill and Kavookjian (2012) | LOW | UNCLEAR | UNCLEAR | UNCLEAR | UNCLEAR |
| Hill et al (2013)* | LOW | UNCLEAR | UNCLEAR | LOW | UNCLEAR |
| Hjorthoj et al (2009) | LOW | UNCLEAR | LOW | UNCLEAR | UNCLEAR |
| Hu et al (2014) | HIGH | HIGH | HIGH | HIGH | HIGH |
| Jiang et al (2017) | LOW | UNCLEAR | LOW | LOW | LOW |
| Jones et al (2014)* | LOW | HIGH | UNCLEAR | UNCLEAR | HIGH |
| Joseph et al (2014) | UNCLEAR | HIGH | UNCLEAR | UNCLEAR | UNCLEAR |
| Joseph et al (2017) | HIGH | HIGH | UNCLEAR | UNCLEAR | HIGH |
| Karmali et al (2014) | LOW | LOW | LOW | LOW | LOW |
| Kay et al ( 2016) | LOW | LOW | LOW | LOW | LOW |
| Kelly et al (2012) | UNCLEAR | HIGH | HIGH | HIGH | HIGH |
| Klimas et al (2012) | LOW | LOW | LOW | LOW | LOW |
| Knight et al (2006) | LOW | UNCLEAR | LOW | UNCLEAR | UNCLEAR |
| Knowles et al (2013) | UNCLEAR | HIGH | HIGH | UNCLEAR | HIGH |
| Kohler & Hofmann (2015)* | LOW | UNCLEAR | UNCLEAR | UNCLEAR | UNCLEAR |
| Kopp et al (2017) | LOW | UNCLEAR | UNCLEAR | LOW | UNCLEAR |
| Laker, C. J.(2007) | LOW | HIGH | HIGH | UNCLEAR | HIGH |
| Lawrence et al (2017)* | LOW | LOW | UNCLEAR | LOW | LOW |
| Lee et al (2016) | LOW | LOW | UNCLEAR | LOW | LOW |
| Lin et al (2014) | LOW | HIGH | LOW | UNCLEAR | UNCLEAR |
| Lindson-Hawley et al (2015)* | LOW | LOW | LOW | LOW | LOW |
| Livingston et al (2012) | LOW | LOW | LOW | LOW | LOW |
| Lundahl et al ( 2013)* | LOW | UNCLEAR | UNCLEAR | UNCLEAR | UNCLEAR |
| Lundahl et al (2010)* | LOW | UNCLEAR | UNCLEAR | UNCLEAR | UNCLEAR |
| Macdonald et al (2012) | LOW | UNCLEAR | UNCLEAR | UNCLEAR | UNCLEAR |
| Mantler et al (2012) | LOW | HIGH | UNCLEAR | UNCLEAR | UNCLEAR |
| Martins and McNeil (2009) | HIGH | HIGH | HIGH | HIGH | HIGH |
| McMurran, Mary ( 2009) | LOW | UNCLEAR | HIGH | UNCLEAR | HIGH |
| Merz et al (2015) | LOW | UNCLEAR | LOW | LOW | LOW |
| Miller et al (2017) | LOW | UNCLEAR | UNCLEAR | LOW | UNCLEAR |
| Naar-King et al (2012) | LOW | UNCLEAR | HIGH | HIGH | HIGH |
| Nieuwlaat et al (2008) | LOW | LOW | LOW | LOW | LOW |
| Noordman et al (2012) | LOW | LOW | LOW | UNCLEAR | LOW |
| O'Halloran et al (2014)* | LOW | LOW | LOW | LOW | LOW |
| Palacio et al (2016)* | LOW | UNCLEAR | LOW | LOW | UNCLEAR |
| Pelletier et al (2014) | LOW | UNCLEAR | LOW | LOW | LOW |
| Petry et al (2017) | UNCLEAR | UNCLEAR | HIGH | HIGH | HIGH |
| Purath et al (2014) | LOW | UNCLEAR | UNCLEAR | LOW | UNCLEAR |
| Rabe et al (2013)* | LOW | LOW | LOW | LOW | LOW |
| Rubak et al (2005)* | LOW | UNCLEAR | UNCLEAR | LOW | UNCLEAR |
| Rueda et al (2006) | LOW | LOW | LOW | LOW | LOW |
| Seigers et al (2010) | LOW | UNCLEAR | HIGH | HIGH | HIGH |
| Shingleton et al (2016) | HIGH | HIGH | HIGH | HIGH | HIGH |
| Smedslund et al (2011)* | LOW | LOW | LOW | LOW | LOW |
| Soderlund et al (2018) | HIGH | HIGH | HIGH | HIGH | HIGH |
| Spencer et al ( 2016) | HIGH | HIGH | HIGH | HIGH | HIGH |
| Stead et al (2016)* | LOW | UNCLEAR | LOW | LOW | LOW |
| Taggart et al (2012) | LOW | LOW | LOW | LOW | LOW |
| Tanner-Smith et al (2015)* | LOW | UNCLEAR | UNCLEAR | LOW | UNCLEAR |
| Terplan et al (2007)* | LOW | LOW | LOW | LOW | LOW |
| Terplan et al (2015)* | LOW | LOW | LOW | LOW | LOW |
| Thepwongsa et al (2017) | LOW | UNCLEAR | HIGH | UNCLEAR | UNCLEAR |
| Thompson et al (2011) | LOW | UNCLEAR | UNCLEAR | HIGH | HIGH |
| VanBuskirk & Wetherell (2014) * | LOW | UNCLEAR | UNCLEAR | LOW | UNCLEAR |
| VanWormer and Boucher (2004) * | UNCLEAR | HIGH | UNCLEAR | HIGH | HIGH |
| Vasilaki et al (2006)* | LOW | UNCLEAR | UNCLEAR | LOW | UNCLEAR |
| Wagonera, et al (2017) | UNCLEAR | HIGH | UNCLEAR | UNCLEAR | UNCLEAR |
| Werner et al (2016)* | LOW | LOW | UNCLEAR | UNCLEAR | UNCLEAR |
| Wilson et al (2015)* | UNCLEAR | UNCLEAR | UNCLEAR | UNCLEAR | UNCLEAR |
| Yakovenko et (2015)* | LOW | LOW | LOW | LOW | LOW |
| Zomahounet al (2017) * | LOW | LOW | LOW | LOW | LOW |
