# Supplementary material for: Effectiveness of Motivational Interviewing on adult behaviour change in health and social care settings: A systematic review of reviews
Source: PLoS One. 2018 Oct 18;13(10):e0204890. doi: 10.1371/journal.pone.0204890 (PMC6193639; doi:10.1371/journal.pone.0204890)
Supplement: S3 Table — (DOCX) [file pone.0204890.s004.docx]

| **Health behaviour** | **Reference to review** | **Comparison** | **Population** | **Outcome** | **Assessment times**  **(if stated)** | **N of studies** | **n (total)** | **Effect size [95% confidence interval)** | **GRADE LEVEL** | **1. ROB**  **2. Imprecision**  **3. Inconsistency**  **4. Indirectness**  **5. Publication bias** | **GRADE applied by authors of:** |
| --- | --- | --- | --- | --- | --- | --- | --- | --- | --- | --- | --- |
| Alcohol | Vasilaki et al 2006[1] | NT | Any | Reducing alcohol consumption |  | 9 | 1587 | ES 0.18 [0.07, 0.29] | ⊕⊕⊝⊝  LOW | 1. D^1^  2. -  3. D^1^  4. -  5. - | overview |
|  | Foxcroft et al 2014 [2] | No MI comparison | Young people (<25 years) | Alcohol problems | <4 months | 16 | 2213 | SMD -0.16 [-0.32, -0.00] | ⊕⊕⊝⊝  LOW | 1. D^1^  2. -  3. D^1^  4. -  5. - | overview |
|  |  | No MI comparison |  | Alcohol problems | 4+ months | 24 | 6742 | SMD -0.08 [-0.15, 0.00] | ⊕⊕⊝⊝  LOW | 1. D^1^  2. -  3. D^1^  4. -  5. - | review |
|  |  | No MI comparison |  | Average BAC | <4 months | 2 | 434 | SMD -0.11 [-0.30, 0.07] | ⊕⊕⊝⊝  LOW | 1. D^1^  2. -  3. -  4. D^1^  5. - | overview |
|  |  | No MI comparison |  | Drink driving | <4 months | 2 | 467 | SMD -0.26 [-0.44, -0.07] | ⊕⊕⊝⊝  LOW | 1. D^1^  2. D^1^  3. -  4. -  5. - | overview |
| Diet | Armstrong et al 2011[3] | Control | Overweight or obese adults | Change in body mass | unclear | 11 | 1448 | SMD -0.51 [-1.04, 0.01] | ⊕⊕⊝⊝  LOW | 1. D^1^  2. -  3. D^1^  4. -  5. - | overview |
|  |  | Control |  | Weighted change scores in BMI | unclear | 7 | NS | WMD −0.24 [−0.50, 0.01] | ⊕⊕⊝⊝  LOW | 1. D^1^  2. -  3. D^1^  4. -  5. - | overview |
|  |  | Control |  | Weighted change scores in weight (kg) | unclear | 8 | NS | WMD −1.47 [−2.05, −0.88] | ⊕⊕⊝⊝  LOW | 1. D^1^  2. -  3. D^1^  4. -  5. - | overview |
| Drinking safe water | Lundahl et al 2010[4] |  | Mixed | health-related behaviours |  | 1 | unclear | ES 0.73 [0.31, 1.15] | ⊕⊝⊝⊝  VERY LOW | 1. D^1^  2. D^1^  3. -  4. D^1^  5. - | overview |
| Drugs | Lundahl et al 2010[4] |  | Mixed | Miscellaneous drug problems |  | 27 | unclear | ES 0.08 [-0.03, 0.20] | ⊕⊕⊝⊝  LOW | 1. D^1^  2. -  3. -  4. D^1^  5. - | overview |
| Eating disorders | Lundahl et al 2010[4] |  | Mixed | Health-related behaviours |  | 1 | unclear | ES 0.18 [-0.23, 0.59] | ⊕⊝⊝⊝  VERY LOW | 1. D^1^  2. D^1^  3. -  4. D^1^  5. - | overview |
| Gambling | Cowlishaw et al 2012[5] | Control | Pathological or problem gamblers | Financial loss from gambling | 0-3 months | 3 | 244 | SMD -0.41 [ -0.75, -0.07 ] | ⊕⊝⊝⊝  VERY LOW | 1. -  2. D^1^  3. D^1^  4. D^1^  5. - | review |
|  |  | Control |  |  | 9-12 months | 2 | 152 | SMD -0.26 [ -0.58, 0.06 ] | ⊕⊝⊝⊝  VERY LOW | 1. -  2. D^1^  3. D^1^  4. D^1^  5. - | overview |
|  |  | Control |  | Frequency of gambling | 0-3 months | 2 | 145 | SMD -0.18 [ -0.50, 0.15] | ⊕⊝⊝⊝  VERY LOW | 1. -  2. D^1^  3. D^1^  4. D^1^  5. - | review |
|  |  | Control |  |  | 9-12 months | 1 | 62 | SMD -0.53 [ -1.04, -0.02 ] | ⊕⊝⊝⊝  VERY LOW | 1. -  2. D^1^  3. D^1^  4. D^1^  5. - | overview |
|  |  | Control |  | Gambling symptom severity | 0-3 months | 2 | 163 | SMD -0.03 [ -0.55, 0.50 ] | ⊕⊝⊝⊝  VERY LOW | 1. -  2. D^1^  3. D^1^  4. D^1^  5. - | overview |
|  |  | Control |  |  | 9-12 months | 3 | 221 | SMD -0.25 [ -0.61, 0.12 ] | ⊕⊝⊝⊝  VERY LOW | 1. -  2. D^1^  3. D^1^  4. D^1^  5. - | overview |
|  | Yakavenko et al 2015[6] | Control | Adult disordered gamblers | Post-treatment average days gambled | Immediate | 5 | 477 | WMD −1.30 [−2.39, −0.21] | ⊕⊕⊝⊝  LOW | 1. D^1^  2. D^1^  3. -  4. -  5. - | overview |
|  |  | Control |  | Post-treatment average dollars lost | Immediate | 5 | 477 | WMD = −0.23 [−0.42, −0.05] | ⊕⊕⊝⊝  LOW | 1. D^1^  2. D^1^  3. -  4. -  5. - | overview |
|  |  | Control |  | Short term average days gambled/month | 6 months | 3 | 310 | WMD= −1.22 [−2.06, −0.38] | ⊕⊕⊝⊝  LOW | 1. D^1^  2. D^1^  3. -  4. -  5. - | overview |
|  |  | Control |  | Short term average days gambled/month | 6 months | 3 | 310 | WMD = −0.26 [−0.53, 0.02] | ⊕⊕⊝⊝  LOW | 1. D^1^  2. D^1^  3. -  4. -  5. -- | overview |
|  |  | Control |  | Long term average days gambled/month | 9-12 months follow-up | 5 | 477 | WMD = −1.12 [−2.16, −0.07] | ⊕⊕⊝⊝  LOW | 1. D^1^  2. D^1^  3. -  4. -  5. - | overview |
|  |  | Control |  | Long term average dollars lost/month | 9-12 months follow-up | 5 | 477 | WMD = −0.33 [−0.78, 0.12] | ⊕⊕⊝⊝  LOW | 1. D^1^  2. D^1^  3. -  4. -  5. - | overview |
| HIV risk | Hettema et al 2005[7] | “all studies” | Mixed | HIV knowledge & behaviours | All | 5 | 624 | ES 0.53 [0.24, 0.81] | ⊕⊕⊝⊝  LOW | 1. D^1^  2. -  3. -  4. D^1^  5. - | overview |
| Marijuana | Gates et al 2015[8] | Inactive control | Adults with cannabis use disorder or frequent cannabis use | Reduction in cannabis use frequency |  | 4 | 612 | MD 4.45 [1.90, 7.00] | ⊕⊝⊝⊝  VERY LOW | 1. D^1^  2. D^1^  3. D^1^  4. D^1^  5. - | overview |
|  |  | Inactive control |  | Point-prevalence abstinence |  | 1 | 197 | RR 1.19 [0.43, 3.28] | ⊕⊝⊝⊝  VERY LOW | 1. D^1^  2. D^1^  3. -  4. D^1^  5. - | overview |
|  |  | Inactive control |  | Reduction in joints/day |  | 4 | 611 | SMD 3.17 [2.67, 3.66] | ⊕⊝⊝⊝  VERY LOW | 1. D^1^  2. D^1^  3. D^1^  4. D^1^  5. - | overview |
|  |  | Inactive control |  | Symptoms of dependence |  | 2 | 316 | SMD 4.07 [1.97, 6.17] | ⊕⊝⊝⊝  VERY LOW | 1. D^1^  2. D^1^  3. D^1^  4. D^1^  5. - | Overview |
|  |  | Inactive control |  | Reduction in cannabis-related problems |  | 4 | 612 | SMD 3.29 [1.85, 4.72] | ⊕⊝⊝⊝  VERY LOW | 1. D^1^  2. D^1^  3. D^1^  4. D^1^  5. - | Overview |
|  |  | DC |  | Reduction in cannabis-use frequency |  | 1 | 112 | MD 3.99 [0.89, 7.08] | ⊕⊝⊝⊝  VERY LOW | 1. D^1^  2. D^1^  3. -  4. D^1^  5. - | Overview |
|  |  | CBT |  | Reduction in cannabis-use frequency |  | 1 | 179 | MD -0.86 [-3.86, 2.14] | ⊕⊝⊝⊝  VERY LOW | 1. D^1^  2. D^1^  3. -  4. D^1^  5. - | Overview |
|  |  | MET+CBT |  | Reduction in cannabis-use frequency |  | 1 | 31 | MD -0.86 [-3.86, 2.14] | ⊕⊝⊝⊝  VERY LOW | 1. D^1^  2. D^1^  3. -  4. D^1^  5. - | Overview |
|  |  | MET+CBT+CBT-abs (EoT) |  | Reduction in cannabis-use frequency |  | 1 | 30 | -MD 7.30 [-13.68, -0.92] | ⊕⊝⊝⊝  VERY LOW | 1. D^1^  2. D^1^  3. -  4. D^1^  5. - | Overview |
|  |  | MET+CBT+CBT-abs |  | Reduction in cannabis-use frequency |  | 1 | 266 | MD -4.96 [-7.18, -2.74] | ⊕⊝⊝⊝  VERY LOW | 1. D^1^  2. D^1^  3. -  4. D^1^  5. - | Overview |
|  |  | MET+CBT |  | Point-prevalence abstinence |  | 2 | 301 | OR 3.59 [1.80, 7.20] | ⊕⊝⊝⊝  VERY LOW | 1. D^1^  2. D^1^  3. D^1^  4. D^1^  5. - | Overview |
|  |  | CBT |  | Point-prevalence abstinence |  | 1 | 170 | OR 0.80 [0.43, 1.47] | ⊕⊝⊝⊝  VERY LOW | 1. D^1^  2. D^1^  3. -  4. D^1^  5. - | Overview |
|  |  | CBT |  | Reduction in joints/day |  | 1 | 183 | SMD -1.63 [-1.97, -1.29] | ⊕⊝⊝⊝  VERY LOW | 1. D^1^  2. D^1^  3. -  4. D^1^  5. - | Overview |
|  |  | MET+CBT+CBT-abs |  | Reduction in joints/day |  | 1 | 266 | SMD 0.22 [-0.02, 0.46] | ⊕⊝⊝⊝  VERY LOW | 1. D^1^  2. D^1^  3. -  4. D^1^  5. - | Overview |
|  |  | DC |  | Reduction in joints/day |  | 1 | 101 | SMD 1.81 [1.35, 2.28] | ⊕⊝⊝⊝  VERY LOW | 1. D^1^  2. D^1^  3. -  4. D^1^  5. - | Overview |
|  |  | Drug education control |  | Reduction in symptoms of dependence |  | 1 | 101 | SMD 4.32 [3.60, 5.04] | ⊕⊝⊝⊝  VERY LOW | 1. D^1^  2. D^1^  3. -  4. D^1^  5. - | Overview |
|  |  | MET+CBT |  | Reduction in symptoms of dependence |  | 1 | 266 | SMD -1.78 [-2.07, -1.50] | ⊕⊝⊝⊝  VERY LOW | 1. D^1^  2. D^1^  3. -  4. D^1^  5. - | Overview |
|  |  | CBT |  | Reduction in symptoms of dependence |  | 1 | 183 | SMD 0.06 [-0.23, 0.36] | ⊕⊝⊝⊝  VERY LOW | 1. D^1^  2. D^1^  3. -  4. D^1^  5. - | Overview |
|  |  | MET+CBT |  | Reduction in cannabis-related problems |  | 1 | 30 | -MD 0.34 [-0.47, -0.22] | ⊕⊝⊝⊝  VERY LOW | 1. D^1^  2. D^1^  3. -  4. D^1^  5. - | Overview |
|  |  | MET+CBT+CBT-abs |  | Treatment completion |  | 1 | 302 | MD 0.04 [-0.22, 0.30] | ⊕⊝⊝⊝  VERY LOW | 1. D^1^  2. D^1^  3. -  4. D^1^  5. - | Overview |
|  |  | MET+CBT (high intensity) |  | Improvement in motivation to quit |  | 1 | 30 | MD 1.54 [1.26, 1.87] | ⊕⊝⊝⊝  VERY LOW | 1. D^1^  2. D^1^  3. -  4. D^1^  5. - | Overview |
|  |  | MET+CBT+CM-abs |  | Reduction in alcohol use severity (ASI score) |  | 2 | 280 | -MD 9.8[-25.83,6.23] | ⊕⊝⊝⊝  VERY LOW | 1. D^1^  2. D^1^  3. D^1^  4. D^1^  5. - | Overview |
|  |  | MET+CBT |  | Reduction in frequency of alcohol use |  | 1 | 249 | -MD 0.02[-0.07, 0.03] | ⊕⊝⊝⊝  VERY LOW | 1. D^1^  2. D^1^  3. -  4. D^1^  5. - | Overview |
|  |  | MET+CBT |  | Reduction in drug use severity |  | 1 | 31 | MD 11.18 [-13.43, 35.79] | ⊕⊝⊝⊝  VERY LOW | 1. D^1^  2. D^1^  3. -  4. D^1^  5. - | Overview |
|  |  | MET+CBT |  | Reduction in cannabis-related problems |  | 1 | 30 | MD 0.03 [-0.08,0.02] | ⊕⊝⊝⊝  VERY LOW | 1. D^1^  2. D^1^  3. -  4. D^1^  5. - | Overview |
|  | Lundahl et al 2010[4] |  | Mixed | Marijuana-related problems |  | 17 | unclear | ES 0.26 [0.10, 0.43] | ⊕⊕⊝⊝  LOW | 1. D^1^  2. -  3. -  4. D^1^  5. - | Overview |
|  | Lundahl et al 2013[9] |  | Patients attending general medical care settings | Marijuana: abstinence |  | 1 | unclear | OR 1.99 [0.81, 4.86] | ⊕⊕⊝⊝  LOW | 1. D^1^  2. D^1^  3. -  4. -  5. - | Overview |
|  |  |  |  | Marijuana: amount |  | 5 | unclear | OR 3.22 [2.14, 4.84] | ⊕⊕⊝⊝  LOW | 1. D^1^  2. -  3. D^1^  4. -  5. - | Overview |
| Adherence to medication | Palacio et al, 2016 | Control | Mixed | Adequate adherence  (categorical measures) | NR | 11 | unclear | RR 1.17 [1.05, 1.31] | ⊕⊝⊝⊝  VERY LOW | 1. D^1^  2. -  3. D^1^  4. D^1^  5. - | Overview |
|  |  | Control | Mixed | Adequate adherence (continuous measures) | NR | 11 | unclear | SMD 0.70 [0.15, 1.25] | ⊕⊝⊝⊝  VERY LOW | 1. D^1^  2. -  3. D^1^  4. D^1^  5. - | Overview |
|  | Lawrence et al 2017 | Control | People with “mental health issues” | Attendance to treatment | NR | 12 | 711 | OR 2.9 [2.24, 4.98] | ⊕⊕⊝⊝  LOW | 1. D^1^  2. -  3. -  4. D^1^  5. - | Overview |
|  | Zomahoun et al. 2017* | Control | Adults with chronic diseases | Medication adherence | NR | 16 | 3893 | SMD 0.12 [0.05, 0.20] | ⊕⊕⊝⊝  LOW | 1. D^1^  2. -  3. -  4. D^1^  5. - | Overview |
| Metabolic disorders | Jones et al 2014[10] | Control | People with type 1 and type 2 diabetes | Control on measures of glycated haemoglobin |  | 13 | 3154 | MD 0.17 [-.0.09, 0.43] | ⊕⊝⊝⊝  VERY LOW | 1. D^1^  2. D^1^  3. D^1^  4. D^1^  5. - | Overview |
| Management of neurovascular disorders | Cheng et al 2015[11] | UC | People with stroke | ADL | 3-month follow-up | 1 | 411 | RR 1.01 [0.84, 1.23] | ⊕⊕⊝⊝  LOW | 1. D^1^  2. D^1^  3. -  4. -  5. - | Overview |
|  |  | UC |  | ADL | 12-month follow-up | 1 | 411 | RR 1.11 [0.89, 1.37] | ⊕⊕⊝⊝  LOW | 1. D^1^  2. D^1^  3. -  4. -  5. - | Overview |
|  |  | UC |  | Mood | 3-month follow-up | 1 | 411 | RR 1.36 [1.07, 1.73] | ⊕⊕⊝⊝  LOW | 1. D^1^  2. D^1^  3. -  4. -  5. - | Overview |
|  |  | UC |  | Mood | 12-month follow-up | 1 | 411 | RR 1.35 [1.05, 1.74] | ⊕⊕⊝⊝  LOW | 1. D^1^  2. D^1^  3. -  4. -  5. - | Overview |
|  |  | UC |  | Death | 3-month follow-up | 1 | 411 | RR 0.34 [0.11, 1.03] | ⊕⊕⊝⊝  LOW | 1. D^1^  2. D^1^  3. -  4. -  5. - | Overview |
|  |  | UC |  | Death | 12-month follow-up | 1 | 411 | RR 0.53 [0.28, 1.00] | ⊕⊕⊝⊝  LOW | 1. D^1^  2. D^1^  3. -  4. -  5. - | Overview |
| Oral health | Werner et al 2016 [12] | Control | Adults with periodontitis | Gingivitis measured using bleeding on probing | NR | 3 | 200 | Bleeding on probing, MI: –2.81 [–11.54, 5.91] | ⊕⊕⊝⊝  LOW | 1. D^1^  2. -  3. D^1^  4. -  5. - | Review |
| Parenting practice | Lundahl et al 2010 [4] |  | Parents | health-related behaviours |  | 2 | Unclear | ES 0.29 [0.06, 0.53] | ⊕⊕⊝⊝  LOW | 1. D^1^  2. D^1^  3. -  4. -  5. - | Overview |
| Physical activity | O'Halloran et al 2013 [13] | Control or UC | Chronic health conditions | Cardiorespiratory fitness | Immediately | 3 | 189 | SMD –0.07 [–0.56, 0.43] | ⊕⊝⊝⊝  VERY LOW | 1. -  2. D^1^  3. D^1^  4. D^1^  5. - | Review |
| Sexual health | Berg et al 2011[14] |  | Men who have sex with men | Condom use | unclear | 2 | 287 | SMD -0.05 [-0.28, 0.18] | ⊕⊕⊝⊝  LOW | 1. D^1^  2. D^1^  3. -  4. -  5. - | Overview |
|  |  |  |  | Drinks consumed/day | short term | 2 | 291 | SMD -0.34 [-0.58, -0.11 ] | ⊕⊕⊝⊝  LOW | 1. D^1^  2. D^1^  3. -  4. -  5. - | Overview |
|  |  |  |  |  | long term | 2 | 268 | SMD -0.15 [-0.39, 0.09 ] | ⊕⊕⊝⊝  LOW | 1. D^1^  2. D^1^  3. -  4. -  5. - | Overview |
|  |  |  |  | Unprotected anal intercourse | short term | 3 | 499 | SMD 0.03 [-0.14, 0.21] | ⊕⊕⊝⊝  LOW | 1. D^1^  2. D^1^  3. -  4. -  5. - | Overview |
|  |  |  |  | Unprotected anal intercourse (UAI) with primary partner | unclear | 2 | 553 | RR 1.34 [0.61, 2.95] | ⊕⊕⊝⊝  LOW | 1. D^1^  2. -  3. D^1^  4. -  5. - | overview |
|  | Lopez et al 2013 [15] |  | Women who were users or potential users of contraception | Contraceptive use maintained at high level or improved | at 12 months | 1 | 664 | OR 1.19 [ 0.87, 1.63 ] | ⊕⊕⊝⊝  LOW | 1. D^1^  2. D^1^  3. -  4. -  5. - | Overview |
|  |  |  |  |  | at 2 months | 1 | 648 | OR 1.33 [ 0.95, 1.85 ] | ⊕⊕⊝⊝  LOW | 1. D^1^  2. D^1^  3. -  4. -  5. - | Overview |
|  |  |  |  | Effective contraceptive use (at 3 months). | at 3 months | 1 | 665 | OR 2.12 [ 1.53, 2.92 ] | ⊕⊕⊝⊝  LOW | 1. D^1^  2. D^1^  3. -  4. -  5. - | Overview |
|  |  |  |  |  | at 9 months | 1 | 593 | OR 2.04 [ 1.47, 2.83 ] | ⊕⊕⊝⊝  LOW | 1. D^1^  2. D^1^  3. -  4. -  5. - | overview |
|  |  |  |  | Ineffective contraceptive use | at 1 month | 1 | 199 | OR 0.49 [ 0.28, 0.87 ] | ⊕⊕⊝⊝  LOW | 1. D^1^  2. D^1^  3. -  4. -  5. - | overview |
|  |  |  |  |  | at 4 months | 1 | 207 | OR 0.56 [ 0.31, 0.98 ] | ⊕⊕⊝⊝  LOW | 1. D^1^  2. D^1^  3. -  4. -  5. - | overview |
|  |  |  |  | Pregnancy (by 12 months) | at 12 months | 1 | 737 | OR 0.88 [ 0.55, 1.42 ] | ⊕⊕⊝⊝  LOW | 1. D^1^  2. D^1^  3. -  4. -  5. - | overview |
|  | Wilson et al 2015 [16] | UC | High risk of unintended pregnancy | Effective contraceptive use | 0-4 months | 6 | 1233 | RR 1.32 [1.11, 1.56] | ⊕⊝⊝⊝  VERY LOW | 1. D^1^  2. D^1^  3. D^1^  4. -  5. - | overview |
|  |  | UC |  | Effective contraceptive use | 4- 8 months post intervention | 4 | 1333 | RR 1.10 [0.93, 1.32] | ⊕⊕⊝⊝  LOW | 1. D^1^  2. -  3. D^1^  4. -  5. - | overview |
|  |  | UC |  | Effective contraceptive use | 8-12 months post intervention | 4 | 1272 | RR 1.18 [0.96, 1.46] | ⊕⊝⊝⊝  VERY LOW | 1. D^1^  2. D^1^  3. D^1^  4. -  5. - | overview |
|  |  | UC |  | Effective contraceptive use | 12-24 months post-intervention | 2 | 440 | RR 0.80 [0.51, 1.26] | ⊕⊕⊝⊝  LOW | 1. D^1^  2. D^1^  3. -  4. -  5. - | overview |
| Smoking | Rabe et al 2013 [17] |  | In Emergency department | Cessation | unclear | 5 | 1755 | RR 1.33 (0.92, 1.92) | ⊕⊕⊝⊝  LOW | 1. D^1^  2. -  3. D^1^  4. -  5. - | overview |
|  | Hettema et al 2010 [18] |  | Pregnant women | Cessation | Shortest follow-up | 7 | unclear | ES -0.01 [-0.17, 0.15] | ⊕⊕⊝⊝  LOW | 1. D^1^  2. -  3. -  4. D^1^  5. - | overview |
|  |  |  |  | Cessation | Longest follow-up | 2 | unclear | ES 0.15 [ -0.19, 0.49] | ⊕⊝⊝⊝  VERY LOW | 1. D^1^  2. D^1^  3. -  4. D^1^  5. - | overview |
| Substance abuse | Darker 2015 [19] | UC | Patients with BZD harmful use, abuse or dependence | Successful discontinuation of BZDs | Post-treatment | 2 | 34 | RR 4.43 [0.16, 125.35] | ⊕⊝⊝⊝  VERY LOW | 1. D^1^  2. D^1^  3. D^1^  4. D^1^  5. - | review |
|  |  | UC |  | Successful discontinuation of BZDs | 3 month follow up | 4 | 80 | RR 3.46 [0.53, 22.45] | ⊕⊝⊝⊝  VERY LOW | 1. D^1^  2. D^1^  3. D^1^  4. D^1^  5. - | review |
|  |  | UC |  | Reduction of BZD > 50 % | 3 month follow up | 1 | 39 | RR 1.52 [0.60, 3.83] | ⊕⊝⊝⊝  VERY LOW | 1. D^1^  2. D^1^  3. -  4. D^1^  5. - | overview |
|  |  | UC |  | Reduction of BZD > 50 % | 12 month follow up | 1 | 39 | RR 0.87 [0.52, 1.47] | ⊕⊝⊝⊝  VERY LOW | 1. D^1^  2. D^1^  3. -  4. D^1^  5. - | overview |
|  | Lundahl et al 2013 [9] |  | Patients attending general medical care settings | alcohol amount |  | 9 | unclear | OR 2.31 [1.75, 3.06] | ⊕⊕⊝⊝  LOW | 1. D^1^  2. -  3. D^1^  4. -  5. - | overview |
|  |  |  |  | Alcohol: dangerous use |  | 4 | unclear | OR 1.83 [1.33, 2.53] | ⊕⊕⊝⊝  LOW | 1. D^1^  2. -  3. D^1^  4. -  5. - | overview |
|  |  |  |  | Smoking tobacco: abstinence |  | 8 | unclear | OR 1.34 [1.05, 1.70] | ⊕⊕⊝⊝  LOW | 1. D^1^  2. -  3. D^1^  4. -  5. - | overview |
|  |  |  |  | Smoking tobacco: amount |  | 4 | unclear | OR 1.18 [0.96, 1.45] | ⊕⊕⊝⊝  LOW | 1. D^1^  2. -  3. D^1^  4. -  5. - | overview |
|  | Smedslund et al 2011 [20] | No intervention | People with substance abuse, dependency or addiction | Extent of substance use | Post-intervention | 4 | 202 | SMD 0.79 [0.48, 1.09] | ⊕⊕⊝⊝  LOW | 1. D^1^  2. D^1^  3. -  4. -  5. - | review |
|  |  | No intervention |  |  | Medium follow up | 12 | 2326 | SMD 0.15 [0.04, 0.25] | ⊕⊕⊝⊝  LOW | 1. D^1^  2. D^1^  3. -  4. -  5. - | review |
|  |  | No intervention |  |  | Long follow-up | 1 | 363 | SMD 0.06 [-0.16, 0.28] | ⊕⊕⊝⊝  LOW | 1. D^1^  2. D^1^  3. -  4. -  5. - | review |
|  |  | UC |  | Extent of substance use | Medium follow up | 5 | 890 | SMD 0.08 [-0.05, 0.21] | ⊕⊕⊝⊝  LOW | 1. D^2^  2. D^1^  3. -  4. -  5. - | review |
|  |  | Other active intervention |  | Extent of substance use | Post-intervention | 2 | 185 | SMD -0.07 [-0.37, 0.23] | ⊕⊕⊝⊝  LOW | 1. D^2^  2. D^1^  3. -  4. -  5. - | review |
|  |  | Other active intervention |  |  | Long follow-up | 2 | 437 | SMD -0.03 [-0.21, 0.14] | ⊕⊕⊝⊝  LOW | 1. D^2^  2. -  3. D^1^  4. -  5. - | review |
|  |  | Assessment and feedback |  | Extent of substance use | Short follow-up | 7 | 986 | SMD 0.12 [-0.01, 0.24] | ⊕⊕⊝⊝  LOW | 1. D^1^  2. -  3. D^1^  4. -  5. - | review |
|  |  | Assessment and feedback |  |  | medium follow up | 2 | 265 | SMD 0.38 [0.10, 0.66] | ⊕⊕⊝⊝  LOW | 1. D^1^  2. -  3. D^1^  4. -  5. - | review |
|  |  | No intervention |  | Readiness to change |  | 5 | 1495 | SMD 0.05 [-0.11, 0.22] | ⊕⊕⊝⊝  LOW | 1. D^1^  2. -  3. D^1^  4. -  5. - | review |
|  |  | Other active intervention |  | Readiness to change |  | 2 | 350 | SMD -0.03 [-0.24, 0.18] | ⊕⊕⊝⊝  LOW | 1. D^1^  2. -  3. D^1^  4. -  5. - | review |
|  |  | UC |  | Retention in treatment | (0-12 months) | 4 | 1354 | SMD -0.11 [-0.41, 0.19] | ⊕⊝⊝⊝  VERY LOW | 1. D^1^  2. D^1^  3. D^1^  4. -  5. - | review |
|  |  | No intervention |  | Retention in treatment | 0-3 months | 2 | 427 | SMD 0.26 [-0.00, 0.52] | ⊕⊝⊝⊝  VERY LOW | 1. D^1^  2. D^1^  3. D^1^  4. -  5. - | review |
|  |  | Other active intervention |  | Retention in treatment |  | 5 | 447 | SMD 0.01 [-0.45, 0.47] | ⊕⊕⊝⊝  LOW | 1. D^1^  2. -  3. D^1^  4. -  5. - | review |
|  | Terplan et al. 2015 [21] | control (maternal outcomes) |  | Positive urine drug test | Immediate | 2 | 278 | RR 0.96 [0.63, 1.48] | ⊕⊕⊝⊝  LOW | 1. D^1^  2. D^1^  3.  4. -  5. - | Overview |
|  | Terplan et al. 2015 [21] | control (maternal outcomes) |  | Positive urine drug test | 3-month follow-up | 1 | 159 | RR 1.13 [0.55, 2.31] | ⊕⊝⊝⊝  VERY LOW | 1. D^1^  2. D^1^  3.  4. -  5. - | Overview |
|  | Terplan et al. 2015[21] | control (maternal outcomes) |  | Positive urine at delivery |  | 1 | 128 | RR 0.84 [0.57, 1.24] | ⊕⊝⊝⊝  VERY LOW | 1. D^1^  2. D^1^  3.  4. -  5. - | Overview |
|  | Terplan et al. 2015 [21] | control (maternal outcomes) |  | Retention at treatment completion |  | 3 | 355 | RR 0.97 [0.89, 1.06] | ⊕⊕⊝⊝  LOW | 1. D^2^  2. -  3. -  4. -  5. - | Review |
|  | Terplan et al. 2015 [21] | control (maternal outcomes) |  | Short-term treatment retention |  | 3 | 334 | RR 0.99 [0.88, 1.12] | ⊕⊕⊝⊝  LOW | 1. D^1^  2. -  3. D^1^  4. -  5. - | Overview |
| Treatment compliance | Hettema et al 2005[7] | Unclear | Mixed | Treatment compliance | across all follow-up points | 5 | unclear | ES 0.72 [0.56, 0.89] | ⊕⊝⊝⊝  VERY LOW | 1. D^1^  2. -  3. D^1^  4. D^1^  5. - | overview |
|  | Lundahl et al 2013[9] | Unclear | Patients attending general medical care settings | Breastfeeding |  | 1 | unclear | OR 1.48 [0.73, 3.01] | ⊕⊕⊝⊝  LOW | 1. D^1^  2. D^1^  3. -  4. -  5. - | overview |
|  |  | Unclear |  | Medication adherence |  | 4 | unclear | OR 1.25 [0.95, 1.65] | ⊕⊕⊝⊝  LOW | 1. D^1^  2. -  3. -  4. D^1^  5. - | overview |
|  |  | Unclear |  | Sedentary behaviour |  | 5 | unclear | OR 1.47 [1.19, 1.81] | ⊕⊕⊝⊝  LOW | 1. D^1^  2. -  3. D^1^  4. D^1^  5. - | overview |
|  |  | Unclear |  | Self-care |  | 2 | unclear | OR 0.64 [0.33, 1.27] | ⊕⊕⊝⊝  LOW | 1. D^1^  2. -  3. -  4. D^1^  5. - | overview |
|  |  | Unclear |  | Self-monitoring |  | 4 | unclear | OR 2.14 [1.65, 2.79] | ⊕⊝⊝⊝  VERY LOW | 1. D^1^  2. -  3. D^1^  4. D^1^  5. - | overview |
| All combined | Lundahl et al 2013[9] | Unclear | Patients attending general medical care settings | Overall effectiveness |  | 51 | unclear | OR 1.55 [1.40, 1.71] | ⊕⊝⊝⊝  VERY LOW | 1. D^1^  2. -  3. D^1^  4. D^1^  5. - | overview |
|  | Lundahl et al 2010[4] | any other control | Various | Overall effectiveness |  | 132 | unclear | ES 0.22 [0.17, 0.27] | ⊕⊝⊝⊝  VERY LOW | 1. D1  2. -  3. D1  4. D1  5. - | (overview) |
|  | Van Buskirk et al 2014 [22] | Control | Patients attending primary care settings | All outcomes |  | 16 | unclear | ES 0.18 [0.03, 0.33] | ⊕⊕⊝⊝  LOW | 1. D^1^  2. -  3. D^1^  4. D^1^  5. - | overview |
|  |  | Control |  | Adherence |  | 2 | unclear | ES 0.19 [0.01, 0.37] | ⊕⊝⊝⊝  VERY LOW | 1. D^1^  2. D^1^  3. -  4. D^1^  5. - | overview |
|  |  | Control |  | Blood pressure |  | 3 | unclear | ES 0.38 [-.024, 0.31] | ⊕⊝⊝⊝  VERY LOW | 1. D^1^  2. D^1^  3. D^1^  4. D^1^  5. - | overview |
|  |  | Control |  | Body weight reduction |  | 2 | unclear | ES 0.47 [-0.04, 0.99] | ⊕⊝⊝⊝  VERY LOW | 1. D^1^  2. D^1^  3. D^1^  4. D^1^  5. - | overview |
|  |  | Control |  | Physical activity |  | 3 | unclear | ES 0.07 [-0.08, 0.21] | ⊕⊝⊝⊝  VERY LOW | 1. D^1^  2. D^1^  3. -  4. D^1^  5. - | overview |
|  |  | Control |  | Substance use |  | 6 | unclear | ES 0.22 [-0.21, 0.65] | ⊕⊝⊝⊝  VERY LOW | 1. D^1^  2. D^1^  3. D^1^  4. D^1^  5. - | overview |
|  | Alperstein (2016) [23] | Inactive control | Patients with chronic pain | Adherence to treatment | Post treatment | 5 | 631 | ES 0.44 0.08,0.80] | ⊕⊕⊝⊝  LOW | 1. D^1^  2. -  3. D^1^  4. -  5. - | overview |
|  |  | Inactive control |  | Pain intensity | Post treatment | 4 | 449 | ES 0.27 [0.04, 0.50] | ⊕⊕⊝⊝  LOW | 1. D^1^  2. -  3. D^1^  4. -  5. - | overview |
|  |  | Inactive control |  | Physical function | 6 months | 5 | 779 | ES 0.12 [-0.02,0.26] | ⊕⊕⊝⊝  LOW | 1. D^1^  2. -  3. -  4. D^1^  5. - | overview |

**S3 TABLE. Summary of comparisons judged to provide low or very low quality evidence**

Abbreviations: ADL: activities of daily living; BDZ: benzodiazepines; BMI: body mass index; ES: effect size; NR: not reported; NT: no treatment; OR: odds ration; RR: risk ratio; SMD: standardised mean difference; UC: usual care; WMD: weighted mean difference

Reasons for downgrading evidence: 1 = serious limitation in the Risk of bias; 2 = imprecision (e.g. wide confidence intervals or small sample size); 3 = Inconsistency (e.g. high I^2^); 4 = indirectness (e.g. variation in participants, intervention, comparisons or outcomes); 5 = publication bias; D^1^ = one downgrade; D2 = two downgrades

GRADE Working Group grades of evidence
**High quality**: Further research is very unlikely to change our confidence in the estimate of effect.
**Moderate quality**: Further research is likely to have an important impact on our confidence in the estimate of effect and may change the estimate.
**Low quality**: Further research is very likely to have an important impact on our confidence in the estimate of effect and is likely to change the estimate.
**Very low quality**: We are very uncertain about the estimate.

We have applied GRADE to the sensitivity data analysis reported in the paper. Authors explored heterogeneity and removed one study with wide confidence intervals and although the effect size decreased from SMD 0.23 [0.08, 0.37] to SMD 0.12 [0.05, 0.20], the I^2^ improved (from 59% to 1%). Consequently, we improved the GRADE judgement from very low (n=17 studies) to low (n=16)

**References**

1. Vasilaki EI, Hosier SG, Cox WM. The efficacy of motivational interviewing as a brief intervention for excessive drinking: A meta-analytic review. Alcohol and Alcoholism. 2006;41(3):328-35. PubMed PMID: 2006-05794-019.

2. Foxcroft DR, Coombes L, Wood S, Allen D, Almeida Santimano NML. Motivational interviewing for alcohol misuse in young adults. The Cochrane database of systematic reviews. 2014;8:CD007025.

3. Armstrong MJ, Mottershead TA, Ronksley PE, Sigal RJ, Campbell TS, Hemmelgarn BR. Motivational interviewing to improve weight loss in overweight and/or obese patients: a systematic review and meta-analysis of randomized controlled trials. Obes Rev. 2011;12(9):709-23. doi: 10.1111/j.1467-789X.2011.00892.x. PubMed PMID: 21692966.

4. Lundahl BW, Kunz C, Brownell C, Tollefson D, Burke BL. A meta-analysis of motivational interviewing: twenty-five years of empirical studies (Provisional abstract). Research on Social Work Practice [Internet]. 2010; 20(2):[137-60 pp.]. Available from: http://onlinelibrary.wiley.com/o/cochrane/cldare/articles/DARE-12010003750/frame.html.

5. Cowlishaw S, Merkouris S, Dowling N, Anderson C, Jackson A, Thomas S. Psychological therapies for pathological and problem gambling. Cochrane Database of Systematic Reviews [Internet]. 2012; (11). Available from: http://onlinelibrary.wiley.com/doi/10.1002/14651858.CD008937.pub2/abstracthttp://onlinelibrary.wiley.com/store/10.1002/14651858.CD008937.pub2/asset/CD008937.pdf?v=1&t=i2qmh2a4&s=24442e6c86853f078ee853bb6912770fd69af25b.

6. Yakovenko I, Quigley L, Hemmelgarn BR, Hodgins DC, Ronksley P. The efficacy of motivational interviewing for disordered gambling: systematic review and meta-analysis. Addict Behav. 2015;43:72-82. doi: 10.1016/j.addbeh.2014.12.011. PubMed PMID: 25577724.

7. Hettema J, Steele J, Miller WR. Motivational interviewing. Annu Rev Clin Psychol. 2005;1:91-111. doi: 10.1146/annurev.clinpsy.1.102803.143833. PubMed PMID: 17716083.

8. Gates P, J, Sabioni P, Copeland J, Le Foll B, Gowing L. Psychosocial interventions for cannabis use disorder. Cochrane Database of Systematic Reviews [Internet]. 2016; (5). Available from: http://onlinelibrary.wiley.com/doi/10.1002/14651858.CD005336.pub4/abstract; http://onlinelibrary.wiley.com/store/10.1002/14651858.CD005336.pub4/asset/CD005336.pdf?v=1&t=ioig2jkq&s=3973c2abe787bfb16f223d7cc0f1339d4e6d1ba4.

9. Lundahl B, Moleni T, Burke BL, Butters R, Tollefson D, Butler C, et al. Motivational interviewing in medical care settings: a systematic review and meta-analysis of randomized controlled trials (Structured abstract). Patient Education and Counseling [Internet]. 2013; 93(2):[157-68 pp.]. Available from: http://onlinelibrary.wiley.com/o/cochrane/cldare/articles/DARE-12013052286/frame.htmlhttp://www.pec-journal.com/article/S0738-3991(13)00288-7/pdf.

10. Jones A, Gladstone BP, Lubeck M, Lindekilde N, Upton D, Vach W. Motivational interventions in the management of HbA1c levels: a systematic review and meta-analysis (Provisional abstract). Database of Abstracts of Reviews of Effects [Internet]. 2014; (2):[91-100 pp.]. Available from: http://onlinelibrary.wiley.com/o/cochrane/cldare/articles/DARE-12014015299/frame.html.

11. Cheng D, Qu Z, Huang J, Xiao Y, Luo H, Wang J. Motivational interviewing for improving recovery after stroke. Cochrane Database Syst Rev. 2015;6(6):CD011398. doi: 10.1002/14651858.CD011398.pub2. PubMed PMID: 26037617.

12. Werner H, Hakeberg M, Dahlstrom L, Eriksson M, Sjogren P, Strandell A, et al. Psychological Interventions for Poor Oral Health: A Systematic Review. Journal of dental research. 2016;95(5):506-14. doi: https://dx.doi.org/10.1177/0022034516628506.

13. O’Halloran PD, Blackstock F, Shields N, Holland A, Iles R, Kingsley M, et al. Motivational interviewing to increase physical activity in people with chronic health conditions: A systematic review and meta-analysis. Clinical Rehabilitation. 2014;28(12):1159-71. doi: 10.1177/0269215514536210. PubMed PMID: 2014-48880-002.

14. Berg RC, Ross MW, Tikkanen R. The effectiveness of MI4MSM: How useful is motivational interviewing as an HIV risk prevention program for men who have sex with men? A systematic review. AIDS Education and Prevention. 2011;23(6):533-49. PubMed PMID: 2011-29905-005.

15. Lopez Laureen M, Steiner M, Grimes David A, Hilgenberg D, Schulz Kenneth F. Strategies for communicating contraceptive effectiveness. Cochrane Database of Systematic Reviews [Internet]. 2013; (4). Available from: http://onlinelibrary.wiley.com/doi/10.1002/14651858.CD006964.pub3/abstract.

16. Wilson A, Nirantharakumar K, Truchanowicz EG, Surenthirakumaran R, MacArthur C, Coomarasamy A. Motivational interviews to improve contraceptive use in populations at high risk of unintended pregnancy: a systematic review and meta-analysis. European journal of obstetrics, gynecology, and reproductive biology. 2015;191:72-9.

17. Rabe GL, Wellmann J, Bagos P, Busch MA, Hense H-W, Spies C, et al. Efficacy of emergency department–initiated tobacco control—Systematic review and meta-analysis of randomized controlled trials. Nicotine & Tobacco Research. 2013;15(3):643-55. PubMed PMID: 2013-09864-004.

18. Hettema JE, Hendricks PS. Motivational interviewing for smoking cessation: a meta-analytic review (Structured abstract). Journal of Consulting and Clinical Psychology [Internet]. 2010; 78(6):[868-84 pp.]. Available from: http://onlinelibrary.wiley.com/o/cochrane/cldare/articles/DARE-12011000298/frame.html.

19. Darker CD, Sweeney BP, Barry JM, Farrell MF, Donnelly-Swift E. Psychosocial interventions for benzodiazepine harmful use, abuse or dependence. The Cochrane database of systematic reviews. 2015;5:CD009652.

20. Smedslund GRC, Hammerstrøm Karianne T, Steiro AK, A., Dahl Helene MK, Kjetil. Motivational interviewing for substance abuse. Cochrane Database of Systematic Reviews [Internet]. 2011; (5). Available from: http://onlinelibrary.wiley.com/doi/10.1002/14651858.CD008063.pub2/abstracthttp://onlinelibrary.wiley.com/store/10.1002/14651858.CD008063.pub2/asset/CD008063.pdf?v=1&t=i2qmfc7p&s=9cd557e022d5878b20c45f506b715404da5f5945.

21. Terplan M, Ramanadhan S, Locke A, Longinaker N, Lui S. Psychosocial interventions for pregnant women in outpatient illicit drug treatment programs compared to other interventions. Cochrane Database of Systematic Reviews [Internet]. 2015; (4). Available from: http://onlinelibrary.wiley.com/doi/10.1002/14651858.CD006037.pub3/abstract; http://onlinelibrary.wiley.com/store/10.1002/14651858.CD006037.pub3/asset/CD006037.pdf?v=1&t=ioig2s9v&s=fbc50772d8ebf81e1c60cf130a7a926f1b16f906.

22. VanBuskirk KAL, Wetherell J. Motivational interviewing with primary care populations: a systematic review and meta-analysis (Provisional abstract). Journal of Behavioral Medicine [Internet]. 2014; 37(4):[768-80 pp.]. Available from: http://onlinelibrary.wiley.com/o/cochrane/cldare/articles/DARE-12013048261/frame.html

http://www.ncbi.nlm.nih.gov/pmc/articles/PMC4118674/pdf/nihms587843.pdf.

23. Alperstein D, Sharpe L. The Efficacy of Motivational Interviewing in Adults With Chronic Pain: A Meta-Analysis and Systematic Review. J Pain. 2016;17(4):393-403. doi: 10.1016/j.jpain.2015.10.021. PubMed PMID: 26639413.
