# Supplementary material for: Effectiveness of Motivational Interviewing on adult behaviour change in health and social care settings: A systematic review of reviews
Source: PLoS One. 2018 Oct 18;13(10):e0204890. doi: 10.1371/journal.pone.0204890 (PMC6193639; doi:10.1371/journal.pone.0204890)
Supplement: S1 File — (DOCX) [file pone.0204890.s005.docx]

**S4 Tables A-C Exploration of moderator variables**

**Table A Comparison: MI for smoking cessation**

Review: Lindson-Hawley et al (2015) [1]

| **Subgroup explored** | **Subgroup** | **Number of studies** | **n (total)** | **Risk Ratio (M-H, Fixed, 95% CI)** | **Effect?** |
| --- | --- | --- | --- | --- | --- |
| None | all subgroups | 28 | 16803 | 1.26 [1.16, 1.36] | beneficial |
| Type of therapist delivering MI | GP | 2 | 736 | 3.49 [1.53, 7.94] | beneficial |
|  | Nurse | 5 | 2256 | 1.24 [0.91, 1.68] | no benefit or harm |
|  | Counsellor | 22 | 13593 | 1.25 [1.15, 1.36] | beneficial |
| Length of session | < 20 minutes | 9 | 3651 | 1.69 [1.34, 2.12] | beneficial |
|  | > 20 minutes | 16 | 10306 | 1.20 [1.08, 1.32] | beneficial |
| Number of sessions | 1 session | 16 | 12103 | 1.26 [1.15, 1.40] | beneficial |
|  | 2 or more sessions | 11 | 3928 | 1.20 [1.02, 1.42] | beneficial |
| Number of follow-up phone calls | 0 follow-up phone calls | 10 | 3927 | 1.41 [1.20, 1.65] | beneficial |
|  | 1-2 follow-up phone-calls | 8 | 3895 | 1.28 [1.05, 1.55] | beneficial |
|  | >2 follow-up phone calls | 8 | 8541 | 1.20 [1.07, 1.34] | beneficial |

**Table B Comparison: MI for alcohol-related outcomes in young people (<25 years)**

Review: Foxcroft et al (2014)[2]

| Outcome /  Subgroup explored | Subgroup | **Number of studies** | **n (total)** | **Standardised mean difference (95% CI)** | **Effect?** |
| --- | --- | --- | --- | --- | --- |
| Quantity of alcohol consumed (< 4months follow-up) | none (any non MI comparison) | 22 | 2677 | -0.25 [-0.37, -0.14] | beneficial |
| Type of control group | comparison with alternative intervention | 8 | 1698 | -0.15 [-0.31, 0.01] | no benefit or harm |
|  | comparison with assessment only | 14 | 979 | -0.35 [-0.48, -0.22] | beneficial |
| Quantity of alcohol consumed (> 4 months follow-up) | none (any non MI comparison) | 28 | 6676 | -0.14 [-0.20, -0.08] | beneficial |
| Type of control group | comparison with alternative intervention | 14 | 3409 | -0.16 [-0.24, -0.08] | beneficial |
|  | comparison with assessment only | 14 | 3267 | -0.11 [-0.19, -0.04] | beneficial |
| Frequency of alcohol consumption (<4 month follow-up) | none (any non MI comparison) | 15 | 1928 | -0.26 [-0.44, -0.09] | beneficial |
| Type of control group | comparison with alternative intervention | 5 | 1247 | -0.07 [-0.28, 0.14] | no benefit or harm |
|  | comparison with assessment only | 10 | 681 | -0.45 [-0.64, -0.26] | beneficial |
| Frequency of alcohol consumption (>4 months follow-up) | none (any non MI comparison) | 16 | 4390 | -0.11 [-0.19, -0.03] | beneficial |
| Type of control group | comparison with alternative intervention | 9 | 2585 | -0.11 [-0.22, -0.01] | beneficial |
|  | comparison with assessment only | 7 | 1805 | -0.11 [-0.27, 0.04] | beneficial |
| Binge drinking (<4 months follow-up) | none (any non MI comparison) | 11 | 1340 | -0.23 [-0.42, -0.04] | beneficial |
| Type of control group | comparison with alternative intervention | 5 | 999 | -0.10 [-0.32, 0.12] | beneficial |
|  | comparison with assessment only | 6 | 401 | -0.39 [-0.69, -0.08] | beneficial |
| Binge drinking (4+ months follow-up) | none (any non MI comparison) | 16 | 4028 | -0.05 [-0.12, 0.01] | beneficial |
| Type of control group | comparison with alternative intervention | 9 | 2086 | -0.09 [-0.18, -0.00] | beneficial |
|  | comparison with assessment only | 7 | 1942 | -0.01 [-0.13, 0.10] | beneficial |
| Peak BAC (>4 months follow-up) | none (any non MI comparison) | 9 | 2042 | -0.14 [-0.23, -0.05] | beneficial |
| Type of control group | comparison with alternative intervention | 5 | 1151 | -0.15 [-0.27, -0.04] | beneficial |
|  | comparison with assessment only | 4 | 891 | -0.11 [-0.25, 0.03] | beneficial |
| Alcohol problems (<4 months follow-up) | none (any non MI comparison) | 16 | 2213 | -0.16 [-0.32, -0.00] | beneficial |
| Type of control group | comparison with alternative intervention | 6 | 1280 | -0.07 [-0.37, 0.23] | no benefit or harm |
|  | comparison with assessment only | 10 | 933 | -0.22 [-0.39, -0.05] | beneficial |
| Alcohol problems (4+ months follow-up) | none (any non MI comparison) | 24 | 6742 | -0.08 [-0.15, 0.00] | beneficial |
| Type of control group | comparison with alternative intervention | 13 | 3745 | -0.06 [-0.18, 0.06] | no benefit or harm |
|  | comparison with assessment only | 11 | 2997 | -0.10 [-0.20, -0.01] | beneficial |

**Table C Comparison: MI for reducing alcohol consumption versus no-treatment control groups**

Review: Vasilaki et al (2006)[3]

| Subgroup explored | Subgroup | **Number of studies** | **n (total)** | **Effect Size** | **Effect?** |
| --- | --- | --- | --- | --- | --- |
| Reducing alcohol consumption | none | 9 | 1587 | 0.18 [0.07, 0.29] | Beneficial |
| Time of follow-up | ≤3 months follow-up | 5 | ns | 0.60 [0.36, 0.83] | Beneficial |
|  | ≤6 months follow-up | 4 | ns | 0.06 [–0.06, 0.18] | No benefit or harm |

# Reference

1. Lindson-Hawley N, Thompson TP, Begh R. Motivational interviewing for smoking cessation. Cochrane Database Syst Rev. 2015;3:CD006936. doi: 10.1002/14651858.CD006936.pub3. PubMed PMID: 25726920.

2. Foxcroft DR, Coombes L, Wood S, Allen D, Almeida Santimano NML. Motivational interviewing for alcohol misuse in young adults. The Cochrane database of systematic reviews. 2014;8:CD007025.

3. Vasilaki EI, Hosier SG, Cox WM. The efficacy of motivational interviewing as a brief intervention for excessive drinking: A meta-analytic review. Alcohol and Alcoholism. 2006;41(3):328-35. PubMed PMID: 2006-05794-019.
