# Supplementary material for: Effectiveness of Motivational Interviewing on adult behaviour change in health and social care settings: A systematic review of reviews
Source: PLoS One. 2018 Oct 18;13(10):e0204890. doi: 10.1371/journal.pone.0204890 (PMC6193639; doi:10.1371/journal.pone.0204890)
Supplement: S1 Appendix — (DOCX) [file pone.0204890.s006.docx]

# Medline (Ovid gateway) search

1. Interview, Psychological/

2. Feedback, Psychological/

3. (interview$ or feedback$ or enhancement).tw

4. or/1-3

5. Motivation/

6. Motivational$.tw.

7. MI.tw.

8. MET.tw.

9. or/5-9

10. 4 and 9

We used the search filter for identifying systematic reviews as documented in the paper.
